# Mobile App-Based Interventions to Support Diabetes Self-Management: A Systematic Review of Randomized Controlled Trials to Identify Functions Associated with Glycemic Efficacy

**DOI:** 10.2196/mhealth.6522

**Published:** 2017-03-14

**Authors:** Yuan Wu, Xun Yao, Giacomo Vespasiani, Antonio Nicolucci, Yajie Dong, Joey Kwong, Ling Li, Xin Sun, Haoming Tian, Sheyu Li

**Affiliations:** ^1^ Department of Endocrinology and Metabolism West China Hospital Sichuan University Chengdu China; ^2^ Department of Academic Affairs West China School of Medicine Sichuan University Chengdu China; ^3^ Diabetes Unit Madonna del Soccorso Hospital San Benedetto del Tronto (AP) Italy; ^4^ Center for Outcomes Research and Clinical Epidemiology Pescara Italy; ^5^ Chinese Evidence-Based Medicine Center West China Hospital Sichuan University Chengdu China

**Keywords:** mobile health, mHealth, mobile applications, mobile apps, diabetes mellitus, classification

## Abstract

**Background:**

Mobile health apps for diabetes self-management have different functions. However, the efficacy and safety of each function are not well studied, and no classification is available for these functions.

**Objective:**

The aims of this study were to (1) develop and validate a taxonomy of apps for diabetes self-management, (2) investigate the glycemic efficacy of mobile app-based interventions among adults with diabetes in a systematic review of randomized controlled trials (RCTs), and (3) explore the contribution of different function to the effectiveness of entire app-based interventions using the taxonomy.

**Methods:**

We developed a 3-axis taxonomy with columns of clinical modules, rows of functional modules and cells of functions with risk assessments. This taxonomy was validated by reviewing and classifying commercially available diabetes apps. We searched MEDLINE, EMBASE, the Cochrane Central Register of Controlled Trials, the Chinese Biomedical Literature Database, and ClinicalTrials.gov from January 2007 to May 2016. We included RCTs of adult outpatients with diabetes that compared using mobile app-based interventions with usual care alone. The mean differences (MDs) in hemoglobin A_1c_ (HbA_1c_) concentrations and risk ratios of adverse events were pooled using a random-effects meta-analysis. After taxonomic classification, we performed exploratory subgroup analyses of the presence or absence of each module across the included app-based interventions.

**Results:**

Across 12 included trials involving 974 participants, using app-based interventions was associated with a clinically significant reduction of HbA_1c_ (MD 0.48%, 95% CI 0.19%-0.78%) without excess adverse events. Larger HbA_1c_ reductions were noted among patients with type 2 diabetes than those with type 1 diabetes (MD 0.67%, 95% CI 0.30%-1.03% vs MD 0.37%, 95% CI –0.12%-0.86%). Having a complication prevention module in app-based interventions was associated with a greater HbA_1c_ reduction (with complication prevention: MD 1.31%, 95% CI 0.66%-1.96% vs without: MD 0.38%, 95% CI 0.09%-0.67%; intersubgroup *P*=.01), as was having a structured display (with structured display: MD 0.69%, 95% CI 0.32%-1.06% vs without: MD 0.69%, 95% CI –0.18%-0.53%; intersubgroup *P*=.03). However, having a clinical decision-making function was not associated with a larger HbA_1c_ reduction (with clinical decision making: MD 0.19%, 95% CI –0.24%-0.63% vs without: MD 0.61%, 95% CI 0.27%-0.95%; intersubgroup *P*=.14).

**Conclusions:**

The use of mobile app-based interventions yields a clinically significant HbA_1c_ reduction among adult outpatients with diabetes, especially among those with type 2 diabetes. Our study suggests that the clinical decision-making function needs further improvement and evaluation before being added to apps.

## Introduction

Diabetes mellitus poses enormous challenges to China’s health care system due to its mortality, prevalence, and costs. Of 8.3 million deaths in China in 2010, 37.3% (3.1 million) were attributable to cardiovascular disease, which was also one of the leading causes of disability-adjusted life-years [1]. Diabetes is not only an independent risk factor for cardiovascular disease [2], but also associated with increased mortality from a range of cardiovascular diseases (eg, ischemic heart disease and stroke), as well as noncardiovascular diseases (eg, infections) among Chinese adults [3]. In 2008, the estimated prevalence of diabetes was 9.7%, accounting for 92.4 million adults with diabetes [4]. A more recent cross-sectional survey reported an even larger estimate (11.6% among Chinese adults, ie, 113.9 million) in 2010 [5]. In addition, expenditures for the medical care of patients with diabetes were 3.38 times higher than for people with normal glucose tolerance [6].

Once diabetes is diagnosed, lifetime diabetes self-management is critical to glycemic control and is associated with the long-term prognosis for patients with diabetes. Diabetes self-management includes self-monitoring blood glucose, making healthy lifestyle choices (healthy eating, physical activity, tobacco cessation, weight management, and coping with stress), taking and managing medications, preventing diabetes complications (self-monitoring of foot health; active participation in screening for eye, foot, and renal complications; and immunizations), and setting self-selected behavioral goals [2]. In China, diabetes self-management education and support are provided during outpatient visits and are a huge burden on patients, their families, and the health system. Hence, a more cost-effective way to provide diabetes self-management education and support is essential for reducing the socioeconomic burden of diabetes.

Mobile apps are the computer programs or software installed on smart mobile devices, with computing and connectivity capability built right into an operating system. With the rapid and ongoing growth in wireless connectivity, more than 500 million Chinese were smartphone and apps users in 2016 [7]. In addition to their universality, apps provide real-time interactions and data transmission, which can be used in providing diabetes self-management education and support [8-10]. Accordingly, the American Diabetes Association (ADA) guideline has stated that mobile apps may be a useful element of effective lifestyle modification to prevent diabetes [2].

In the iTunes App Store for iOS and Google Play for Android, diabetes is one of the top-ranked categories [11,12], with more than 1100 different apps available for download. In contrast, according to a recent systemic review [13], there were only 5 randomized controlled trials (RCTs) assessing the effectiveness of apps in diabetes self-management. The contrast between the number of commercially available apps and the number of RCTs of apps demonstrates a shocking lack of evidence to support the recommendation of a specific app for diabetes self-management. Consequently, it is extremely difficult for clinicians and patients to choose a safe and effective one among the thousands of available apps [14].

Despite their variety and complexity, apps for diabetes self-management always share a limited number of basic functions, which can be classified into several simple categories (eg, self-monitoring, education, alerts and reminders, and communication) [15]. Therefore, indirect evidence from systematic reviews of existing RCTs can give insight into the efficacy of each app function, which is helpful in estimating the effectiveness of a specific app and making recommendations for effective functions. Nevertheless, prior systematic reviews involving mobile app-based interventions with multiple functions have not attempted to investigate their differential effectiveness [16-18]. As a result, it remains unclear how their functions contribute to the efficacy of apps.

To address functional efficacy, a classification of app functions is required [19]. Moreover, the classification should be comprehensive, with not only considerations of functions but also recommendations for clinical practice [15], as well as risk assessment [20,21]. However, existing classifications are inconsistent, and they primarily focus on functions [16,17,20,22-30]. Inconsistency and incompleteness have limited their use in classifying functions of diabetes self-management apps.

The aims of this systematic review of RCTs were to (1) develop and validate a taxonomy of apps for diabetes self-management, (2) perform a meta-analysis investigating the effects of mobile app-based interventions on glycemic control in adults with diabetes, and (3) explore the contribution of different functions to the glycemic efficacy of entire app-based interventions using the taxonomy and subgroup analyses.

## Methods

### Taxonomy Development and Validation

We developed a preliminary taxonomy based on previous classifications, evidence-based guidelines, and authoritative recommendations, and validated it by reviewing commercially available apps for diabetes management. The contents of the taxonomy were confirmed if all functions of the available apps could be classified. After validation, we proposed a final taxonomy for diabetes management apps. Multimedia Appendix 1, part A, shows the flow chart of taxonomy development. Multimedia Appendix 1, part B, shows the review of previous classifications [16,17,20,22-30].

The preliminary taxonomy was validated by a review of commercially available diabetes apps, as shown in Multimedia Appendix 1, part D. We searched the iTunes App Store (Apple Inc, Cupertino, CA, USA) and Google Play (Google Inc, Mountain View, CA, USA) (for the United States and China, February 1, 2016) using the terms “diabetes” OR “blood glucose” to identify apps for diabetes management. Apps with real-time interactions and any functions supporting self-monitoring of blood glucose were included. We excluded apps that were duplicated or were designed for health care providers. Apps that did not have English or Chinese versions and that had not been updated for at least 5 years were also excluded.

### Data Sources and Searches

We searched MEDLINE, EMBASE, the Cochrane Central Register of Controlled Trials (CENTRAL), and the Chinese Biomedical Literature Database using the terms “diabetes mellitus,” “blood glucose,” “blood glucose self-monitoring,” “mobile applications,” and “cell phones” from January 1, 2007, to May 30, 2016. We also searched for ongoing studies via ClinicalTrials.gov and checked the reference lists of relevant reviews and trials. Multimedia Appendix 2 lists the search strategy for MEDLINE. Necessary adjustments were made for searching other databases.

### Eligibility Criteria

We selected RCTs that compared mobile app-based interventions with standard care (free of app-based interventions) in adult outpatients with diabetes. Mobile app-based interventions were those that could provide real-time interactions with users through apps running on smart mobile devices.

Our primary outcome was the change in hemoglobin A_1c_ (HbA_1c_) concentration (%) from baseline. Our secondary outcomes were severe hypoglycemia (defined as the need for assistance from another person or very low glucose concentrations; this was study specific, eg, <2 mmol/L) and any other adverse events. We did further quantitative meta-analyses of primary and secondary outcomes if relevant data were available.

We excluded studies without any available data on HbA_1c_. We also excluded studies if their participants were children, adolescents, or pregnant women who required different therapeutic strategies for a more challenging or strict glycemic control [2]. Studies of apps for continuous glucose monitoring or continuous subcutaneous insulin infusion were excluded due to their medical devices nature. We excluded interventions without real-time interactions (eg, frequent interactions or passive interactions).

Two reviewers (YW and YD) independently screened titles and abstracts and then full texts to select eligible studies. Reviewers resolved disagreements through discussion or, if necessary, through discussion with an arbitrator (SL).

### Data Extraction and Quality Assessment

For each trial, 2 reviewers (YW and YD) independently extracted data using a structured abstraction form and classified functions according to our taxonomy. Then, 2 reviewers (YW and YD) independently used the Cochrane Collaboration’s tool to assess the risk of bias of included studies [31]. The Grading of Recommendations Assessment, Development and Evaluation (GRADE) approach was used to assess the quality of evidence for primary and secondary outcomes [32]. Reviewers resolved discrepancies by discussion or, if required, through adjudication by a third reviewer (SL).

### Data Synthesis and Analysis

We used a random-effects meta-analysis to pool the overall mean difference (MD) of the HbA_1c_ changes and the risk ratios of adverse events due to the possible clinical heterogeneity of each included study. For trials with unreported change-from-baseline standard deviations, we imputed by standard deviations at the baseline and at the end of the intervention using the formula *SD*_change_=√ *SD*^2^_baseline_+ *SD*^2^_final_–(2× *Corr* × *SD*_baseline_× *SD*_final_). The correlation coefficient (Corr) was calculated with the reported change-from-baseline standard deviations using the formula *Corr*=(*SD*^2^_baseline_+ *SD*^2^_final_– *SD*^2^_change_)/(2× *SD*_baseline_× *SD*_final_) [31]. Publication bias was examined in Begg funnel plots and with the Egger linear regression test [33,34]. We assessed the consistency of the results across the studies by the statistical heterogeneity with the *I*^2^ statistic [35]. The effect of the presence and absence of each function was examined in an exploratory subgroup analysis. We also conducted subgroup analyses of interventions that applied distinct technologies and had different risk levels. Statistical analyses were performed using RevMan version 5.3.0 (the Cochrane Collaboration) and STATA version 9.0 (StataCorp LLC). GraphPad Prism version 7.0 (GraphPad Software, Inc) was used to generate the figures included in this study.

## Results

### Taxonomy of Apps for Diabetes Self-Management

We designed a preliminary taxonomy with a functional axis, a clinical axis, and a risk axis as shown in Multimedia Appendix 1, part C. The functional axis consisted of 5 technical modules (ie, log, structured display, general education, personalized feedback, and communication) whose descriptive details were refined by previous classifications. The clinical axis consisted of 5 diabetes management modules (ie, monitoring, medication management, lifestyle modification, complication prevention, and psychosocial care) referring to the ADA guideline [2]. Functions were specified by crossing the functional axis (module) and the clinical axis (module), where we made sure that each function belonged to a functional or clinical classification, or both.

We developed the risk axis based on the US Food and Drug Administration (FDA) risk-based recommendation [36]. This recommendation classifies functionalities of mobile health technology into 3 categories: administrative (eg, general-purpose communication and population health management), which pose limited or no risk to patient safety; health management (eg, some clinical decision support and medication management), which pose potential but generally low risks; and medical device (eg, medical device accessories and medical device clinical decision support software), which present a relatively higher risk to patient safety. We assessed the risks of functions as low, potential, and high, accordingly.

During validation, we identified 1559 apps by searching the iTunes App Store and Google Play and excluded 1414 apps that were duplicated, were not for diabetes-management, were without English or Chinese versions, and had not been updated for at least 5 years. The remaining 145 eligible apps were downloaded onto smart mobile devices. After excluding those without real-time interactions and designed solely for health care providers, we included 96 apps and classified them by the preliminary taxonomy. As we could well classify all functions among the included apps by the taxonomy, and we identified all modules in the taxonomy in the included apps, we proposed the final taxonomy after this validation (Table 1).

**Table 1:** Taxonomy of apps for diabetes self-management.

Functional modules	Diabetes management modules				
	Monitoring^b^	Medication management^c^	Lifestyle modification	Complication prevention	Psychosocial care
Log^b^	⊕⊖⊖Recording self-monitoring parameters^d^; ⊕⊕⊖Recording other medical parameters^e^	⊕⊕⊖Recording used medications and side effects	⊕⊖⊖Recording activities, diets, and weight^f^	⊕⊖⊖Recording complication-related status^g^; ⊕⊖⊖Recording appointments with doctors	⊕⊖⊖Recording mood
Structured display	⊕⊖⊖Displaying data in a structured way				
General education	⊕⊖⊖Instructions for monitoring; ⊕⊕⊖Interpreting the parameters	⊕⊕⊖Diabetes process and treatment options; ⊕⊕⊖Using medications safely and effectively	⊕⊖⊖Incorporating nutritional management and physical activity into lifestyle	⊕⊕⊖Preventing, detecting, and handling acute complications and chronic complications^h^	⊕⊖⊖Addressing psychosocial issues and promoting behavior change
Personalized feedback	⊕⊖⊖Reminding to monitor; ⊕⊖⊖Off-target alert; ⊕⊕⊖Setting targets	⊕⊕⊖Reminding to take medications; ⊕⊕⊕Clinical decision making^i^	⊕⊖⊖Reminding to eat healthily and be active; ⊕⊕⊖Self-management decision making^j^	⊕⊖⊖Reminding to quit smoking, visit doctors, and prevent acute complications	N/A^k^
Communication	⊕⊖⊖General communication, connecting users with their peers and families through social networking, chat forums, or websites; ⊕⊕⊖Patient-clinician communication, in-app access to health care providers for medical support or consultation.				

^a^Risk assessment of a function: low risk (⊕⊖⊖), potential risk (⊕⊕⊖), and high risk (⊕⊕⊕). The overall risk assessment of an app was determined by the highest risk of included functions.

^b^Monitoring and log are basic modules.

^c^Medications for diabetes include insulin, oral antidiabetic agents, aspirin, antihypertensives, lipid-lowering medications, and vaccines.

^d^Self-monitoring parameters include blood glucose, blood pressure, heart rate, and pulse.

^e^Other medical parameters include cholesterol levels, hemoglobin A_1c_, urine test, and ketones.

^f^Activities include steps, duration, heart rate, and consumed calories; diets include food, water, nutritional values, carbohydrate counting, and calorie calculator; weight includes body mass index, body fat, and circumference.

^g^Complication-related status includes smoking, drinking, snoring, feet, eyes, teeth, and sensory status.

^h^Acute complications include hypoglycemia and hyperglycemia; chronic complications include cardiovascular disease and microvascular complications (ie, nephropathy, retinopathy, neuropathy).

^i^Clinical decision making is recommending treatment (eg, oral agents and insulin) by algorithms alone without the participation of health care providers.

^j^Self-management decision making is decision making on lifestyle modification by algorithms.

^k^N/A: not applicable.

### Characteristics and Classifications of Included Trials

**Figure 1:**
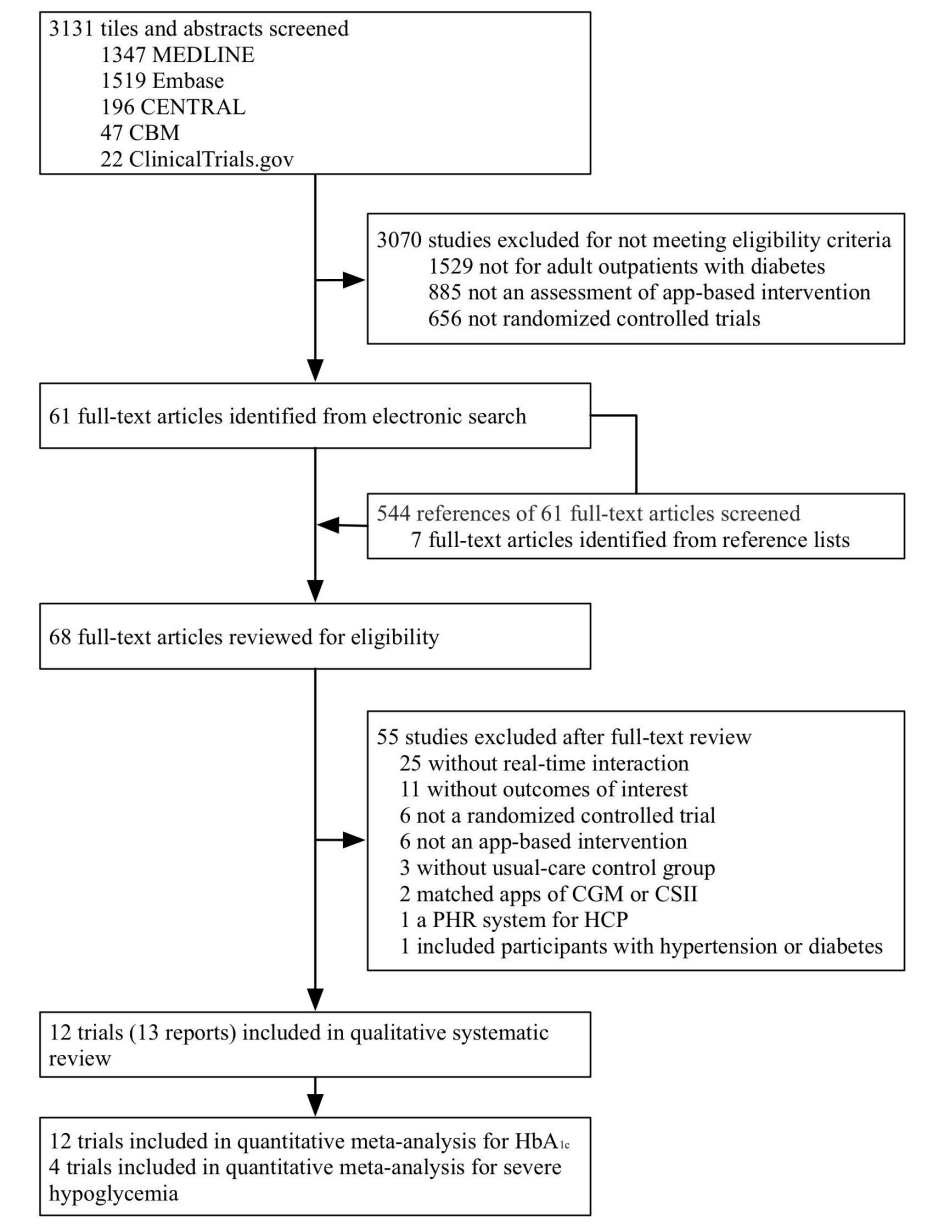
Study selection. CBM: Chinese Biomedical Literature Database; CENTRAL: Cochrane Central Register of Controlled Trials; CGM: continuous glucose monitoring; CSII: continuous subcutaneous insulin infusion; HbA_1c_: hemoglobin A_1c_; HCP: health care provider; PHR: personal health record.

We identified 3131 references using our search strategies and identified 544 references by checking the reference lists of relevant articles, 68 of which underwent a full-text review. This process excluded 55 studies, with the reasons listed in Multimedia Appendix 3. We included 12 trials from 13 references in a qualitative systematic review, evaluating 12 independent app-based interventions involving 974 outpatients with diabetes. Figure 1 shows the flow of study selection.

Across the 13 included references, the HbA_1c_ was obtained from 12 trials with 974 participants after a median follow-up period of 6 (range 3-12) months, and severe hypoglycemia was extracted from 4 trials of 346 participants after a median follow-up of 6 months. There were 5 trials that enrolled patients with type 1 diabetes mellitus (T1DM), 5 with type 2 diabetes mellitus (T2DM), and 2 with both types of diabetes.

Of the 12 included mobile app-based interventions, 1 is available in the iTunes Store and Google Play at the time of our study [37]. After taxonomic classification, all 12 included interventions had monitoring as a diabetes management module, followed by lifestyle modification (11/12, 92%), medication management (8/12, 67%), and complication prevention (2/12, 17%). Psychosocial care was not distinguished in any of the included interventions. For functional modules, all 12 interventions had a log as a basic functional module, followed by communication (9/12, 75%), a structured display (8/12, 67%), personalized feedback (8/12, 67%), and general education (6/12, 50%). To be noted, the included interventions only had patient-clinician communication instead of general communication.

Various technologies were applied for data transmission between users and mobile devices. Across the 12 included trials, 6 (50%) used wireless transmission through Wi-Fi, Bluetooth, near-field communication, or public switched telephone network, 5 (42%) used manual entry, and 1 (8%) used wire transmission through a data port connection.

Of the 12 included app-based interventions, we determined 3 (25%) to be of high risk due to having a clinical decision-making function. The definition of the clinical decision-making function was recommending treatment (eg, oral agents and insulin) by algorithms alone without the participation of health care providers. We determined that the other 9 interventions (75%) carried potential risk. Table 2 summarizes the modules, risks, and technologies of the mobile app-based interventions included in the meta-analysis [37-49].

**Table 2:** Characteristics, modules, risk assessments, and technologies of the included mobile app-based interventions.

Study	Country	No. patients: baseline/ end	Diabetes type	Follow-up (months)	Mean (SD) HbA_1c_^a^, %: baseline; end; change	Intervention	FM^b^	DMM^c^	Risk assessment^d^	Technology
Hsu, 2016 [38]	US	I^e^: 20/15; C^f^: 20/16	2	3	I: 10.8 (1.0); 7.7 (1.6); –3.2 (1.5) C: 10.9 (0.9); 8.9 (2.2); –2.0 (2.0)	Cloud-based diabetes management program	L, StD, GE, Co	M, MM, LM, CP	Potential	Wireless
Baron, 2017 [39]	UK	I: 45/40; C: 36/31	Both	9	I: 9.1 (1.8); 8.6 (1.6); C: 8.9 (1.7); 8.9 (1.6)	Mobile telehealth	L, StD, GE, PF, Co	M, MM, LM	Potential	Wireless
Drion, 2015 [40]	Netherlands	I: 31/30; C: 32/32	1	3	I: 7.73 (NR^g^); 7.91 (NR); C: 7.82 (NR); 7.91 (NR)	Diabetes Under Control (DBEES)	L, StD	M, MM, LM	Potential	Manual entry
Holmen, 2014 [41]; Torbjornsen, 2014 [42]	Norway	I: 51/39; C: 50/41	2	12	I: 8.1 (1.1); 7.8 (0.9); C: 8.3 (1.2); 8.2 (1.1)	Few Touch Application (FTA)	L, StD, GE, PF, Co	M, LM	Potential	Wireless
Waki, 2014 [43]	Japan	I: 27/24; C: 27/25	2	3	I: 7.1 (1.0); 6.7 (0.7); C: 7.0 (0.9); 7.1 (1.1)	DialBetics	L, StD, GE, PF, Co	M, LM	Potential	Wireless
Kirwan, 2013 [37]	Australia	I: 36/28; C: 36/32	1	9	I: 9.1 (1.2); 8.0 (0.7); C: 8.5 (0.9); 8.4 (1.0)	Glucose Buddy	L, StD	M, MM, LM	Potential	Manual entry
Rossi, 2013 [44]	Italy	I: 63/55; C: 64/57	1	6	I: 8.4 (NR); 7.9 (NR); –0.5 (NR); C: 8.5 (NR); 8.1 (NR); –0.5 (NR)	Diabetes Interactive Diary	L, PF, Co	M, MM, LM	High	Manual entry
Charpentier, 2011 [45]	France	I: 60/56; C: 61/60	1	6	I: 9.2 (1.1); 8.6 (1.1); C: 8.9 (0.9); 9.1 (1.2)	Diabeo system	L, StD, PF, Co	M, MM, LM	High	Manual entry
Rossi, 2010 [46]	Italy	I: 67/58; C: 63/61	1	6	I: 8.2 (0.8); 7.8 (0.8); –0.4 (0.9); C: 8.4 (0.7); 7.9 (1.1); –0.5 (1.0)	Diabetes Interactive Diary	L, PF, Co	M, MM, LM	High	Manual entry
Yoo, 2009 [47]	Korea	I: 62/57; C: 61/54	2	3	I: 7.6 (0.9); 7.1 (0.8); C: 7.4 (0.9) 7.6 (1.0)	Ubiquitous Chronic Disease Care (UCDC) system	L, GE, PF	M, LM	Potential	Wire
Istepanian, 2009 [48]	UK	I: 72/NR; C: 65/NR	Both	9	I: 7.9 (1.5); 7.8 (NR); C: 8.1 (1.6) 8.4 (NR)	Mobile phone telemonitoring system	L, Co	M	Potential	Wireless
Quinn, 2008 [49]	US	I: 15/13; C: 15/13	2	3	I: 9.5 (NR); 7.5 (NR); C: 9.1 (NR); 8.4 (NR)	WellDoc Communications	L, StD, GE, PF, Co	M, MM, LM, CP	Potential	Wireless

^a^HbA_1c_: hemoglobin A_1c_.

^b^FM: functional modules are communication (Co), general education (GE), log (L), personalized feedback (PF), and structured display (StD).

^c^DMM: diabetes management modules are complication prevention (CP), lifestyle modification (LM), monitoring (M), and medication management (MM).

^d^The overall risk assessment of an intervention was determined by the highest risk of its functions.

^e^I: intervention group.

^f^C: control group.

^g^NR: not reported.

### Risks of Bias of Included Trials

**Figure 2:**
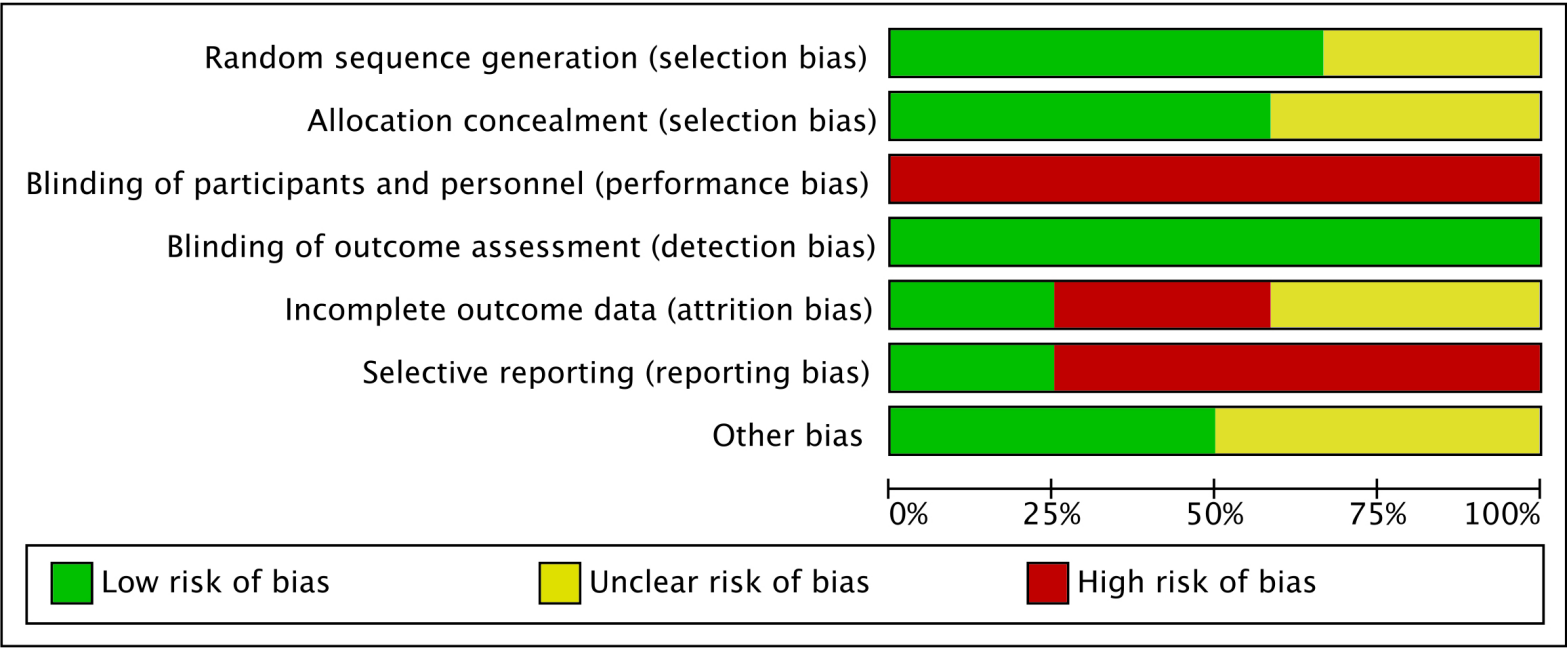
Risk of bias for the primary outcome (hemoglobin A_1c_ changes): review authors’ judgments about each risk-of-bias item presented as percentages across all included studies.

**Figure 3:**
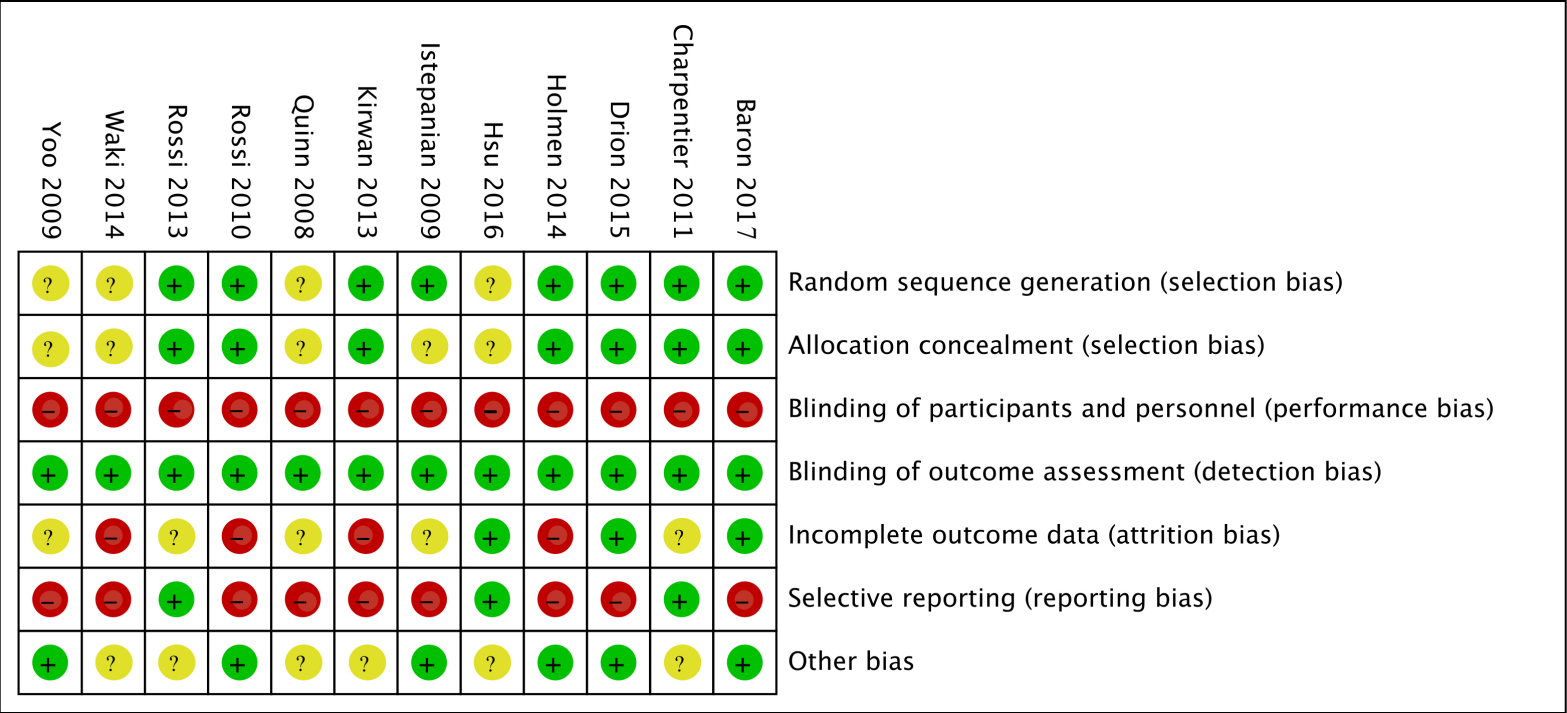
Risk-of-bias summary for the primary outcome (hemoglobin A_1c_ changes): review authors’ judgments about each risk-of-bias item for each included study.

Only 67% (8/12) of the trials adequately reported allocation sequence generation, and 58% (7/12) adequately reported concealing the allocation sequence. As an objective outcome, all trials adequately blinded the assessment of the primary outcome (HbA_1c_ changes). The corresponding proportion for incomplete outcome data was 25% (3/12), for selective reporting was 25% (3/12), and for other sources of bias was 50% (6/12). Figure 2 and Figure 3 present the risk-of-bias assessments of the primary outcome (HbA_1c_ changes) for each domain of each included study. Multimedia Appendix 4 lists the detailed characteristics, taxonomic classification, and risk of bias of each included trial.

### Effects of Mobile App-Based Interventions on HbA1c

**Figure 4:**
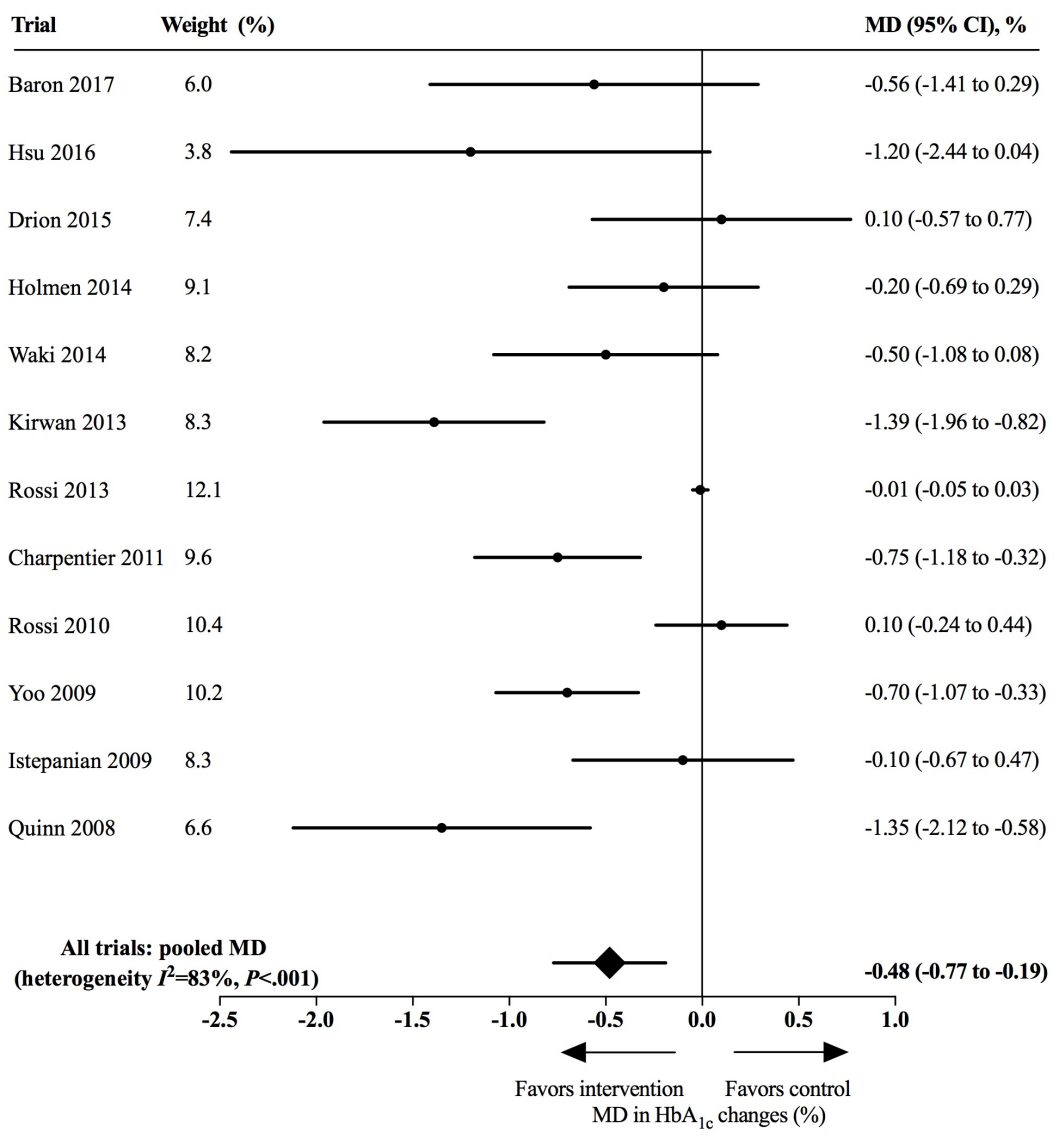
Effects of app-based mobile health interventions on hemoglobin A_1c_ (HbA_1c_). MD: mean difference.

The use of mobile app-based interventions was associated with a clinically significant HbA_1c_ reduction of 0.48% (95% CI 0.19%-0.78%, *I*^2^=76%, *P*<.001) compared with standard care alone, as Figure 4 shows. However, the funnel plot was found to be asymmetrical (Multimedia Appendix 5), with Egger test indicating a potential publication bias (*P*=.008). Overall, we used the GRADE approach to rate the quality of the evidence for HbA_1c_ as low due to the potential publication bias and study limitations (lack of allocation concealment, lack of blinding of participants and personnel, incomplete outcome data, selective reporting, and other biases as shown in Figure 2 and Figure 3; Multimedia Appendix 6).

**Figure 5:**
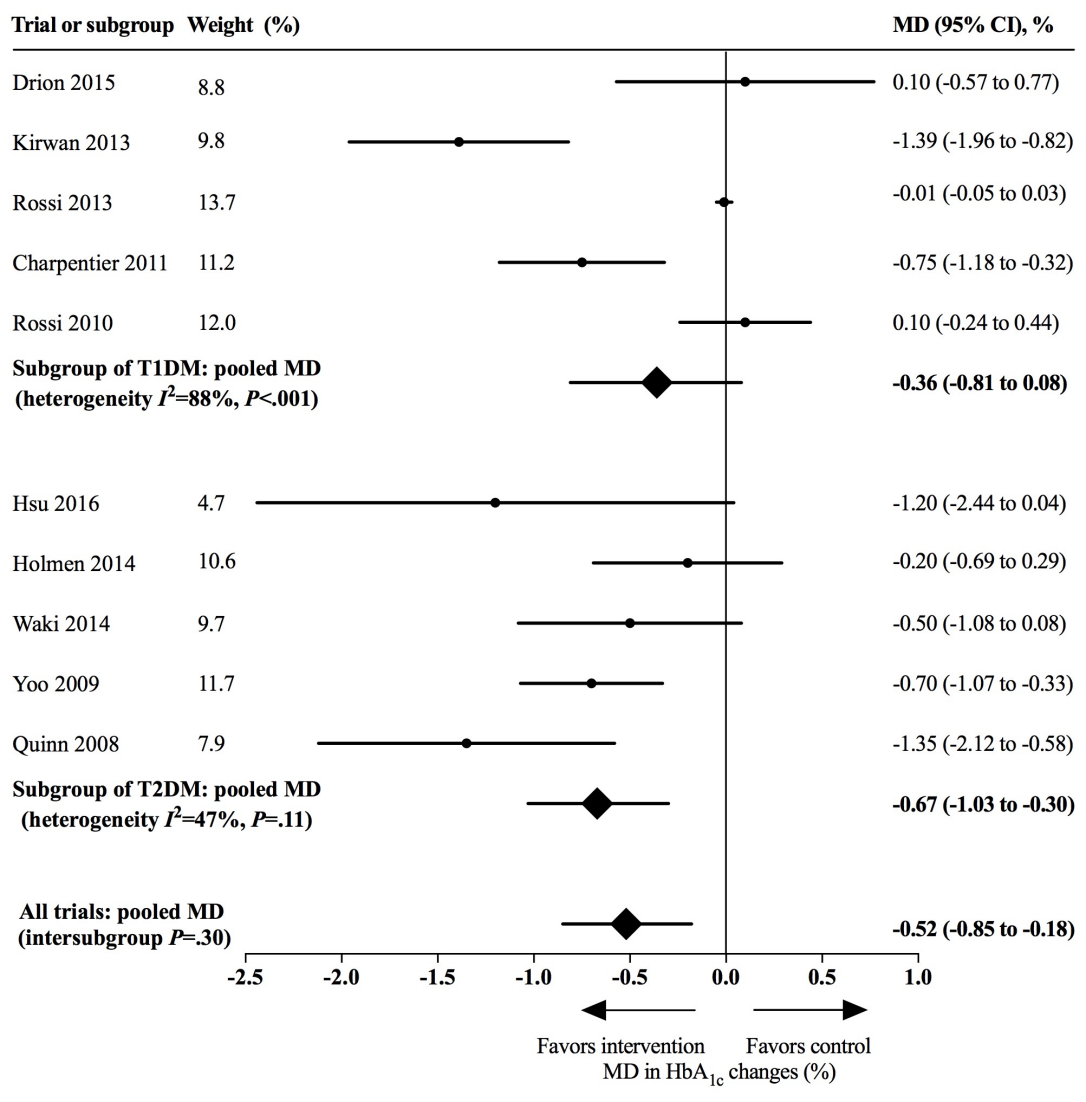
Effects of app-based mobile health interventions on hemoglobin A_1c_ (HbA_1c_) for patients with type 1 diabetes (T1DM) and type 2 diabetes (T2DM). MD: mean difference.

We performed a post hoc exploratory analysis for 5 trials enrolling patients with T1DM and 5 trials enrolling patients with T2DM. The use of app-based interventions did not achieve statistical significance among patients with T1DM (MD 0.37%, 95% CI –0.12%-0.86%, *I*^2^=86%, *P*<.001). Larger HbA_1c_ reductions were noted for patients with T2DM (MD 0.67%, 95% CI 0.30%-1.03%, *I*^2^=47%, *P*=.11). The intersubgroup difference was not significant (*P*=.30) (Figure 5).

### Effects of Modules, Risks, and Technologies of App-Based Interventions on HbA1c

We noted a greater HbA_1c_ reduction when interventions included a complication prevention module (with complication prevention: MD 1.31%, 95% CI 0.66%-1.96%, *I*^2^=0%, *P*=.84 vs without: MD 0.38%, 95% CI 0.09%-0.68%, *I*^2^=76%, *P*<.001; test for subgroup difference *P*=.01). Having a structured display was also associated with a larger HbA_1c_ reduction (with structured display: MD 0.69%, 95% CI 0.32%-1.06%, *I*^2^=63%, *P*=.008 vs without: MD 0.17%, 95% CI –0.18% to 0.53%, *I*^2^=75%, *P*=.007; test for subgroup difference *P*=.05).

For high-risk interventions with a clinical decision-making function, the reduction of HbA_1c_ was 0.19% (95% CI –0.24%-0.63%, *I*^2^=82%, *P*=.004), while the reduction was 0.61% (95% CI 0.27%-0.95%, *I*^2^=64%, *P*=.005) for potential-risk interventions without clinical decision making (test for subgroup difference *P*=.104.

**Figure 6:**
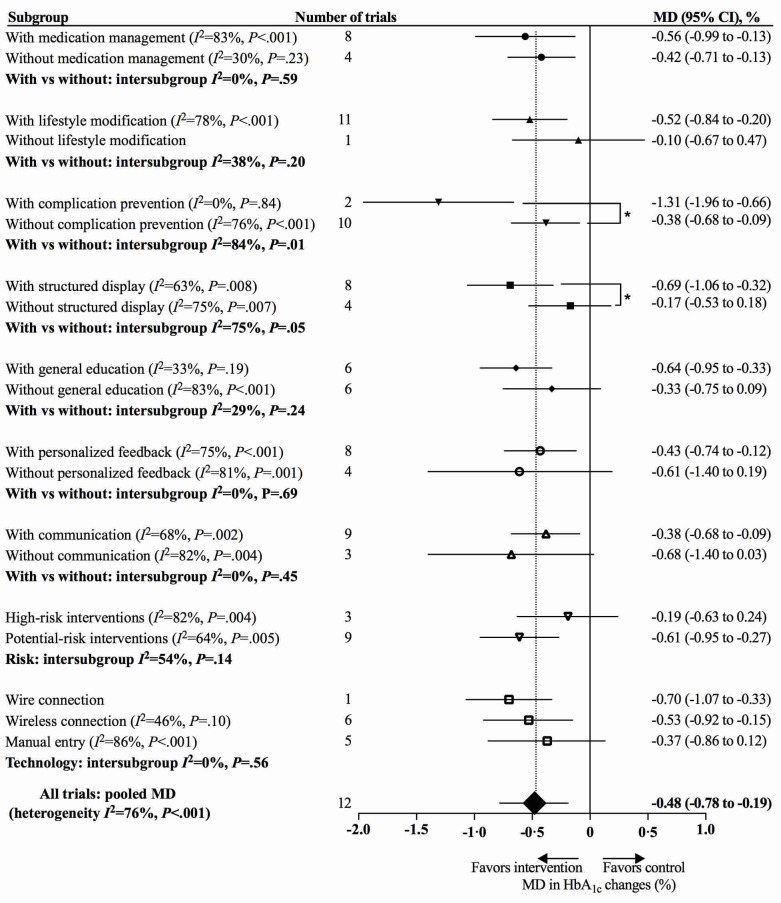
Effects of modules, risks, and technologies of app-based mobile health interventions on hemoglobin A_1c_ (HbA_1c_). MD: mean difference.

Interventions using manual entry showed an associated lower HbA_1c_ reduction without statistical significance (wire connection: MD 0.70%, 95% CI 0.33%-1.07% vs wireless connection: MD 0.53% CI 0.15%-0.92%, *I*^2^=46%, *P*=.10 vs manual entry: MD 0.37%, 95% CI –0.12%-0.86%, *I*^2^=86%, *P*<.001; test for subgroup difference *P*=.56) (Figure 6).

### Adverse Events of Included Trials

Adverse events were reported variably among the 5 included studies [38,41,44-46]. One study reported no adverse clinical event but several undesired technical events in the automatic data transmission between the glucometer and the app [41]. A total of 4 studies reported the participants or the proportion of participants with, or the incidence of severe hypoglycemia and overall hypoglycemia [38,44-46]. None of the studies reported any other kinds of adverse events or death.

For severe hypoglycemia, 1 study reported significantly fewer episodes in the intervention group (0.33 vs 2.29 events/patient-year) [44]; 3 studies reported no severe hypoglycemia in either the intervention or control group [38,41,46]. Of the 5 studies, 4 reported that 3 participants in the intervention group and 3 in the standard-care group had severe hypoglycemia episodes, with a pooled risk ratio of 1.07 (95% CI 0.23%-5.09%) [38,41,45,46]. The pooled risk ratio was 1.62 (95% CI 0.48%-5.40%) for the 3 trials reporting overall hypoglycemia [38,41,46] (Multimedia Appendix 7).

Overall, we rated the quality of the evidence for severe hypoglycemia as low due to imprecision (wide confidence intervals including null effect) and study limitations (risk of bias in 4 trials), and as very low for adverse events owing to inconsistency (substantial diversity in the definitions of outcome measures), imprecision (small sample sizes and low event rates), and study limitations (risk of bias in 5 trials) (Multimedia Appendix 5).

## Discussion

### Principal Findings

As most commercially available apps for diabetes self-management were not tested by RCTs, both the patients and the clinicians needed indirect evidence to guide their assessment while choosing apps. The purposes of this review were to investigate the glycemic efficacy of mobile app-based interventions, and to explore the differential effectiveness of their functions. We could not use existing classifications for the functions of the app-based interventions because of inconsistency and incompleteness. As a result, we developed and validated a comprehensive taxonomy for the functions of diabetes self-management apps. To our knowledge, this is the first comprehensive taxonomy with clinical, functional, and risk axes, and this is the first review exploring the contribution of each function to the effectiveness of entire apps.

The meta-analysis of 12 RCTs demonstrated that app-based interventions were associated with a statistically and clinically significant HbA_1c_ reduction of 0.48% (95% CI 0.19%-0.78%). We noted larger HbA_1c_ reductions for patients with T2DM (MD 0.67%, 95% CI 0.30%-1.03%) than those with T1DM (MD 0.37%, 95% CI –0.12%-0.86%). The exploratory subgroup analyses showed that having a clinical decision-making function in app-based interventions was not associated with a greater HbA_1c_ reduction (with clinical decision making: MD 0.19%, 95% CI –0.24%-0.63% vs without: MD 0.61%, 95% CI 0.27%-0.95%; intersubgroup *P*=.14). There were no excess adverse events related to the included app-based interventions.

### Comparison With Prior Work

Consistent with previous reviews involving mobile app-based interventions [16-18], our study indicated that the use of mobile app-based interventions is associated with a clinically significant HbA_1c_ reduction in the diabetes management of adult outpatients. Our results suggested that glycemic control of adult outpatients with diabetes can benefit from apps. A subgroup analysis of diabetes types showed a larger HbA_1c_ reduction in patients with T2DM than in those with T1DM. This difference is consistent with a previous review [17] and may be explained, at least in part, by the complexity of the management of T1DM [2]. Patients with T1DM, especially those at a young age, require intensive management, which increases the burdens placed on the management of T1DM. Our result suggested that current apps may not be good enough to support the intensive management of T1DM.

Our study developed a 3-axis taxonomy for diabetes apps, with rows of functional modules, columns of diabetes management modules, and cells of functions with risk assessments. The functional, clinical, and risk axes were developed based on previous classifications, the ADA’s guidelines, and the FDA’s risk recommendation, respectively. The 3-axis design of the taxonomy is comprehensive and decreases the possibility of misclassification. Additionally, this 3-axis design is applicable for diseases other than diabetes by adjusting the modules in the clinical axis. The validation process guarantees that our taxonomy can be used to classify commercial diabetes apps. Differences in the detected effect sizes in subsequent subgroup analyses indicated the utility of our taxonomy.

Our taxonomy has some advantages. First, it is a comprehensive taxonomy with functional, clinical, and risk axes. The taxonomy permits subsequent exploratory subgroup analyses of multifunction apps, which give insights into the efficacy and risk of each module in diabetes apps. Comparatively, existing classifications appear to be incomplete or inconsistent. Previous classifications have mainly focused on the functions of apps, which, as a result, have made them applicable only for functional evaluation [16,17,22-25,27-30]. Some similar functions in these classifications have diverse definitions and descriptive details. Moreover, some functions lack clinical considerations, such as education, feedback, and decision support [16,17,23-25,27-30]. Only 1 classification addresses risk assessment [20]. These classifications, on the one hand, demonstrate a requirement to classify apps comprehensively, and on the other hand, they indicate the limitation of each independent classification.

Second, our taxonomy can be of some help in the development and evolution of diabetes apps. App developers are usually technicians without a clinical background. As a result, the evidence-based guidelines for diabetes management are easily ignored during app development. For example, we found that complication prevention and psychosocial care were uncommon in the app-based interventions we examined. However, complication prevention behaviors and emotional well-being are associated with positive diabetes outcomes according to the guidelines [2]. Previous reviews also suggested that diabetes apps lacked essential modules and neglected evidence-based guidelines [15,50]. With a clinical axis of diabetes management modules developed based on guidelines, our taxonomy makes it straightforward for app developers to follow evidence-based guidelines during the design and development of diabetes apps.

Third, our taxonomy permits subsequent exploratory subgroup analyses of multifunction apps, which give insights into the efficacy and risk of each module in diabetes apps.

Our exploratory subgroup analyses suggested a limited efficacy of clinical decision making, which was defined as recommending treatment (eg, oral agents and insulin) by algorithms alone without the participation of health care providers and was determined to be high risk according to our taxonomy. Traditionally, clinical decisions are made during a face-to-face interview after a complete assessment. Built-in clinical decision support systems, however, are less likely to collect data and assess status as thoroughly as face-to-face consultations do. Without adequate data and well-designed algorithms, clinical decision-making functions can make inappropriate decisions and pose risks to patients [51,52]. Additionally, complex data collection may cause technical difficulties. Despite the above-mentioned issues, clinical decision making can be found in diabetes apps both in trials [44-46] and in app stores [23,28]. Therefore, we suggest that app developers should employ caution to add clinical decision making into diabetes apps, and patients should consult with health care providers on using apps for diabetes self-management.

Our subgroup analyses indicated that having a complication prevention module in the apps was associated with a greater HbA_1c_ reduction. Complication prevention behaviors such as smoking cessation and hypoglycemia prevention are critical components of diabetes management according to current guidelines [2]. However, only 2 included app-based interventions had a complication prevention module. Further studies are needed to confirm the efficacy of a complication prevention module. Meanwhile, having a structured display module was associated with a larger HbA_1c_ reduction. The structured display module may improve blood glucose self-monitoring behaviors by displaying structured self-monitoring of blood glucose profiles. Having a structured display is consistent with current clinical guidelines, in which self-monitoring of blood glucose is a critical element in the management of diabetes [2].

Having a lifestyle modification in app-based interventions was associated with a trend toward reduced HbA_1c_, as was having a general education module. The modules of lifestyle modification and general education may raise awareness of lifestyle change and self-management. Since these 2 modules pose limited risks to patients with diabetes, it might be reasonable to add lifestyle modification and general education to diabetes apps.

The data suggested limited glycemic efficacy of having a personalized feedback module. However, considerable uncertainty and limitations exist regarding its efficacy. Given that the personalized feedback module has a relatively high risk, further evaluation is required before adding a personalized feedback module to diabetes apps. Consistent with a previous review [15], our review found that none of the interventions included a general communication function. Particular attention should be paid to the complexity and variety of the patient-clinician communication function as shown in Multimedia Appendix 8. As for the technologies, direct data transmission between users and mobile devices using wire or wireless connections was associated with a trend toward reduced HbA_1c_, which could be explained by the convenience and accuracy of the technology.

### Limitations

Our study also has some limitations. First, the exploratory and observational nature of our subgroup analyses and the possibility of misclassification prevented us from drawing a solid conclusion about the modular efficacies and risks. Second, we examined only 12 trials in our study, which may limit the strength of this systematic review. Third, we noted the asymmetry of the funnel plot, which indicated a potential risk of publication bias in our systematic review.

### Conclusions

In our study, we developed a 3-axis taxonomy for diabetes self-management apps. Mobile app-based interventions improve glycemic control in adult outpatients with diabetes, especially in those with T2DM. Our analyses suggest that clinical decision making requires further improvement and evaluation before being added to apps. Safety issues such as hypoglycemia and other adverse events are being overlooked and need attention in future investigations.

## Appendix

Multimedia Appendix 1 Taxonomy development and validation.

Multimedia Appendix 2 MEDLINE search strategy.

Multimedia Appendix 3 List of excluded references with reasons for exclusions.

Multimedia Appendix 4 The detailed information, taxonomic classification, and risk of bias of included trials.

Multimedia Appendix 5 Funnel plot.

Multimedia Appendix 6 GRADE for primary and secondary outcomes.

Multimedia Appendix 7 Meta-analyses for adverse events.

Multimedia Appendix 8 Classifications of the patient-clinician communication function.

## Abbreviations

ADA: American Diabetes Association
CENTRAL: Cochrane Central Register of Controlled Trials
FDA: Food and Drug Administration
GRADE: Grading of Recommendations Assessment, Development and Evaluation
HbA1c: hemoglobin A1c
MD: mean difference
RCT: randomized controlled trial
T1DM: type 1 diabetes mellitus
T2DM: type 2 diabetes mellitus
