# Correction: Mobile App-Based Interventions to Support Diabetes Self-Management: A Systematic Review of Randomized Controlled Trials to Identify Functions Associated with Glycemic Efficacy

**DOI:** 10.2196/mhealth.8789

**Published:** 2018-01-15

**Authors:** Yuan Wu, Xun Yao, Giacomo Vespasiani, Antonio Nicolucci, Yajie Dong, Joey Kwong, Ling Li, Xin Sun, Haoming Tian, Sheyu Li

**Affiliations:** ^1^ Department of Endocrinology and Metabolism West China Hospital Sichuan University Chengdu China; ^2^ Department of Academic Affairs West China School of Medicine Sichuan University Chengdu China; ^3^ Diabetes Unit Madonna del Soccorso Hospital San Benedetto del Tronto (AP) Italy; ^4^ Center for Outcomes Research and Clinical Epidemiology Pescara Italy; ^5^ Chinese Evidence-Based Medicine Center West China Hospital Sichuan University Chengdu China

In “Mobile App-Based Interventions to Support Diabetes Self-Management: A Systematic Review of Randomized Controlled Trials to Identify Functions Associated with Glycemic Efficacy” (JMIR Mhealth Uhealth 2017;5(3):e35), there was an error in Table 2. The “Mean (SD) HbA_1c_, %: baseline; end; change” for “Rossi 2013” should read “I: 8.4 (NR); 7.9 (NR); –0.5 (NR); C: 8.5 (NR); 8.1 (NR); –0.5 (NR)” instead of “I: 8.4 (0.1); 7.9 (0.1); –0.5 (0.1); C: 8.5 (0.1); 8.1 (0.1); –0.5 (0.1)”.

As a result, data were slightly changed as follows:

1. In the Results subsection of the Abstract, the data were changed in 4 places:
  - “Across 12 included trials involving 974 participants, using app-based interventions was associated with a clinically significant reduction of HbA_1c_ (MD 0.48%, 95% CI 0.19%-0.78%) without excess adverse events”;
  - “Larger HbA_1c_ reductions were noted among patients with type 2 diabetes than those with type 1 diabetes (MD 0.67%, 95% CI 0.30%-1.03% vs MD 0.37%, 95% CI –0.12%-0.86%)”;
  - “Having a complication prevention module in app-based interventions was associated with a greater HbA_1c_ reduction (with complication prevention: MD 1.31%, 95% CI 0.66%-1.96% vs without: MD 0.38%, 95% CI 0.09%-0.67%; intersubgroup *P*=.01), as was having a structured display (with structured display: MD 0.69%, 95% CI 0.32%-1.06% vs without: MD 0.17%, 95% CI –0.18%-0.53%; intersubgroup *P*=.05).”
  - “However, having a clinical decision-making function was not associated with a larger HbA_1c_ reduction (with clinical decision making: MD 0.19%, 95% CI –0.24%-0.63% vs without: MD 0.61%, 95% CI 0.27%-0.95%; intersubgroup *P*=.14).
2. In the Effects of Mobile App-Based Interventions on HbA_1c_ subsection of the Results, the data were changed in 4 places:
  - ”The use of mobile app-based interventions was associated with a clinically significant HbA_1c_ reduction of 0.48% (95% CI 0.19%-0.78%, *I*^2^=76%, *P*<.001);
  - “The use of app-based interventions did not achieve statistical significance among patients with T1DM (MD 0.37%, 95% CI –0.12%-0.86%, *I*^2^=86%, *P*<.001)”;
  - Figure 4;
  - Figure 5.
3. In the Effects of Modules, Risks, and Technologies of App-Based Interventions on HbA_1c_ subsection of the Results, data were corrected in the following 5 places:
  - “We noted a greater HbA_1c_ reduction when interventions included a complication prevention module (with complication prevention: MD 1.31%, 95% CI 0.66%-1.96%, *I*^2^=0%, *P*=.84 vs without: MD 0.38%, 95% CI 0.09%-0.68%, *I*^2^=76%, *P*<.001; test for subgroup difference *P*=.01)”;
  - “Having a structured display was also associated with a larger HbA_1c_ reduction (with structured display: MD 0.69%, 95% CI 0.32%-1.06%, *I*^2^=63%, *P*=.008 vs without: MD 0.17%, 95% CI –0.18% to 0.53%, *I*^2^=75%, *P*=.007; test for subgroup difference *P*=.05)”;
  - “For high-risk interventions with a clinical decision-making function, the reduction of HbA_1c_ was 0.19% (95% CI –0.24%-0.63%, *I*^2^=82%, *P*=.004), while the reduction was 0.61% (95% CI 0.27%-0.95%, *I*^2^=64%, *P*=.005) for potential-risk interventions without clinical decision making (test for subgroup difference *P*=.14)”;
  - “Interventions using manual entry showed an associated lower HbA_1c_ reduction without statistical significance (wire connection: MD 0.70%, 95% CI 0.33%-1.07% vs wireless connection: MD 0.53% CI 0.15%-0.92%, *I*^2^ =46%, *P*=.10 vs manual entry: MD 0.37%, 95% CI –0.12%-0.86%, *I*^2^ =86%, *P*<.001; test for subgroup difference *P*=.56)”;
  - Figure 6.
4. In the Principal Findings subsection of the Discussion, the data were corrected in 4 places:
  - 1) “The meta-analysis of 12 RCTs demonstrated that app-based interventions were associated with a statistically and clinically significant HbA_1c_ reduction of 0.48% (95% CI 0.19%-0.78%)”;
  - 2) “We noted larger HbA_1c_ reductions for patients with T2DM (MD 0.67%, 95% CI 0.30%-1.03%) than those with T1DM (MD 0.37%, 95% CI –0.12%-0.86%)”;
  - 3) “The exploratory subgroup analyses showed that having a clinical decision-making function in app-based interventions was not associated with a greater HbA_1c_ reduction (with clinical decision making: MD 0.19%, 95% CI –0.24%-0.63% vs without: MD 0.61%, 95% CI 0.27%-0.95%; intersubgroup *P*=.14)”.

The corrected article will appear in the online version of the paper on the JMIR website on January 15, 2018, together with the publication of this correction notice. Because this was made after submission to PubMed or Pubmed Central and other full-text repositories, the corrected article also has been re-submitted to those repositories.

Please see the corrected data and figures here.

**Figure 4:**
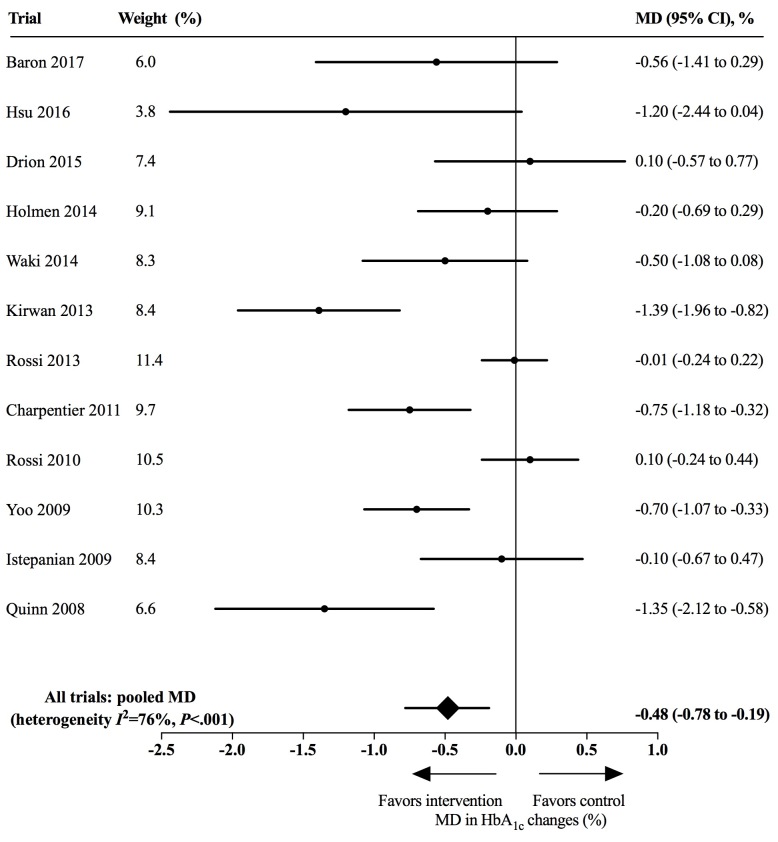
Effects of app-based mobile health interventions on hemoglobin A_1c_ (HbA_1c_). MD: mean difference.

**Figure 5:**
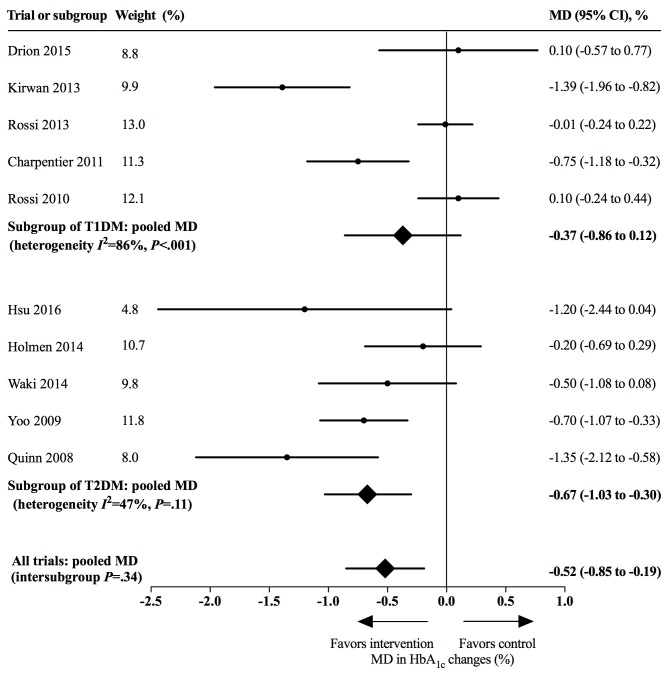
Effects of app-based mobile health interventions on hemoglobin A_1c_ (HbA_1c_) for patients with type 1 diabetes (T1DM) and type 2 diabetes (T2DM). MD: mean difference.

**Figure 6:**
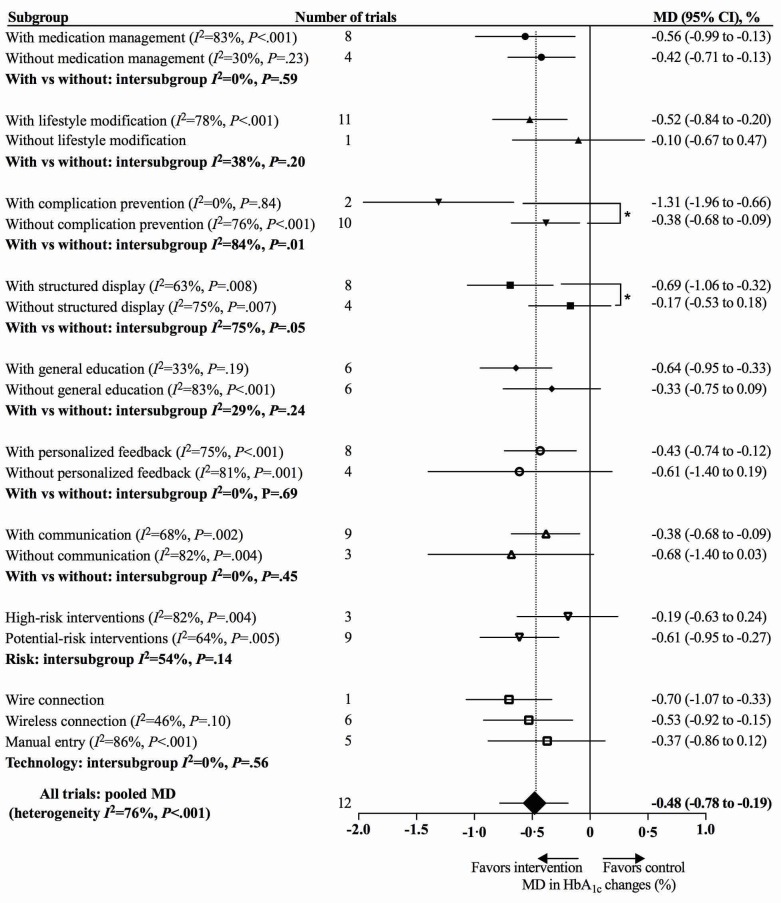
Effects of modules, risks, and technologies of app-based mobile health interventions on hemoglobin A_1c_ (HbA_1c_). MD: mean difference.

