# Supplementary material for: Mobile App-Based Interventions to Support Diabetes Self-Management: A Systematic Review of Randomized Controlled Trials to Identify Functions Associated with Glycemic Efficacy
Source: JMIR Mhealth Uhealth. 2017 Mar 14;5(3):e35. doi: 10.2196/mhealth.6522 (PMC5373677; doi:10.2196/mhealth.6522)
Supplement: Multimedia Appendix 1 [file mhealth_v5i3e35_app1.pdf]

Multimedia Appendix 1 Taxonomy development and validation

A Flow chart of taxonomy development

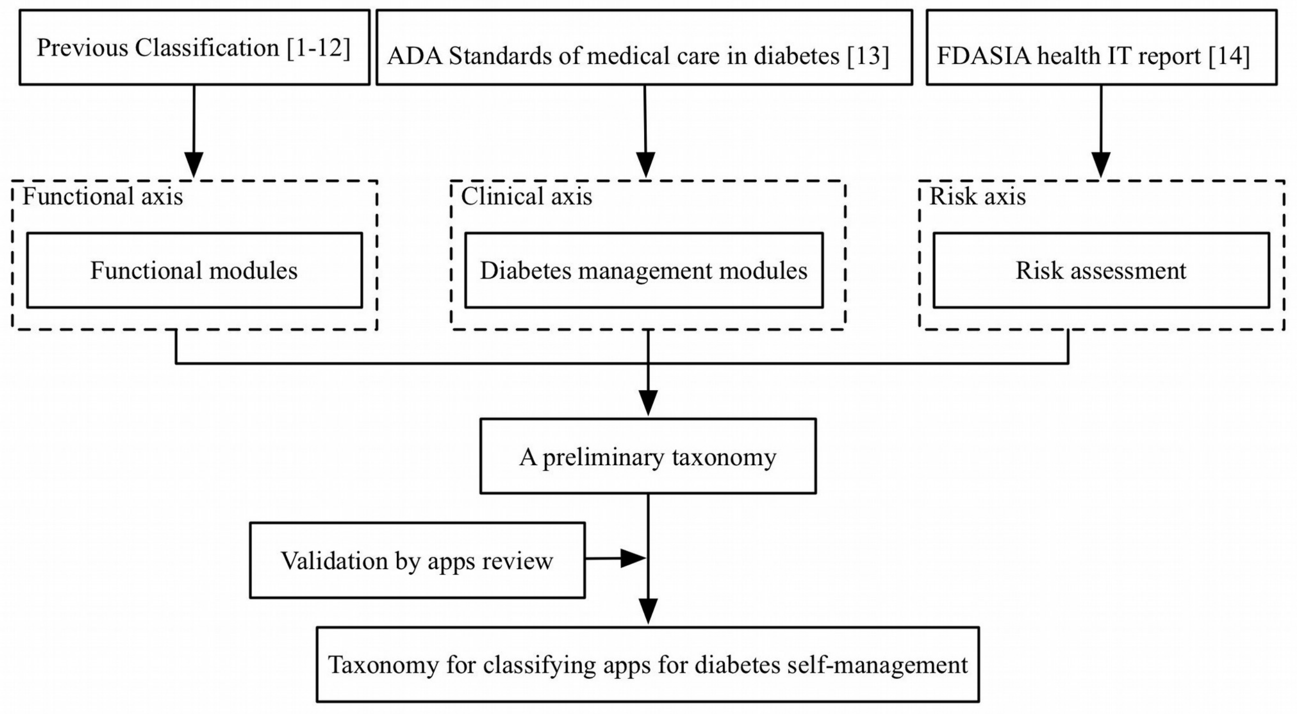

ADA, American Diabetes Associations. FDA, Food and Drug Administration.

B Review of previous classifications of mobile health technology for diabetes management

Author year	Review	Classification	Functionalities	Our judgements			
				Functional consideration	Clinical consideration	Risk assessment	Comments
De Ridder 2016 [1]	34 studies	Incentive-driven technologies (IDTs)	Education, reminder, feedback, social, alert, gasification, financial	Education, reminder, feedback, social, alert, gamification, financial	-	-	Without clinical consideration and risk assessment;<br>Broad definition of functionalities without descriptive details;
Basilico 2016 [2]	41 apps in Apple US store	Features	Blood glucose/medication/nutrition/activity log, data representation/delivery, insulin calculator, community support	log, data representation/delivery, insulin calculator, community support	Blood glucose, medication, nutrition, activity	-	Without risk assessment;
Riazi 2015 [3]	67 studies	Interventions	Telephone coaching, clinical decision support system, electronic medical record, distance learning, computerized insulin dose adjustment, personal health record, mobile health/short message service, telemedicine/telehealth summation	Telehealth coaching and education, CDSS, PHR	Insulin	-	Without risk assessment;<br>Without systematic clinical consideration in each functionality (ie, education);<br>Broad definition of functionalities without descriptive details (eg, short message service);<br>Duplicated functionalities;
Williams 2015 [4]	20 apps	Features and functionalities	Blood glucose recording/annotation, activity logs, data exportation/uploading, connect directly with a glucometer, reminder to take medications or check blood glucose	Recording and log, data transmission, connection, reminder	Blood glucose, activity, medication	-	Without risk assessment;
Lewis 2014	-	Functionalities	Low risk and pose minimal risk to patients if	Education, communication with	BMI, medication	Low risk, harm,	Broad definition without

[5]		and risks	misused: BMI calculator, patient education, accessing PHR, access guidelines, formulary;<br>Cause harm if used inappropriately or without adequate training: inter professional consultation and referral, drug conversion apps, entering treatment requests;<br>Significant risk to patients due to either inherent complexity functionality or potential for harm if misuse: diagnostic support apps, patient decision apps, medical calculators;<br>Apps pose significant risk to patients due to combination of inherent complexity, functionality and potential for major harm if misused: clinical decision support tools, control devices;	healthcare providers, CDSS, diagnose		significant risk, major harm	descriptive details of functionalities;<br>Without systematic clinical consideration in each functionality (ie, education, CDSS, diagnose);<br>Unclear boundary between different risk levels;<br>Unclear definition of misuse;<br>Not based on comprehensive apps or studies review;
El-Gayar 2013 [6]	71 apps and 16 studies	Functions	Blood glucose, medication, diet, physical exercise, education, weight, blood pressure, communication, PHR, decision support, data entry automation, security, social networking	Track, education, communication, PHR, decision support, data entry automation, security, social networking	Blood glucose, medication, diet, physical exercise, weight, blood pressure	-	Without risk assessment;<br>Without systematic clinical consideration in each functionality (ie, education, decision support);
El-Gayar 2013 [7]	104 studies	Technology and functionality	Internet: sending glucose level, medication and diet information; enquiring; accessing of personal electronic medical record, data visualising; education; feedback;<br>Phone: uploading the glucose level; messaging the clinicians; self-management decision support; automatic glucose level upload;<br>Decision support: real-time feedback; insulin and medication regimen; integrated with patient’s	Data transmission, data, visualising, automation, PHR, education, feedback, communication with clinicians, DSS on self-management, CDSS on regimen	Glucose, medication, diet	-	Without risk assessment;<br>Without systematic clinical consideration in each functionality (ie, education, feedback, DSS, CDSS);<br>Duplicated functionalities due to categorised by technology (ie, the internet, phone, and decision

			medical record;<br>Telemedicine: video conferencing with clinicians; uploading and transmitting data; receiving feedback				support);
Arsand 2012 [8]	10 apps	Functions	Automatic BG data transfer, educational SMS, mobile diary, diary integration with HC providers, picture diary, automatic step counter, food information on phone, context sensitivity, mobile BG modelling	Data transmission, education, diary, communication with healthcare providers	Blood glucose, physical exercise, food	-	Without risk assessment;<br>Without systematic clinical consideration in each functionality (ie, education, diary);
Demidowitch 2012 [9]	42 Android apps	Features	SMBG recording, a tool to track insulin or oral diabetic medications, a prandial insulin dose calculator, track bodyweight or blood pressure, graphing, data exporting or synchronisation with PHR	Track and recording, insulin dose calculator, graphing, data exporting, data synchronisation with PHR	SMBG, bodyweight, blood pressure, medications and insulin	-	Without risk assessment;
Holtz 2012 [10]	21 studies	Functions	Diary/log to record data such as blood glucose readings, carbohydrate or calorie consumption or physical activity, reminders, information/education, Bluetooth/wire/signal/manually data transmission between phone and glucometer	Diary, reminders, education, data transmission	Blood glucose, carbohydrate, calorie consumption, physical activity	-	Without risk assessment;<br>Broad definition of functionalities without descriptive details;<br>Without systematic clinical consideration in each functionality (ie, education, reminders);
Liang 2011 [11]	22 studies	Interventions	Provide support for SMBG, continuous education, reinforcement of diet, exercise and medication adjustment	Education	SMBG, diet, exercise, medication and	-	Without risk assessment;<br>Broad definition of functionalities;

					insulin		
Rao 2010 [12]	22 iPhone apps	Features	Glucose/carbohydrate/insulin/medicine/activity/ weight/blood pressure tracking, meal-time tagging, preset/custom note, food database, color coded for hypo/hyper, trend chart length/widescreen mode/logbook view/direct entry from logbook/background themes, averages/standard deviation, email composer (comma-separated values), target range settings, auto synch to website	Tracking, tagging/notes/colour coded, database, multiple displays, manual entry, multiple statistics, data exportation (email)	Blood glucose, diet, exercise, weight, blood pressure, medication and insulin	-	Without risk assessment; Duplicated functionalities due to narrow definition of each one;

C Development of the preliminary taxonomy

Functional axis

**Functionalities [1-12]**	**Structured classification framework**	
	**Functional modules**	**Descriptive Details**
Uploading blood glucose, blood pressure, medication and other information by self-log or paired medical devices;	Log	Collecting personal parameters and information from mobile device itself or from separate devices connected to it;<br>The basic functionality of mobile health technology for management of diabetes.
Visualizing physiological, and medical data;<br>Log book;<br>Display patterns of fluctuations;<br>Add tags and notes;<br>Export and share	Structured display	Organizing tracked data in a structured way
Diabetes recipes, disease information, instructions for self-management and behaviour change through electronic educational materials on the storage, offline database, email, text message, video or website;	General education	Diabetes self-management education
Real-time reminders and feedback for entered data;<br>Medication and insulin regimen adjustment and recommendations;<br>Management dedicated decision support software;<br>Clinical decision support system;	Personalized feedback	Processing tracked data and generating personalized goals, reminders and feedback instantly.
Patient-patient communication;<br>Video conferencing with clinicians;<br>Messaging the clinicians;<br>Providing feedback to patients by the clinicians;	Communication	General communication, connecting users with their peers and families through social networking, chat forums, or websites;<br>Patient-clinician communication, in-app access to healthcare providers for medical support or consultation.

Clinical axis

ADA Foundations of Care [13]	Structured classification framework	
	**Diabetes management modules**	**Descriptive Details**
SMBG	Monitoring	Self-monitoring parameters including blood glucose and blood pressure.<br>Other medical parameters including cholesterol levels, HbA1c, urine and blood testing.<br>The basic functionality of mobile health technology for management of diabetes.
Immunization	Medication management	Medications for diabetes including insulin, oral antidiabetic agents, aspirin, antihypertensive, lipid-lowering medications, and vaccinations.
Nutrition	Lifestyle modification	Healthy eating and being active.
Physical Activity		
Education	-	A functional component
Smoking Cessation	Complication prevention	Prevent diabetes-related chronic and acute complications. Acute complications include hypoglycemia and hyperglycemia; chronic complications include cardiovascular disease and microvascular complications (ie, nephropathy, retinopathy, neuropathy).
Psychosocial Care	Psychosocial care	Physical and emotional care

Risk axis

FDA risk-based framework [14]		Structured classification framework	
**Functionalities**	**Risk recommendations**	**Functions**	**Risk Assessment**
Scheduling;<br>Analysis of historical claims data to predict future utilisation or cost-effectiveness;<br>Population health management;<br>General purpose communications;	Pose limited or no risk to patient safety;	Track self-monitoring parameters (ie. blood glucose and blood pressures);<br>Structured display;<br>General education of instructions for monitoring, lifestyle modification and psychosocial care;<br>Personalized feedback concerning customised reminders;<br>Patient-patient and patient-family communication;	Low risk
Health information and data management;<br>Data capture and encounter documentation;<br>Electronic access to clinical results;<br>Some clinical decision support;<br>Medication management (electronic medication administration records);<br>Electronic communication and coordination (i.e. provider to patient, patient to provider, etc.)<br>Knowledge (clinical evidence) management;	Potential safety risks are generally low compared to the potential benefits;	Track profession-monitoring parameters (ie, cholesterol levels, HbA1c, urine and blood testing);<br>General educations of interpreting parameters, treatment and medications and complication prevention;<br>Personalized feedback concerning target setting and self-management decision making in lifestyle modification;<br>Patient-clinicians communication;	Potential risk
Most Drug dosing calculations;<br>Medical-device accessories;<br>Clinical decision support;<br>Suggestions for possible diagnoses;	Present greater risks to patient safety;	Clinical decision making on adjusting to current treatment (eg, oral agents, and insulin) by mobile health algorithms alone without the involvement of healthcare providers.	High risk

D Validation of the structured classification framework by on-market apps review

Flow chart for selection of apps

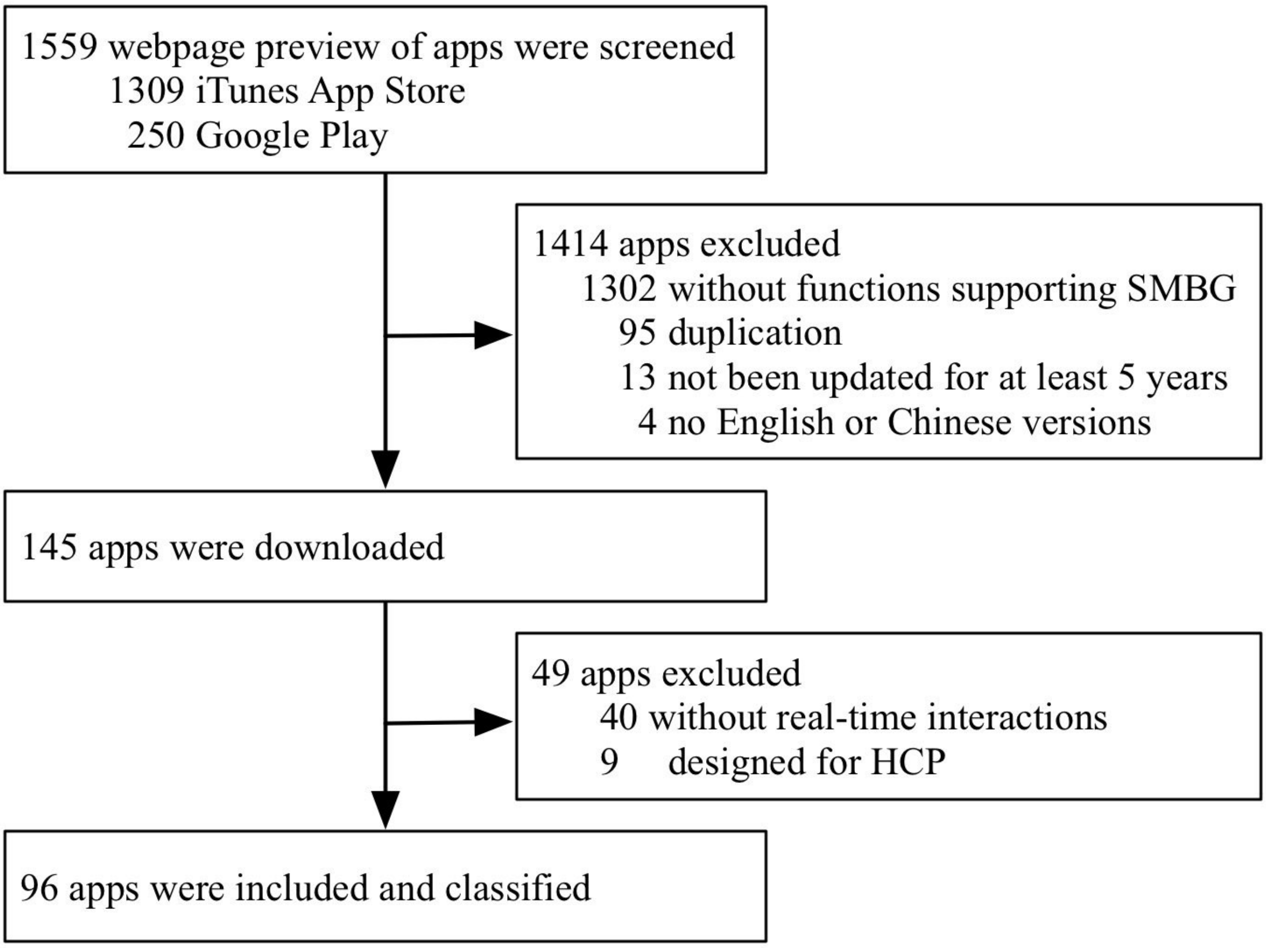

SMBG, self-monitoring of blood glucose. HCP, health care providers.

Structured classifications for functions of included apps

AADE Diabetes Goal Tracker

Functions	Functional modules	Diabetes management modules	Risk assessment
Share goals with the community;<br>Support each other by sending each other push notifications;<br>Link-up over Facebook;<br>Award points to others by accepting their support;	Communication	-	Low Risk
Stay on track with your goals;	Track	Monitoring	
Customizable reminders ;	Personalized feedback	Monitoring	Low Risk
Edit your daily totals in profile settings under Recommended Daily Allowance to individualize goals;	Personalized feedback	Lifestyle modification	Potential risk
Learn about what you're eating to better manage your diabetes;<br>Search for common foods and restaurant items or scan the barcode to see nutritional values;<br>Log items under breakfast, lunch, dinner or snacks to view your total intake of calories, carbs, sodium and total fats for each day;	General education	Lifestyle modification	Low risk
Focus on areas of diabetes management: healthy eating, being active, monitoring, taking medication, problem solving, reducing risks and healthy coping.	General education	Monitoring; Lifestyle modification; Medications; Complication prevention; Psychosocial care	Potential risk

Diabetes Tracker

Functions	Functional modules	Diabetes management modules	Risk assessment
Track blood glucose;<br>Track blood pressure;	Track	Monitoring	Low risk
Keep a track of your Insulin intake and all your Medicines.	Track	Medications	Potential risk
Diet Diary	Track	Lifestyle modification	
View current, detailed or average readings in a list view or via detailed graphs and reports	Structured display	-	Low risk
Reminders to check Blood Glucose	Personalized feedback	Monitoring	Low risk
Reminders to track medicines	Personalized feedback	Medications	Potential risk
Reminders of appointments	Personalized feedback	Complication prevention	Low risk
Reminders to maintain exercise schedules	Personalized feedback	Lifestyle modification	Low risk
Diet Guidelines;<br>Exercises with proper instructions and images;	General education	lifestyle modification	Low risk
Maintain an extensive list of Doctors and call them from	Communication	-	Potential risk

within the app.			

SocialDiabetes

**Functions**	**Functional modules**	**Diabetes management modules**	**Risk assessment**
Track blood glucose;	Track	Monitoring	Low risk
Track food;	Track	Lifestyle modification	Low risk
Track insulin;	Track	Medications	Potential risk
Graphs;	Structured display	-	Low risk
Insulin correction recommendations;	Personalized feedback	Medications	High risk
Food database with nutrition;	General education	Lifestyle modification	Low risk
Advice to prevent hypoglycemia;	General education	Complication prevention	Potential risk
Share with other users;	Communication	-	Low risk
ACE Medical Device, Council Directive 93/42/EEC that meets the highest standards of safety and quality.			

Diabetes Tracker

**Functions**	**Functional modules**	**Diabetes management modules**	**Risk assessment**
Comprehensive blood glucose tracking;<br>BP tracking;	Track	Monitoring	Low risk
HbA1c, LDL, HDL tracking;	Track	Monitoring	Potential risk
Optional insulin tracking<br>Medication tracking	Track	Medications	Potential risk
Food and carb tracking;<br>Exercise tracking;<br>Tracks your water intake;	Track	Lifestyle modification	Low risk
Labels per reading supported;<br>Detailed daily and weekly analysis, charts and reports;	Structured Display	-	Low risk
Highlights out-of-range readings;<br>Practical BG reminders;<br>Optional meal reminders;	Personalized feedback	Monitoring	Low risk
Custom pre- and post-meal target ranges;	Personalized feedback	Monitoring	Potential risk
Diet planning tools	Personalized feedback	Lifestyle modification	Potential risk
Provide personalized diet advice, recommend articles and blogs, suggest tips and tricks	General education	Lifestyle modification	Low risk
Community supported by a Registered Dietitian	Communication	-	High risk
DISCLAIMER<br>Diabetes Tracker by MyNetDiary is an educational tool, it is not a medical device, it is not a substitute for a medical device or medical care, and it does not provide diagnosis, cure, mitigation, treatment, or prevention of any disease.<br>If you have questions or concerns about individual health matters or management of your diabetes, please consult your diabetes care team.			

RapidCalc Diabetes Manager

Functions	Functional modules	Diabetes management modules	Risk assessment
Track blood glucose;	Track	Monitoring	Low risk
Estimates HbA1c;	Track	Monitoring	Potential risk
Track insulin;	Track	Medications	Potential risk
Track carbohydrates;	Track	Lifestyle modification	Low risk
Charting of blood glucose readings and trend data<br>Pre and post meal BG comparisons<br>Summary statistics for doses, glucose and carbohydrates	Structured display	-	Low risk
Customised target	Personalized feedback	Monitoring	Potential
Calculate insulin	Personalized feedback	Medications	High risk
safety warnings and guidance	General education	Complication prevention	Potential risk
SAFETY INFORMATION<br>RapidCalc is intended for guidance only and is not a substitute for professional medical advice. Always discuss use of the calculator with your health care professional and only use personal settings agreed with them. If you are in any doubt as to the correct insulin dose always follow the advice of your healthcare professional.			

Diabetes Blood & Pressure Log

Functions	Functional modules	Diabetes management modules	Risk assessment
Track blood glucose and blood pressure;	Track	Monitoring	Low risk
Track HbA1c;	Track	Monitoring	Potential risk
Track insulin;	Track	Medications	Potential risk
Track meal, carbohydrates calories and other nutrients;<br>Track exercise;<br>Track weight	Track	Lifestyle modification	Low risk
Trend and timeline of health vitals;<br>Add notes and remind yourself important things about health;	Structured display	-	Low risk
Remind to monitor	Personalized feedback	Monitoring	Low risk
Remind about medicines	Personalized feedback	Medications	Potential risk
Analysis of Health records/ Medical reports;	General education	Monitoring	Potential risk
Tips for diabetes, hypertension and weight loss;	General education	Monitoring, Lifestyle modification	Low risk
Track your entire families health;<br>Remotely monitor your parents;<br>Weekly reports and alerts families health;	Communication	-	Low risk

Care4life Diabetes

Functions	Functional modules	Diabetes management	Risk

		modules	assessment
Track blood glucose;	Track	Monitoring	Low risk
Track step;	Track	Lifestyle modification	Low risk
Graph;	Structured display	-	Low risk
Set reminders for appointments;	Personalized feedback	Complication prevention	Low risk
Set reminders for medications;	Personalized feedback	Medications	Potential risk
Healthy recipes;<br>Exercise tips;	General education	Lifestyle modification	Low risk
Care4life content was developed in collaboration with the American Diabetes Association and uses text messages, a free app, videos, email and online website.			

Diabetes UK Tracker

**Functions**	**Functional modules**	**Diabetes management modules**	**Risk assessment**
Track blood glucose and blood pressure;	Track	Monitoring	Low risk
Track HbA1c, ketones and lipid profile;	Track	Monitoring	Potential risk
Track insulin;	Track	Medications	Potential risk
Track meal;<br>Track weight;	Track	Lifestyle modification	Low risk
Track feelings;	Track	Psychosocial care	Low risk
Track appointments;	Track	Complication prevention	Low risk
Graph;<br>Note;	Structured display	-	Low risk
Reminders;	Personalized feedback	-	Low risk
Share graph and notes on Facebook and Twitter	Communication	-	Low risk

One drop for Diabetes Management

**Functions**	**Functional modules**	**Diabetes management modules**	**Risk assessment**
Track blood glucose;	Track	Monitoring	Low risk
Track medications;	Track	Medications	Potential risk
Track food;<br>Track exercise;	Track	Lifestyle modification	Low risk
Tags and notes;	Structured display	-	Low risk
Customised reminders;	Personalized feedback	-	Potential risk
Food tips;	General education	Lifestyle modification	Low risk
Share in community	Communication	-	Low risk

My Diabetes Coach

**Functions**	**Functional modules**	**Diabetes management**	**Risk**

		modules	assessment
Track blood glucose;	Track	Monitoring	Low risk
Track medication;	Track	Medications	Potential risk
Track diet;<br>Track activity;	Track	Lifestyle modification	Low risk
Track feet status	Track	Complication prevention	Low risk
Chats;	Structured display	-	Low risk
Customised health goals;	Personalized feedback	-	Potential risk
Virtual coach;<br>Health information;	General education	-	Potential risk

Diamedic

**Functions**	**Functional modules**	**Diabetes management modules**	**Risk assessment**
Track blood glucose and blood pressure;	Track	Monitoring	Low risk
Track lab result;	Track	Monitoring	Potential risk
Track insulin and medications;	Track	Medications	Potential risk
Track carb intake;<br>Track exercise;<br>Track weight;	Track	Lifestyle modification	Low risk
Track feet status	Track	Complication prevention	Low risk
Graphs;<br>Composite view;<br>Notes;	Structured display	-	Low risk
Medications refill reminders;	Personalized feedback	Medications	Potential risk
Dosage calculator;	Personalized feedback	Medications	High risk
Exercise database;	General education	Lifestyle modification	Low risk
Insulin and medications database;<br>Insulin injection templates;	General education	Medications	Potential risk

Tactio Health

**Functions**	**Functional modules**	**Diabetes management modules**	**Risk assessment**
Track blood glucose and blood pressure;	Track	Monitoring	Low risk
Track cholesterol, triglycerides, HDL and LDL;	Track	Monitoring	Potential risk
Track meal, carbohydrates calories and other nutrients;<br>Track steps and activity;<br>Track weight, height, waist size, BMI;<br>Track water intake;	Track	Lifestyle modification	Low risk

Data presented by charts, computed factors and color coding;	Structured display	-	Low risk
Customised reminder;<br>Healthy goals;	Personalized feedback	-	Potential risk
A personalized nutrition plan	Personalized feedback	Lifestyle modification	Potential risk
Education about the disease, medications and lifestyle	General education	Medications; Lifestyle modifications;	Potential risk
In light of the recent final guidance provided by the FDA on mobile medical apps, we have decided to temporarily disable the insulin adjustments for patients with Diabetes using insulin in Nov 20, 2013			

PredicBGL

**Functions**	**Functional modules**	**Diabetes management modules**	**Risk assessment**
Track blood glucose;	Track	Monitoring	Low risk
HbA1c estimate;	Track	Monitoring	Potential risk
Track insulin;	Track	Medications	Potential risk
Track meal, carbohydrates calories and other nutrients;<br>Track exercise;	Track	Lifestyle modification	Low risk
Graphs with blood glucose curves;	Structured display	-	Low risk
Calculate insulin;<br>Basal Rates for insulin pump users;	Personalized feedback	Medications	High risk
Food databases with glycemic index;	General education	Lifestyle modification	Low risk
Explain blood glucose readings;	General education	Monitoring	Potential risk
SAFETY INFORMATION<br>PredictBGL is intended for guidance only and is not a substitute for professional medical advice. Always discuss use of PredictBGL with a diabetes educator or physician and only use personal settings agreed with them. If in any doubt as to the correct insulin dose always follow the advice of a Health Care Professional.			

mySugr Diabetes Logbook

**Functions**	**Functional modules**	**Diabetes management modules**	**Risk assessment**
Track blood glucose and blood pressure;	Track	Monitoring	Low risk
Estimated HbA1c;<br>Track ketones	Track	Monitoring	Potential risk
Track medications and insulin;	Track	Medications	Potential risk
Track meal, carbohydrates calories and other nutrients;<br>Track steps and activities;<br>Track weight;	Track	Lifestyle modification	Low risk
Daily, weekly, monthly analysis;	Structured display	-	Low risk
Personal therapy goals;<br>Motivating feedback;	Personalized feedback	Monitoring	Low risk

Remind to monitor;			
Basal rates for insulin pump users;<br>Bolus Calculator;	Personalized feedback	Medications	High risk
Social sharing functions;	Communication	-	Low risk
Registered (class 1) medical device			

Diabetes App/lite

**Functions**	**Functional modules**	**Diabetes management modules**	**Risk assessment**
Track blood glucose and blood pressure;	Track	Monitoring	Low risk
Track medications and insulin;	Track	Medications	Potential risk
Track meal, carb intake, calories, other nutrients and water consumption;<br>Track activities;<br>Track weight and BMI;	Track	Lifestyle modification	Low risk
Put tracked parameters on the calendar;	Structured display	-	Low risk
Off-line food database	General education	Lifestyle modification	Low risk
Budget daily carb allowance	Personalized feedback	Lifestyle modification	Potential risk

t:simulator

**Functions**	**Functional modules**	**Diabetes management modules**	**Risk assessment**
Track blood glucose and blood pressure;	Track	Monitoring	Low risk
Track insulin;	Track	Medications	Potential risk
Customised note;<br>Trends;<br>View insulin history	Structured display	-	Low risk
Set reminder alarms for low and high blood glucose, and after bolus blood glucose	Personalized feedback	Monitoring	Low risk
Set reminder alarms for missed meal boluses	Personalized feedback	Medications	Low risk
Create customised basal rates, carb ratios, correction factors, and target Blood Glucose (BG) levels;<br>Program a meal bolus, using either units of insulin or grams of carbohydrate;<br>Program a correction bolus by entering a BG value;<br>Program an extended bolus;<br>Set a temporary basal rate;	Personalized feedback	Medications	High risk
Technical specifications, glossary, and important safety information;	General education	Medications	Potential risk
social media share	Communication	-	Low risk
Contact a Tandem Diabetes Care representative directly	Communication	-	Potential risk

from within the app			
Tandem pump			

LogFrog DB/Lite

**Functions**	**Functional modules**	**Diabetes management modules**	**Risk assessment**
Track blood glucose and blood pressure;	Track	Monitoring	Low risk
Track HbA1c;	Track	Monitoring	Potential risk
Track insulin and oral medication;	Track	Medications	Potential risk
Track carb intake;<br>Track exercise;<br>Track weight;	Track	Lifestyle modification	Low risk
Track doctor visits;	Track	Complication prevention	Low risk
Customised notes;<br>Graph by days or out to months ;	Structured display	-	Low risk
Set reminders to check blood glucose;<br>Set your ideal blood glucose value range;	Personalized feedback	Monitoring	Low risk
Set reminders to take medications;	Personalized feedback	Medications	Potential risk

HEALTheDiabetes

**Functions**	**Functional modules**	**Diabetes management modules**	**Risk assessment**
Track blood glucose;	Track	Monitoring	Low risk
Track insulin and oral medication;	Track	Medications	Potential risk
Track food, calories, carbs, and nutrition;<br>Track activities;<br>Track weight;	Track	Lifestyle modification	Low risk
Charts and trends;	Structured display	-	Low risk
Set targets: carbohydrate, calorie;	Personalized feedback	Monitoring	Potential risk
Set reminders to take medications;	Personalized feedback	Medications	Potential risk
Food database with nutrition;	General education	Lifestyle modification	Low risk

Diabetes Management

**Functions**	**Functional modules**	**Diabetes management modules**	**Risk assessment**
Track blood glucose;	Track	Monitoring	Low risk
Track diet, calories, carbohydrates;<br>Track activities;	Track	Lifestyle modification	Low risk
Charts and trends;	Structured display	-	Low risk
Food database with nutrition and glycemic index;<br>A healthy diet;	General education	Lifestyle modification	Low risk

Symptoms of diabetes;	General education	Complication prevention	Potential risk
Order tests and exams;<br>Order medicine and ancillary needs;	Communication	-	Potential risk

BG Monitor Diabetes /Pro

**Functions**	**Functional modules**	**Diabetes management modules**	**Risk assessment**
Track blood glucose;	Track	Monitoring	Low risk
Track insulin;	Track	Medications	Potential risk
Track carbohydrates;<br>Track activities;	Track	Lifestyle modification	Low risk
Detailed graphs;<br>Graphical reporting;<br>Notes and tags;	Structured display	-	Low risk
Reminders of low and high blood glucose;<br>Reminders to check blood glucose;	Personalized feedback	Monitoring	Low risk
Reminders to give insulin;	Personalized feedback	Medications	Potential risk
Insulin calculator;	Personalized feedback	Medications	High risk
Food database;<br>Carbohydrates calculator;	General education	Lifestyle modification	Low risk

Track3

**Functions**	**Functional modules**	**Diabetes management modules**	**Risk assessment**
Track blood glucose;	Track	Monitoring	Low risk
Track HbA1c	Track	Monitoring	Potential risk
Track insulin and medications;	Track	Medications	Potential risk
Track food;<br>Track activities;<br>Track calories<br>Track weight;	Track	Lifestyle modification	Low risk
Chart blood glucose trends;<br>Tags;	Structured display	-	Low risk
Customised information for the foods and exercises;	Personalized feedback	Lifestyle modification	Low risk
Customised information for medications;	Personalized feedback	Medications	Potential risk
Food database with carbohydrate and nutrition details;<br>Carb and calorie counter;	General education	Lifestyle modification	Low risk

iFORA Diabetes Manager

**Functions**	**Functional modules**	**Diabetes management modules**	**Risk assessment**

Track blood glucose;	Track	Monitoring	Low risk
Track insulin dosage;	Track	Medications	Potential risk
Track activities;<br>Track food intake;	Track	Lifestyle modification	Low risk
Statistic trend graph;<br>Target pie charts;<br>Record list;<br>Logbook;	Structured display	-	Low risk
Set target goal of blood glucose;<br>Color coded alert;	Personalized feedback	Monitoring	Low risk
Diabetes medications	General education	Medications	Potential risk
Healthy eating;<br>Regular exercise routine;	General education	Lifestyle modification	Low risk

ezbds

**Functions**	**Functional modules**	**Diabetes management modules**	**Risk assessment**
Track blood glucose;	Track	Monitoring	Low risk
Track insulin dosage and medications;	Track	Medications	Potential risk
Track meal, snack and carbs;<br>Track activities;<br>Track food intake;	Track	Lifestyle modification	Low risk
Graph;<br>Tag;	Structured display	-	Low risk
Trending up alert;	Personalized feedback	Monitoring	Low risk
Insulin doses alert;	Personalized feedback	Medications	Potential risk
Drug dose calculating;	Personalized feedback	Medications	High risk
Healthy diet;	General education	Lifestyle modification	Low risk

Diabetes Kit Blood Glucose Logbook

**Functions**	**Functional modules**	**Diabetes management modules**	**Risk assessment**
Track blood glucose and blood pressure;	Track	Monitoring	Low risk
Track HbA1c;	Track	Monitoring	Potential risk
Track insulin and medication;	Track	Medications	Potential risk
Track nutrition;<br>Track steps, activities and calories;<br>Track weight;	Track	Lifestyle modification	Low risk
Trend;	Structured display	-	Low risk
Insulin dose calculator;	Personalized feedback	Medications	High risk

Clinical insight;	General education	-	Potential risk

Diabetes PA

**Functions**	**Functional modules**	**Diabetes management modules**	**Risk assessment**
Track blood glucose and blood pressure;	Track	Monitoring	Low risk
Track HbA1c, cholesterol and ketones;	Track	Monitoring	Potential risk
Track insulin and medication;	Track	Medications	Potential risk
Track mood and emotions;	Track	Psychosocial care	Low risk
Track carbs and calories;<br>Track alcohol consumption;<br>Track exercise;<br>Track weight and BMI;	Track	Lifestyle modification	Low risk
Charts;<br>Graphs;	Structured display	-	Low risk
Reminders for medications;	Personalized feedback	Medications	Potential risk

Glucose Monitor

**Functions**	**Functional modules**	**Diabetes management modules**	**Risk assessment**
Track blood glucose and blood pressure;	Track	Monitoring	Low risk
Track cholesterol;	Track	Monitoring	Potential risk
Track medications and insulin;	Track	Medications	Potential risk
Statistics;<br>Graphs;	Structured display	-	Low risk
Customised reminders for checking blood glucose;	Personalized feedback	Monitoring	
Tips on improving glycemic control;<br>Diabetes health guide;	General education	-	Potential risk
Diabetes communities	Communication	-	Low risk

Diabetes in Check

**Functions**	**Functional modules**	**Diabetes management modules**	**Risk assessment**
Blood glucose tracking;	Track	Monitoring	Low risk
Medication tracking;	Track	Medications	Potential risk
Exercise tracking;<br>Weight tracking;<br>Track carb by searching a database of over 80,000 foods or barcode scanner;	Track	Lifestyle modification	Low risk
Set reminders to measure blood glucose;	Personalized feedback	Monitoring	Low risk

Set and track exercise goals and stay motivated;<br>A personalized daily plan to help you stay on track and achieve your goals;	Personalized feedback	Lifestyle modification	Potential risk
Set reminders to take medications;	Personalized feedback	Medications	Potential risk
Join a community of over 200,000 people who are also living with type 2 diabetes.	Communication	-	Low risk
In-app support from a Certified Diabetes Educator	Communication	-	Potential risk

Diabetes Pilot Pro

**Functions**	**Functional modules**	**Diabetes management modules**	**Risk assessment**
Records glucose and blood pressure;	Track	Monitoring	Low risk
Estimates HbA1c;	Track	Monitoring	Potential risk
Record medications;	Track	Medications	Potential risk
Records meals, exercise, weight;<br>Track carbohydrates, calories and nutrients by scanning barcodes;	Track	Lifestyle modification	Low risk
Customizable reports;<br>Notes about information;	Structured display	-	Low risk
Estimate your insulin for meals and correcting high glucose;	Personalized feedback	Medications	High risk

Daily Carb /Premium

**Functions**	**Functional modules**	**Diabetes management modules**	**Risk assessment**
Track blood glucose and blood pressure;	Track	Monitoring	Low risk
Track HbA1c;<br>Track cholesterol, triglycerides, HDL and LDL;	Track	Monitoring	Potential risk
Track medications and insulin;	Track	Medications	Potential risk
Track meal, carbohydrates calories and other nutrients;<br>Track exercise by date, duration, name and intensity;<br>Track weight;<br>Track water intake;	Track	Lifestyle modification	Low risk
Analyze tracked parameters by chart;<br>Add note to reading;<br>Calendar overview of data;	Structured display	-	Low risk
Customised reminder	Personalized feedback	-	Potential risk
Calculate insulin	Personalized feedback	Medications	High risk
For the insulin calculation function, it is based this formula:<br>Insulin dosage= (Blood Sugar – Goal Blood Sugar) / Correction Factor + Carbohydrate / Carbohydrate Factor<br>Please do consult your physician before using it to check if the formula is suitable for you, and ask for your personal Correction Factor,			

Carbohydrate Factor, Goal Blood Sugar from him/her, and please take note of the unit (mg/dL or mmol/L). The user assumes all responsibility for the numbers inputted and the result.

Glucose Wiz/pro

Functions	Functional modules	Diabetes management modules	Risk assessment
Track blood glucose;	Track	Monitoring	Low risk
Track medications;	Track	Medications	Potential risk
Track weight and BMI;	Track	Lifestyle modification	Low risk
Generates glucose chart view by daily, weekly, monthly or yearly;<br>Generates histogram to show average glucose of each period;<br>Generates chart view to show weight tendency;	Structured display	-	Low risk
Customised reminders	Personalized feedback	-	Low risk
Manage medications	Personalized feedback	Medications	Potential risk

Health2Sync

Functions	Functional modules	Diabetes management modules	Risk assessment
Track blood glucose;	Track	Monitoring	Low risk
Track HbA1c	Track	Monitoring	Potential risk
Track medications and insulin;	Track	Medications	Potential risk
Track food;	Track	Lifestyle modification	Low risk
Enter note;<br>A quick analysis of your recent blood sugar history in table and graph formats;	Structured display	-	Low risk
Invite families to become partners with you on Health2Sync	Communication	-	Low risk

HealthWatch 360

Functions	Functional modules	Diabetes management modules	Risk assessment
Track blood glucose and blood pressure;	Track	Monitoring	Low risk
Track triglycerides	Track	Monitoring	Potential risk
Track medications and insulin;	Track	Medications	Potential risk
Track food, calories and nutrients;<br>Track weight;	Track	Lifestyle modification	Low risk
Customised note;<br>Trends;	Structured display	-	Low risk
Food nutrition score, charts and tips;	General education	Lifestyle modification	Low risk

Customised recipe;			

Diabetes, blood pressure Diary

Functions	Functional modules	Diabetes management modules	Risk assessment
Track blood glucose and blood pressure;	Track	Monitoring	Low risk
Estimated HbA1c;	Track	Monitoring	Potential risk
Track insulin and oral medication;	Track	Medications	Potential risk
Track food;<br>Track weight;	Track	Lifestyle modification	Low risk
Graph for trends;	Structured display	-	Low risk
Set reminders to take medications	Personalized feedback	Medications	Potential risk

Diabetes Glucose Tracker App

Functions	Functional modules	Diabetes management modules	Risk assessment
Track blood glucose;	Track	Monitoring	Low risk
Track HbA1c;	Track	Monitoring	Potential risk
Track insulin and oral medication;	Track	Medications	Potential risk
Track food consumption and carbs;<br>Track physical activities;<br>Track weight;	Track	Lifestyle modification	Low risk
Charts and trends;	Structured display	-	Low risk
Set goal;	Personalized feedback	Monitoring	Potential risk
Set reminders to take medications;	Personalized feedback	Medications	Potential risk
Clinical Study Reference<br>Evan M. Benjamin. Self-Monitoring of Blood Glucose: The Basics. Clinical Diabetes January 2002 20:45-47; doi:10.2337/diaclin.20.1.45			

Diabetes Plus

Functions	Functional modules	Diabetes management modules	Risk assessment
Track blood glucose and blood pressure;	Track	Monitoring	Low risk
Calculation of HbA1c;	Track	Monitoring	Potential risk
Track insulin and medication;	Track	Medications	Potential risk
Track carbohydrates;<br>Track activities;<br>Track weight;	Track	Lifestyle modification	Low risk
Line graphs;<br>Graphical reporting;<br>Notes;	Structured display	-	Low risk
Customised glucose target range;	Personalized feedback	Monitoring	Low risk

Colored indicators;			
Calculation of dosage of insulin;	Personalized feedback	Medications	High risk

Diaguard Diabetes

**Functions**	**Functional modules**	**Diabetes management modules**	**Risk assessment**
Track blood glucose and blood pressure;	Track	Monitoring	Low risk
Track HbA1c	Track	Monitoring	Potential risk
Track insulin;	Track	Medications	Potential risk
Track carbohydrates;	Track	Lifestyle modification	Low risk
Visualize blood glucose in a graph;<br>Detailed logs of your data;	Structured display	-	Low risk
Reminders;	Personalized feedback	-	Potential risk
Bolus calculator;	Personalized feedback	Medications	High risk

Glucose Buddy/Pro

**Functions**	**Functional modules**	**Diabetes management modules**	**Risk assessment**
Track blood glucose and blood pressure;	Track	Monitoring	Low risk
Track HbA1c	Track	Monitoring	Potential risk
Track insulin;	Track	Medications	Potential risk
Track carbohydrates;<br>Track activities;<br>Track weight;	Track	Lifestyle modification	Low risk
Graph of food, activities and blood glucose;<br>Tags;	Structured display	-	Low risk
Reminders;	Personalized feedback	-	Potential risk

Diabetes Pal App

**Functions**	**Functional modules**	**Diabetes management modules**	**Risk assessment**
Track blood glucose and blood pressure;	Track	Monitoring	Low risk
Track HbA1c	Track	Monitoring	Potential risk
Track medications;	Track	Medications	Potential risk
Track nutrition;<br>Track activities;<br>Track calories<br>Track weight;	Track	Lifestyle modification	Low risk
Charts and graphs;	Structured display	-	Low risk

Tags;<br>Notes;			
Create and achieve goals;	Personalized feedback	-	Potential risk

BlueStar Diabetes

**Functions**	**Functional modules**	**Diabetes management modules**	**Risk assessment**
Track blood glucose and blood pressure;	Track	Monitoring	Low risk
Track HbA1c and cholesterol;	Track	Monitoring	Potential risk
Track medications;	Track	Medications	Potential risk
Track diet;<br>Track activities;	Track	Lifestyle modification	Low risk
Charts and graphs;	Structured display	-	Low risk
Personalized guidance driven by medication treatment plan and blood glucose;	Personalized feedback	Medications	Hihg risk
Prescribed by a healthcare provider.			

iBGStar

**Functions**	**Functional modules**	**Diabetes management modules**	**Risk assessment**
Track blood glucose;	Track	Monitoring	Low risk
Track insulin doses;	Track	Medications	Potential risk
Track carb;<br>Track activities;	Track	Lifestyle modification	Low risk
Trend chart, logbook and statistics;<br>Tag of mealtimes;<br>Customised notes;	Structured display	-	Low risk
Color-coded hypo and hyper alerts when blood glucose readings are out of range	Personalized feedback	Monitoring	Low risk

myMedtronic Connect

**Functions**	**Functional modules**	**Diabetes management modules**	**Risk assessment**
Track blood glucose and infusion site;	Track	Monitoring	Low risk
Track insulin doses;	Track	Medications	Potential risk
Graph;	Structured display	-	Low risk
Set reminders;<br>Alarms and alerts;	Personalized feedback	-	Low risk
Video tutorials;<br>Tips and explanations;<br>Correct infusion set placement and site rotations;	General education	-	Potential risk

Medtronic diabetes blog;			
A support tool for a Medtronic insulin pump and continuous glucose monitor (CGM)			

Glucool Diabetes/Premium

**Functions**	**Functional modules**	**Diabetes management modules**	**Risk assessment**
Track blood glucose and blood pressure;	Track	Monitoring	Low risk
Track HbA1c;	Track	Monitoring	Potential risk
Track insulin and medication;	Track	Medications	Potential risk
Track carbs intake;<br>Track exercise;<br>Track weight and BMI;	Track	Lifestyle modification	Low risk
Graph;<br>Pie charts;<br>Note;	Structured display	-	Low risk
Customised ranges;<br>Remind to check blood glucose;	Personalized feedback	Monitoring	Low risk
Insulin dose calculator;	Personalized feedback	Medications	High risk

Glucose Companion/Free

**Functions**	**Functional modules**	**Diabetes management modules**	**Risk assessment**
Track blood glucose;	Track	Monitoring	Low risk
Track weight;	Track	Lifestyle modification	Low risk
Chart;<br>Note;<br>Histogram;<br>Statistics;	Structured display	-	Low risk
Customised target range;	Personalized feedback	Monitoring	Low risk
Customised reminders;	Personalized feedback	-	Potential risk
Insulin dose calculator;	Personalized feedback	Medications	High risk
For the insulin calculation function, it is based this formula: Insulin dosage= (Blood Sugar – Goal Blood Sugar)/ Correction Factor+ Carbohydrate /Carbohydrate Factor.<br>Please do consult your physician before using it to check if the formula is suitable for you, and ask for your personal Correction Factor, Carbohydrate Factor, Goal Blood Sugar from him/her, and please take note of the unit (mg/dl or mmoL/L). The user assumes all responsibility for the numbers inputted and the result.			

Diabetes in Pregnancy

**Functions**	**Functional modules**	**Diabetes management**	**Risk**

		**modules**	**assessment**
Track blood glucose;	Track	Monitoring	Low risk
Track food, carbs and calorie;	Track	Lifestyle modification	Low risk
Track insulin and medications;	Track	Medications	Potential risk
Charts;	Structured display	-	Low risk
Information for food, nutrition and exercises	General education	Lifestyle modification	Low risk
Information for medications	General education	Medications	Potential risk

Glooko

**Functions**	**Functional modules**	**Diabetes management modules**	**Risk assessment**
Track blood glucose and blood pressure;	Track	Monitoring	Low risk
Track insulin;	Track	Medications	Potential risk
Track carbs;<br>Track exercise;<br>Track weight;	Track	Lifestyle modification	Low risk
Trends;<br>Graph;<br>Statistics;	Structured display	-	Low risk
Customised reminders	Personalized feedback	-	Potential risk
FDA 510(k) cleared platform			

Carburetor

**Functions**	**Functional modules**	**Diabetes management modules**	**Risk assessment**
Track blood glucose;	Track	Monitoring	Low risk
Track HbA1c	Track	Monitoring	Potential risk
Track medications;	Track	Medications	Potential risk
Track carbs;<br>Track exercise;	Track	Lifestyle modification	Low risk
Statistics;	Structured display	-	Low risk
Food nutrition;	General education	Lifestyle modification	Low risk

Diabetes:360

**Functions**	**Functional modules**	**Diabetes management modules**	**Risk assessment**
Track blood glucose;	Track	Monitoring	Low risk
Track insulin;	Track	Medications	Potential risk
Track carbohydrate;<br>Track exercise;	Track	Lifestyle modification	Low risk
Graphs;	Structured display	-	Low risk

Customised reminder	Personalized feedback	-	Potential risk
Calculate insulin dosing	Personalized feedback	Medications	High risk

ACCU-CHEK

**Functions**	**Functional modules**	**Diabetes management modules**	**Risk assessment**
Track blood glucose and blood pressure;	Track	Monitoring	Low risk
Track insulin and medications;	Track	Medications	Potential risk
Track meals, size, carbs and calories;<br>Track exercise;<br>Track weight;	Track	Lifestyle modification	Low risk
Trends;<br>Graphs;	Structured display	-	Low risk
Reminders for blood glucose testing;	Personalized feedback	Monitoring	Low risk
Bolus insulin advice;	Personalized feedback	Medications	High risk
The ACCU-CHEK Bolus Advisor feature must be activated by your healthcare professional with appropriate information needed for set-up and use.			

Gestational Diabetes Manager

**Functions**	**Functional modules**	**Diabetes management modules**	**Risk assessment**
Track blood glucose;	Track	Monitoring	Low risk
Track ketones;	Track	Monitoring	Potential risk
Track insulin;	Track	Medications	Potential risk
Track carbohydrate intake;<br>Track exercise;<br>Track water consumption;	Track	Lifestyle modification	Low risk
Charts;<br>Graphs;	Structured display	-	Low risk
Food database with nutrition and calorie information;	General education	Lifestyle modification	Low risk

Sugar Sense

**Functions**	**Functional modules**	**Diabetes management modules**	**Risk assessment**
Track blood glucose;	Track	Monitoring	Low risk
Track nutrition;<br>Track activities;<br>Track weight;	Track	Lifestyle modification	Low risk
Charts;<br>Trends;<br>Graphs;	Structured display	-	Low risk

Tips on improving glycemic control;<br>Diabetes health guide;	General education	-	Low risk
Diabetes communities	Communication	-	Low risk
This software is intended for informational and educational use only. Please consult your healthcare provider for any health concerns.			

mySugr Junior

**Functions**	**Functional modules**	**Diabetes management modules**	**Risk assessment**
Track blood glucose;	Track	Monitoring	Low risk
Track medications and insulin;	Track	Medications	Potential risk
Track carbs;	Track	Lifestyle modification	Low risk
Goals and feedback	Personalized feedback	Monitoring	Low risk
Communication between children living with diabetes and their caregivers	Communication	-	Low risk

Easy Diabetes

**Functions**	**Functional modules**	**Diabetes management modules**	**Risk assessment**
Track blood glucose;	Track	Monitoring	Low risk
Track ketones;	Track	Monitoring	Potential risk
Track insulin;	Track	Medications	Potential risk
Track carbohydrate;	Track	Lifestyle modification	Low risk
Notes;<br>Charts;	Structured display	-	Low risk
Customised targets and feedback	Personalized feedback	Monitoring	Low risk

GlucoSweet

**Functions**	**Functional modules**	**Diabetes management modules**	**Risk assessment**
Track blood glucose;	Track	Monitoring	Low risk
Track carbohydrate;	Track	Lifestyle modification	Low risk
Track medications and insulin;	Track	Medications	Potential risk
Graphs and chart;<br>Statistics;	Structured display	-	Low risk
Customised ranges and feedback;	Personalized feedback	Monitoring	Low risk
Customised reminders;	Personalized feedback	-	Potential risk

Diabetes Pedometer with Glucose

**Functions**	**Functional modules**	**Diabetes management modules**	**Risk assessment**
Track blood glucose and blood pressure;	Track	Monitoring	Low risk

Track activity;<br>Carb counting;<br>Track weight and BMI;	Track	Lifestyle modification	Low risk
Track insulin;	Track	Medications	Potential risk
Graphs and chart;	Structured display	-	Low risk
Medication reminders;	Personalized feedback	Medications	Potential risk

Diabetes Recorder/Free

**Functions**	**Functional modules**	**Diabetes management modules**	**Risk assessment**
Track blood glucose and blood pressure;	Track	Monitoring	Low risk
Track HbA1c;	Track	Monitoring	Potential risk
Track insulin;	Track	Medications	Potential risk
Track carbohydrate;<br>Track movement;<br>Track weight;	Track	Lifestyle modification	Low risk
Graphs and chart;	Structured display	-	Low risk
Set alarms to measure;	Personalized feedback	Monitoring	Low risk

Diabetes studio

**Functions**	**Functional modules**	**Diabetes management modules**	**Risk assessment**
Track blood glucose;	Track	Monitoring	Low risk
Track moods and emotions;	Track	Psychosocial care	Low risk
Tags;<br>Charts;	Structured display	-	Low risk
Customised reminders;	Personalized feedback	-	Potential risk
Insulin calculator;	Personalized feedback	Medications	High risk

DiabetesConnect

**Functions**	**Functional modules**	**Diabetes management modules**	**Risk assessment**
Track blood glucose;	Track	Monitoring	Low risk
Track insulin and medications;	Track	Medications	Potential risk
Track meals and carbohydrate;<br>Track activities;	Track	Lifestyle modification	Low risk
Graphs and statistics;<br>Pie chart;	Structured display	-	Low risk
Set reminders;	Personalized feedback	Monitoring	Low risk

BlueLoop

Functions	Functional modules	Diabetes management modules	Risk assessment
Track blood glucose;	Track	Monitoring	Low risk
Track carbohydrates;	Track	Lifestyle modification	Low risk
Track medications and insulin;	Track	Medications	Potential risk
Notes;	Structured display	-	Low risk
Instant notifications are sent as enter data;	Personalized feedback	-	Potential risk

GlucoSuccess

Functions	Functional modules	Diabetes management modules	Risk assessment
Track blood glucose;	Track	Monitoring	Low risk
Track diet, sugar, carbohydrates and calories;<br>Track steps, activity duration and intensity;<br>Track body weight and waist size;	Track	Lifestyle modification	Low risk
Provide an integrated view of your health behaviors<br>Compare foods you ate and activity levels before in-range/out-of-range blood glucose measurements	Structured display	-	Low risk
Receive Notifications about:<br>- Logging your blood glucose<br>- Recording diet information through LoseIt	Personalized feedback	Monitoring;<br>Lifestyle modification	Low risk
Diabetes lifestyle education from ADA website.	General education	Lifestyle modificaiton	Low risk
A research study.			

MyGlycemia

Functions	Functional modules	Diabetes management modules	Risk assessment
Track blood glucose;	Track	Monitoring	Low risk
Table, graph and charts;	Structured display	-	Low risk
Alarm to measure blood glucose;	Personalized feedback	Monitoring	Low risk
Avoid hypoglycemia;	General education	Complication prevention	Potential risk

GoMeals

Functions	Functional modules	Diabetes management modules	Risk assessment
Track blood glucose;	Track	Monitoring	Low risk
Track food, nutrition, carbs and calorie;<br>Track activities and calories;<br>Track weight;	Track	Lifestyle modification	Low risk

Charts;<br>Tags;	Structured display	-	Low risk
Database for food and nutrition	General education	Lifestyle modification	Low risk

Diabetes Personal Calculator

**Functions**	**Functional modules**	**Diabetes management modules**	**Risk assessment**
Track blood glucose;	Track	Monitoring	Low risk
Track insulin;	Track	Medications	Potential risk
Track meals and food items, carbohydrate intake;	Track	Lifestyle modification	Low risk
Bolus insulin calculator;	Personalized feedback	Medications	High risk
Diabetes Personal Calculator must only be used as a guide and on the advice of your physician.			

Healthgoal360

**Functions**	**Functional modules**	**Diabetes management modules**	**Risk assessment**
Track blood glucose;	Track	Monitoring	Low risk
Track HbA1c and lab results;	Track	Monitoring	Potential risk
Track medications and insulin;	Track	Medications	Potential risk
Track steps and calories;<br>Track diets;<br>Track weight;	Track	Lifestyle modification	Low risk
Summary dashboard;<br>Charts;	Structured display	-	Low risk

Diabetes Assistant

**Functions**	**Functional modules**	**Diabetes management modules**	**Risk assessment**
Track blood glucose;	Track	Monitoring	Low risk
Track meals;	Track	Lifestyle modification	Low risk
Charts;	Structured display	-	Low risk
Customised targets and feedback;	Personalized feedback	Monitoring	Low risk
Customised reminders;	Personalized feedback	-	Potential risk

Diabetes Diary

**Functions**	**Functional modules**	**Diabetes management modules**	**Risk assessment**
Track blood glucose;	Track	Monitoring	Low risk
Track carbohydrate intake;	Track	Lifestyle modification	Low risk
Track insulin;	Track	Medications	Potential risk

Graphs;<br>Tags;	Structured display	-	Low risk

Diabetes:M

Functions	Functional modules	Diabetes management modules	Risk assessment
Track blood glucose;	Track	Monitoring	Low risk
Charts;<br>Statistics;	Structured display	-	Low risk
Insulin bolus calculator;	Personalized feedback	Medications	High risk

OnTrack Diabetes

Functions	Functional modules	Diabetes management modules	Risk assessment
Track blood glucose;	Track	Monitoring	Low risk
Track HbA1c;	Track	Monitoring	Potential risk
Track food;<br>Track weight;	Track	Lifestyle modification	Low risk
Graphs and chart;	Structured display	-	Low risk
Set reminders;	Personalized feedback	Monitoring	Low risk

Diabetes Management

Functions	Functional modules	Diabetes management modules	Risk assessment
Track blood glucose and blood pressure;	Track	Monitoring	Low risk
Track insulin;	Track	Medications	Potential risk
Track carbohydrate;<br>Track movement;	Track	Lifestyle modification	Low risk
Finding diabetes doctors	Communications	-	Potential risk
News for diabetes	General education	-	Potential risk

Dexcom Follow/Dexcom Share2

Functions	Functional modules	Diabetes management modules	Risk assessment
Track blood glucose;	Track	Monitoring	Low risk
Graphs and trends;	Structured display	-	Low risk
Customised glucose range and alarms;	Personalized feedback	Monitoring	Low risk
Share with families;	Communication	-	Low risk
Dexcom CGM companion			

mylife SiDiary

Functions	Functional modules	Diabetes management modules	Risk assessment
Track blood glucose and blood pressure;	Track	Monitoring	Low risk
Track carbohydrates;	Track	Lifestyle modification	Low risk
Track medications;	Track	Medications	Potential risk
Statistic graphs;<br>Trend analysis;	Structured display	-	Low risk

dbees.com

Functions	Functional modules	Diabetes management modules	Risk assessment
Track blood glucose;	Track	Monitoring	Low risk
Track diet;	Track	Lifestyle modification	Low risk
Track insulin and medications;	Track	Medications	Potential risk
Graphs;	Structured display	-	Low risk

AgaMatrix Diabetes Manager

Functions	Functional modules	Diabetes management modules	Risk assessment
Track blood glucose;	Track	Monitoring	Low risk
Track carbs;<br>Track weight;	Track	Lifestyle modification	Low risk
Track insulin and medications;	Track	Medications	Potential risk
Trends and logbook;	Structured display	-	Low risk

Best Diabetes Control

Functions	Functional modules	Diabetes management modules	Risk assessment
Track blood glucose and blood pressure;	Track	Monitoring	Low risk
Track food, categories, carbs and nutrition;<br>Track exercise and calories;<br>Track weight and BMI;<br>Track water intake	Track	Lifestyle modification	Low risk
Track insulin and medications;	Track	Medications	Potential risk
Trends and graphs;<br>Tags;	Structured display	-	Low risk

diasend

Functions	Functional modules	Diabetes management modules	Risk assessment
Track blood glucose;	Track	Monitoring	Low risk

Track activity;<br>Track carb	Track	Lifestyle modification	Low risk
Track insulin;	Track	Medications	Potential risk
Landscape mode;	Structured display	-	Low risk

SiDiary Diabetes Management

**Functions**	**Functional modules**	**Diabetes management modules**	**Risk assessment**
Track blood glucose and blood pressure;	Track	Monitoring	Low risk
Track carbohydrates;<br>Track weight;	Track	Lifestyle modification	Low risk
Track insulin and medications;	Track	Medications	Potential risk
Trends and statistics;	Structured display	-	Low risk

Diabetes Insulin Calculator

**Functions**	**Functional modules**	**Diabetes management modules**	**Risk assessment**
Track blood glucose;	Track	Monitoring	Low risk
Track food;	Track	Lifestyle modification	Low risk
Insulin calculator	Personalized feedback	Medications	High risk

Diabetes Diary

**Functions**	**Functional modules**	**Diabetes management modules**	**Risk assessment**
Track blood glucose;	Track	Monitoring	Low risk
Track insulin;	Track	Medications	Potential risk
Custom notes	Structured display	-	Low risk
Insulin calculator	Personalized feedback	Medications	High risk

Diabetes BP Health Tracker App

**Functions**	**Functional modules**	**Diabetes management modules**	**Risk assessment**
Track blood glucose and blood pressure;	Track	Monitoring	Low risk
Track food;<br>Track weight and body metrics;	Track	Lifestyle modification	Low risk
Track medications and insulin;	Track	Medications	Potential risk
Graphs;	Structured display	-	Low risk

Gluco Share

**Functions**	**Functional modules**	**Diabetes management modules**	**Risk assessment**

Track blood glucose;	Track	Monitoring	Low risk
Graphs;	Structured display	-	Low risk
Customised goals and feedback;	Personalized feedback	Monitoring	Low risk
Share with other users;	Communication	-	Low risk

Welkin for Diabetes

**Functions**	**Functional modules**	**Diabetes management modules**	**Risk assessment**
Track blood glucose;	Track	Monitoring	Low risk
Track meals;	Track	Lifestyle modification	Low risk
Connect with a professional diabetes coach;	Communication	-	Potential risk

True Manager Air Logbook

**Functions**	**Functional modules**	**Diabetes management modules**	**Risk assessment**
Track blood glucose;	Track	Monitoring	Low risk
Logbook and tag;	Structured display	-	Low risk
Set target ranges for blood glucose;	Personalized feedback	Monitoring	Potential risk
True Metrix Air Blood Glucose wireless meter companion.<br>Target Range (mg/dL) YELLOW = Above Normal >130 GREEN = Normal 70-130 RED = Hypoglycemic <70.<br>This product is intended for personal use to support better diabetes management without providing specific treatment or treatment suggestions. It is not for diagnostic use. Consult a Doctor or Healthcare Professional before changing treatment plan.			

Diabetic Connect

**Functions**	**Functional modules**	**Diabetes management modules**	**Risk assessment**
Track blood glucose;	Track	Monitoring	Low risk
Track mood;	Track	Psychosocial care	Low risk
Community of diabetes patients;	Communication	-	Low risk

Dexcom G5 Mobile

**Functions**	**Functional modules**	**Diabetes management modules**	**Risk assessment**
Track blood glucose;	Track	Monitoring	Low risk
Trends;	Structured display	-	Low risk
Set alerts for glucose	Personalized feedback	Monitoring	Potential risk
Continuous glucose monitoring system			

Sugar Streak

**Functions**	**Functional modules**	**Diabetes management modules**	**Risk assessment**

Track blood glucose;	Track	Monitoring	Low risk
Predicted HbA1c;	Track	Monitoring	Potential risk
Graphs;	Structured display	-	Low risk
Set goals and rewards;<br>Reminders to monitor;	Personalized feedback	Monitoring	Potential risk

Bant

**Functions**	**Functional modules**	**Diabetes management modules**	**Risk assessment**
Track blood glucose;	Track	Monitoring	Low risk
Graphs;	Structured display	-	Low risk
Share through Twitter;	Communication	-	Low risk

ditto Glucose Logbook

**Functions**	**Functional modules**	**Diabetes management modules**	**Risk assessment**
Track blood glucose;	Track	Monitoring	Low risk
Graphs;<br>Notes and tags;	Structured display	-	Low risk
Customised blood glucose range;	Personalized feedback	Monitoring	Low risk

Dnurse

**Functions**	**Functional modules**	**Diabetes management modules**	**Risk assessment**
Track blood glucose;	Track	Monitoring	Low risk
Graphs and statistics;	Structured display	-	Low risk
Customised reminders;	Personalized feedback	Monitoring	Low risk

Blood Glucose Tracker

**Functions**	**Functional modules**	**Diabetes management modules**	**Risk assessment**
Track blood glucose;	Track	Monitoring	Low risk
Graphs;	Structured display	-	Low risk
Customised reminders to monitor;	Personalized feedback	Monitoring	Low risk

Diabetes App

**Functions**	**Functional modules**	**Diabetes management modules**	**Risk assessment**
Track blood glucose;	Track	Monitoring	Low risk
Track food;	Track	Lifestyle modification	Low risk
Graphs;	Structured display	-	Low risk

MiniMed Connect

Functions	Functional modules	Diabetes management modules	Risk assessment
Track blood glucose;	Track	Monitoring	Low risk
Track insulin;	Track	Medications	Potential risk
Graphs;	Structured display	-	Low risk
MiniMed Connect links your MiniMed pump and continuous glucose monitoring (CGM) sensor.			

Lazysugar

Functions	Functional modules	Diabetes management modules	Risk assessment
Track blood glucose;	Track	Monitoring	Low risk
Feedback and rewards of checking;	Personalized feedback	Monitoring	Low risk

Diabetes

Functions	Functional modules	Diabetes management modules	Risk assessment
Track blood glucose;	Track	Monitoring	Low risk
Trends and graphs;<br>Tags;	Structured display	-	Low risk

Gluco Logger

Functions	Functional modules	Diabetes management modules	Risk assessment
Track blood glucose;	Track	Monitoring	Low risk
Trends;	Structured display	-	Low risk

Gmate SMART

Functions	Functional modules	Diabetes management modules	Risk assessment
Track blood glucose;	Track	Monitoring	Low risk
Graphs;	Structured display	-	Low risk

Glucose Recorder/Free

Functions	Functional modules	Diabetes management modules	Risk assessment
Track blood glucose;	Track	Monitoring	Low risk
Graphs;	Structured display	-	Low risk

Diabetes Importer by mySugr

Functions	Functional modules	Diabetes management modules	Risk assessment
Track blood glucose;	Track	Monitoring	Low risk
Graphs;	Structured display	-	Low risk

References

1. de RM, Kim J, Jing Y, Khadra M, Nanan R. A systematic review on incentive-driven mobile health technology: As used in diabetes management. J Telemed Telecare 2016 Feb 16. PMID:26888421
2. Basilico A, Marceglia S, Bonacina S, Pinciroli F. Advising patients on selecting trustful apps for diabetes self-care. Comput Biol Med 2016 Apr 01; 71:86-96. PMID:26897071
3. Riazi H, Larijani B, Langarizadeh M, Shahmoradi L. Managing diabetes mellitus using information technology: a systematic review. J Diabetes Metab Disord 2015; 14:49. PMID:26075190
4. Williams JP, Schroeder D. Popular Glucose Tracking Apps and Use of mHealth by Latinos With Diabetes: Review. JMIR Mhealth Uhealth 2015; 3(3):e84. PMID:26307533
5. Lewis TL, Wyatt JC. mHealth and mobile medical Apps: a framework to assess risk and promote safer use. J Med Internet Res 2014 Sept 15; 16(9):e210. PMID:25223398
6. El-Gayar O, Timsina P, Nawar N, Eid W. Mobile applications for diabetes self-management: status and potential. J Diabetes Sci Technol 2013; 7(1):247-62. PMID:23439183
7. El-Gayar O, Timsina P, Nawar N, Eid W. A systematic review of IT for diabetes self-management: are we there yet?. Int J Med Inform 2013 Aug; 82(8):637-52. PMID:23792137
8. Årsand E, Frøisland DH, Skrøvseth SO, Chomutare T, Tatara N, Hartvigsen G, Tufano JT. Mobile health applications to assist patients with diabetes: lessons learned and design implications. J Diabetes Sci Technol 2012 Sept; 6(5):1197-206. PMID:23063047
9. Demidowich AP, Lu K, Tamler R, Bloomgarden Z. An evaluation of diabetes self-management applications for Android smartphones. J Telemed Telecare 2012 Jun; 18(4):235-8. PMID:22604278
10. Holtz B, Lauckner C. Diabetes management via mobile phones: a systematic review. Telemed J E Health 2012 Apr; 18(3):175-84. PMID:22356525
11. Liang X, Wang Q, Yang X, Cao J, Chen J, Mo X, Huang J, Wang L, Gu D. Effect of mobile phone intervention for diabetes on glycaemic control: a meta-analysis. Diabet Med 2011 Apr; 28(4):455-63. PMID:21392066
12. Rao A, Hou P, Golnik T, Flaherty J, Vu S. Evolution of data management tools for managing self-monitoring of blood glucose results: a survey of iPhone applications. J Diabetes Sci Technol 2010 Jul; 4(4):949-57. PMID:20663461
13. American Diabetes Association. Standards of medical care in diabetes-2017. Diabetes Care 2017 Jan;40(Suppl 1):S1-S132. URL: http://professional.diabetes.org/sites/professional.diabetes.org/files/media/dc_40_s1_final.pdf
14. Food and Drug Administration, Federal Communications Commission, The Office of the National Coordinator for Health Information Technology. 2014 Apr. FDASIA health IT report: proposed strategy and recommendations for a risk-based framework. URL:http://www.fda.gov/downloads/AboutFDA/CentersOffices/OfficeofMedicalProductsandTobacco/CDRH/CDRHReports/UCM391521.pdf
