# Supplementary material for: Mobile App-Based Interventions to Support Diabetes Self-Management: A Systematic Review of Randomized Controlled Trials to Identify Functions Associated with Glycemic Efficacy
Source: JMIR Mhealth Uhealth. 2017 Mar 14;5(3):e35. doi: 10.2196/mhealth.6522 (PMC5373677; doi:10.2196/mhealth.6522)
Supplement: Multimedia Appendix 2 [file mhealth_v5i3e35_app2.pdf]

Multimedia Appendix 2 MEDLINE search strategy

Item#	Search terms
**1**	Search “diabetes mellitus”[mh] OR diabete*[tw] OR diabetic*[tw] OR IDDM[tw] OR NIDDM[tw] OR T1DM[tw] OR T2DM[tw] OR T1D[tw] OR T2D[tw] OR DM[tw] OR MODY[tw]OR ((Insulin*[tw] OR noninsulin*[tw]) AND depend*[tw])
**2**	Search “blood glucose”[mh] OR ((“blood”[mh] OR “blood”[subheading] OR blood[tw] OR serum[tw] OR plasma[tw]) AND (glucose[tw] OR sugar[tw])) OR glycemi*[tw] OR glycemi*[tw]
**3**	Search “blood glucose self-monitoring”[mh]
**4**	Search #1 or #2 or #3
**5**	Search “mobile applications”[mh] OR app [tiab] OR apps [tiab] OR ((application[tiab] OR applications[tiab] OR software[tiab) AND mobile[tw])
**6**	Search “cell phones”[mh] OR phone*[tw] OR cellphone*[tw] OR smartphone*[tw] OR iphone*[tw] OR ipad*[tw] OR ipod*[tw] OR ios[tw] OR android[tw]
**7**	Search #5 or #6
**8**	Search #4 and #7
**9**[a]	Search "2007"[Date-Publication]:"3000"[Date-Publication]
**10**	Search #8 and #9

[a]The universal operating systems of mobile devices were marketed in 2007.
