# Supplementary material for: Mobile App-Based Interventions to Support Diabetes Self-Management: A Systematic Review of Randomized Controlled Trials to Identify Functions Associated with Glycemic Efficacy
Source: JMIR Mhealth Uhealth. 2017 Mar 14;5(3):e35. doi: 10.2196/mhealth.6522 (PMC5373677; doi:10.2196/mhealth.6522)
Supplement: Multimedia Appendix 3 [file mhealth_v5i3e35_app3.pdf]

Multimedia Appendix 4 List of excluded references with reasons for exclusions

No.		Reason for exclusion
**1**	Or C, Tao D. A 3-Month Randomized Controlled Pilot Trial of a Patient-Centered, Computer-Based Self-Monitoring System for the Care of Type 2 Diabetes Mellitus and Hypertension. *J Med Syst* 2016; **40**: 81.	Included participants with hypertension or diabetes
**2**	Kim JM, Lee HJ, Kim KO, Won JC, Ko KS, Rhee BD. Clinical Evaluation of OneTouch Diabetes Management Software System in Patients with Type 2 Diabetes Mellitus. Diabetes Metab J 2016; 40: 129–39.	Not a app-based mobile health intervention
**3**	Siavash M, Taherian M, Khorasgani MA. Efficacy of bolus insulin calculation by a mobile-based bolus advisor: An open label clinical trial. *J Res Med Sci* 2015; **20**: 1064-9.	Not a randomized controlled trial
**4**	Van Olmen J, Van Pelt M, Malombo B, et al. Process evaluation of a mobile health intervention for people with diabetes in low income countries - the implementation of the TEXT4DSM study. *J Telemed Telecare* 2015: 1357633X15617885.	Without outcomes of interests
**5**	Levy N, Moynihan V, Nilo A, et al. The Mobile Insulin Titration Intervention (MITI) for Insulin Adjustment in an Urban, Low-Income Population: Randomized Controlled Trial. *J Med Internet Res* 2015; **17**: e180.	An intervention without instant interaction
**6**	Fountoulakis S, Papanastasiou L, Gryparis A, Markou A, Piaditis G. Impact and duration effect of telemonitoring on EtabA1c, BMI and cost in insulin-treated Diabetes Mellitus patients with inadequate glycemic control: A randomized controlled study. *Hormones (Athens)* 2015;	A telemonitoring system without instant interaction
**7**	Patnaik L, Joshi A, Sahu T. Mobile phone-based education and counseling to reduce stress among patients with diabetes mellitus attending a tertiary care hospital of India. *Int J Prev Med* 2015; **6**: 37.	Without outcomes of interests
**8**	Karhula T, Vuorinen AL, Raapysjarvi K, et al. Telemonitoring and Mobile Phone-Based Health Coaching Among Finnish Diabetic and Heart Disease Patients: Randomized Controlled Trial. *J Med Internet Res* 2015; **17**: e153.	Without instant interaction
**9**	Skrovseth SO, Arsand E, Godtliebsen F, et al. Data-Driven Personalized Feedback to Patients with Type 1 Diabetes: A Randomized Trial. *Diabetes Technol Ther* 2015; **17**: 482–89;	Control group received an mobile health application without CDSS
**10**	Shahid M, Mahar SA, Shaikh S, et al. Mobile phone intervention to improve diabetes care in rural areas of Pakistan: a randomized controlled trial. *J Coll Physicians Surgeons Pakistan* 2015; **25**: 166–71.	Use telephone only without instant interaction
**11**	Pizzi LT, Zangalli CS, Murchison AP, et al. Prospective randomized controlled trial comparing the outcomes and costs of two eyecare adherence interventions in diabetes patients. *Appl Health Econ Health Policy* 2015; **13**: 253–63	Without outcomes of interests
**12**	Nobis S, Lehr D, Ebert DD, et al. Efficacy of a web-based intervention with mobile phone support in treating depressive symptoms in adults with type 1 and type 2 diabetes: a randomized controlled trial. *Diabetes Care* 2015; **38**: 776–83.	Without outcomes of interests
**13**	Kim KM, Park KS, Lee HJ, et al. Efficacy of a New Medical Information system, Ubiquitous	Not a app-based mobile health

	Healthcare Service with Voice Inception Technique in Elderly Diabetic Patients. Sci Rep 2015; 5: 18214.	intervention
**14**	Lim S, Kang SM, Kim KM, et al. Multifactorial intervention in diabetes care using real-time monitoring and tailored feedback in type 2 diabetes. Acta Diabetol 2015; 1–10.	Not a app-based mobile health intervention
**15**	Katalenich B, Shi L, Liu S, et al. Evaluation of a Remote Monitoring System for Diabetes Control. Clin Ther 2015; 37: 1216–25.	Not a app-based mobile health intervention
**16**	Quinn CC, Sareh PL, Shardell ML, Terrin ML, Barr EA, Gruber-Baldini AL. Mobile Diabetes Intervention for Glycemic Control: Impact on Physician Prescribing. *J Diabetes Sci Technol* 2014; **8**: 362-70.	A cluster randomized trial
**17**	Kim HS, Choi W, Baek EK, et al. Efficacy of the smartphone-based glucose management application stratified by user satisfaction. *Diabetes Metab J* 2014; **38**: 204-10.	Not a randomized trial
**18**	Arora S, Burner E, Lam J, De Santos R, Meeks A, Menchine M. Trial to examine text-message based mhealth in ed patients with diabetes (TExT-MED). *Acad Emerg Med* 2014; **63:** 745–54.	Use SMS only without instant interaction
**19**	Vervloet M, van Dijk L, de Bakker DH, et al. Short- and long-term effects of real-time medication monitoring with short message service (SMS) reminders for missed doses on the refill adherence of people with Type 2 diabetes: evidence from a randomized controlled trial. *Diabet Med* 2014; **31**: 821–28.	Using SMS only without instant interaction
**20**	Arora S, Peters AL, Burner E, et al. Trial to examine text message-based mHealth in emergency department patients with diabetes (TExT-MED): a randomized controlled trial. *Ann Emerg Med* 2014; **63**: 745–54.	Use SMS only without instant interaction
**21**	Ruiz JG, Andrade AD, Anam R, Lisigurski M, Karanam C, Sharit J. Computer-based programmed instruction did not improve the knowledge retention of medication instructions of individuals with type 2 diabetes mellitus. *Diabetes Educ* 2014; **40**: 77-88.	Without outcomes of interests
**22**	Tang PC, Overhage JM, Chan AS, et al. Online disease management of diabetes: engaging and motivating patients online with enhanced resources-diabetes (EMPOWER-D), a randomized controlled trial. *J Am Med Inform Assoc* 2013; **20**: 526-34.	An integrated personal health records (PHRs) system for healthcare providers with no data available on users' smartphones
**23**	Nagrebetsky A, Larsen M, Craven A, et al. Stepwise self-titration of oral glucose-lowering medication using a mobile telephone-based telehealth platform in type 2 diabetes: a feasibility trial in primary care. *J Diabetes Sci Technol* 2013; **7**: 123-34.	Without instant interaction
**24**	Brath H, Morak J, Kastenbauer T, et al. Mobile health (mHealth) based medication adherence measurement - a pilot trial using electronic blisters in diabetes patients. *Br J Clin Pharmacol* 2013; **76 Suppl 1**: 47-55.	Without outcomes of interests
**25**	Wongrochananan S, Jiamjarasrangsi W, Tuicomepee A, et al. The effectiveness of interactive multi-modality intervention on self-management support of type 2 diabetic patients in Thailand: A cluster randomized controlled trial. *J Diabetes* 2013; **5**: 151–52.	Use SMS only without instant interaction.
**26**	Orsama AL, Lahteenmaki J, Harno K, Kulju M, Wintergerst E, Schachner H, Stenger P, Leppanen J, Kaijanranta H, Salaspuro V, Fisher WA. Active assistance technology reduces glycosylated hemoglobin and weight in individuals with type 2 diabetes: results of a theory-based randomized trial. Diabetes Technol Ther 2013; 15: 662-9.	Feedback is more often provided in asynchronous and intermittent format than in real time.
**27**	Williams ED, Bird D, Forbes AW, et al. Randomized controlled trial of an automated, interactive	Use telephone only without instant

	telephone intervention (TLC Diabetes) to improve type 2 diabetes management: baseline findings and six-month outcomes. *BMC Public Health* 2012; **12**: 602.	interaction
**28**	Del Prato S, Nicolucci A, Lovagnini-Scher AC, Turco S, Leotta S, Vespasiani G. Telecare Provides comparable efficacy to conventional self-monitored blood glucose in patients with type 2 diabetes titrating one injection of insulin glulisine-the ELEONOR study. *Diabetes Technol Ther* 2012; **14**: 175-82.	An intervention for healthcare provides without instant interaction
**29**	Vervloet M, van Dijk L, Santen-Reestman J, et al. SMS reminders improve adherence to oral medication in type 2 diabetes patients who are real time electronically monitored. *Int J Med Inform* 2012; **81**: 594-604.	Use SMS only without instant interaction.
**30**	Logan AG, Irvine MJ, McIsaac WJ, et al. Effect of home blood pressure telemonitoring with self-care support on uncontrolled systolic hypertension in diabetics. *Hypertension* 2012; **60**: 51–57.	Without outcomes of interests
**31**	Goodarzi M, Ebrahimzadeh I, Rabi A, et al. Impact of distance education via mobile phone text messaging on knowledge, attitude, practice and self efficacy of patients with type 2 diabetes mellitus in Iran. *J Diabetes Metab Disord* 2012; **11**: 1–8.	Use SMS only without instant interaction
**32**	Bell AM, Fonda SJ, Walker MS, et al. Mobile phone-based video messages for diabetes self-care support. *J Diabetes Sci Technol* 2012; **6**: 310–19.	Use video SMS only without instant interaction
**33**	O'Grady MJ, Retterath AJ, Keenan DB, et al. The use of an automated, portable glucose control system for overnight glucose control in adolescents and young adults with type 1 diabetes. *Diabetes Care* 2012; **35**: 2182-7.	A matched insulin app
**34**	Weinstock RS, Teresi JA, Goland R, et al. Glycemic control and health disparities in older ethnically diverse underserved adults with diabetes: five-year results from the Informatics for Diabetes Education and Telemedicine (IDEATel) study. *Diabetes Care* 2011; **34**: 274-9.	A telecare system without instant interaction
**35**	Castelnuovo G, Manzoni GM, Cuzziol P, et al. TECNOB Study: Ad Interim Results of a Randomized Controlled Trial of a Multidisciplinary Telecare Intervention for Obese Patients with Type-2 Diabetes. *Clin Pract Epidemiol Ment Health* 2011; **7**: 44-50.	A web-site based intervention without instant interaction on mobile phone
**36**	Lim S, Kang SM, Shin H, et al. Improved glycemic control without hypoglycemia in elderly diabetic patients using the ubiquitous healthcare service, a new medical information system. Diabetes Care 2011; 34: 308–13.	Not a app-based mobile health intervention
**37**	Stone RA, Rao RH, Sevick MA, et al. Active care management supported by home telemonitoring in veterans with type 2 diabetes: the DiaTel randomized controlled trial. *Diabetes Care* 2010; **33**: 478-84.	A telecare system without instant interaction
**38**	Davis RM, Hitch AD, Salaam MM, Herman WH, Zimmer-Galler IE, Mayer-Davis EJ. TeleHealth improves diabetes self-management in an underserved community: diabetes TeleCare. *Diabetes Care* 2010; **33**: 1712-7.	A telecare system without instant interaction
**39**	Noh JH, Cho YJ, Nam HW, et al. Web-based comprehensive information system for self-management of diabetes mellitus. *Diabetes Technol Ther* 2010; **12**: 333–7.	An educational program to users without instant interaction
**40**	Earle KA, Istepanian RS, Zitouni K, et al. Mobile telemonitoring for achieving tighter targets of blood pressure control in patients with complicated diabetes: a pilot study. *Diabetes Technol Ther* 2010; **12**: 575–79.	Without outcomes of interests
**41**	Kim CS, Park SY, Kang JG, et al. Insulin dose titration system in diabetes patients using a short messaging service automatically produced by a knowledge matrix. Diabetes Technol Ther 2010; 12: 663–69.	Not a app-based mobile health intervention

**42**	Cho JH, Lee HC, Lim DJ, Kwon HS, Yoon KH. Mobile communication using a mobile phone with a glucometer for glucose control in Type 2 patients with diabetes: as effective as an Internet-based glucose monitoring system. *J Telemed Telecare* 2009; **15**: 77-82.	Without standard care control group
**43**	Holbrook A, Thabane L, Keshavjee K, et al. Individualized electronic decision support and reminders to improve diabetes care in the community: COMPETE II randomized trial. *CMAJ* 2009; **181**: 37–44.	Cluster randomized control trial
**44**	Hanauer DA, Wentzell K, Laffel N, et al. Computerized Automated Reminder Diabetes System (CARDS): e-mail and SMS cell phone text messaging reminders to support diabetes management. *Diabetes Technol Ther* 2009; **11**: 99–106.	Control group received e-mail reminders
**45**	Rodriguez-Idigoras MI, Sepulveda-Munoz J, Sanchez-Garrido-Escudero R, Martinez-Gonzalez JL, Escolar-Castello JL, Paniagua-Gomez IM, Bernal-Lopez R, Fuentes-Simon MV, Garofano-Serrano D. Telemedicine influence on the follow-up of type 2 diabetes patients. Diabetes Technol Ther 2009; 11: 431-7.	Not an app based-intervention
**46**	Handley MA, Shumway M, Schillinger D. Cost-effectiveness of automated telephone self-management support with nurse care management among patients with diabetes. *Ann Fam Med* 2008; **6**: 512-8.	Without outcomes of interests
**47**	Yoon KH, Kim HS. A short message service by cellular phone in type 2 diabetic patients for 12 months. *Diabetes Res Clin Pract* 2008; **79**: 256–61.	Use SMS only without instant interaction
**48**	Kim SI, Kim HS. Effectiveness of mobile and internet intervention in patients with obese type 2 diabetes. *Int J Med Inform* 2008; **77**: 399–404.	Use SMS only without instant interaction
**49**	Kim HS, Song MS. Technological intervention for obese patients with type 2 diabetes. *Appl Nurs Res* 2008; **21**: 84–89.	An educational intervention without instant interaction
**50**	Faridi Z, Liberti L, Shuval K, Northrup V, Ali A, Katz DL. Evaluating the impact of mobile telephone technology on type 2 diabetic patients' self-management: the NICHE pilot study. J Eval Clin Pract 2008; 14: 465–69.	Not real-time intervention (daily messages).
**51**	Kim HS. A randomized controlled trial of a nurse short-message service by cellular phone for people with diabetes. *Int J Nurs Stud* 2007; **44**: 687-92.	Use SMS only without instant interaction
**52**	Benhamou PY, Melki V, Boizel R, et al. One-year efficacy and safety of Web-based follow-up using cellular phone in type 1 diabetic patients under insulin pump therapy: the PumpNet study. *Diabetes Metab* 2007; **33**: 220–26.	A matched CSII app
**53**	Luzio S, Piehlmeier W, Tovar C, et al. Results of the pilot study of DIADEM: a comprehensive disease management programme for type 2 diabetes. *Diabetes Res Clin Pract* 2007; **76**: 410-7.	Not a randomized trial
**54**	Logan AG, McIsaac WJ, Tisler A, et al. Mobile phone-based remote patient monitoring system for management of hypertension in diabetic patients. *Am J Hypertens* 2007; **20**: 942-8.	Without outcomes of interests
**55**	Izquierdo R, Meyer S, Starren J, et al. Detection and remediation of medically urgent situations using telemedicine case management for older patients with diabetes mellitus. *Ther Clin Risk Manag* 2007; **3**: 485-9.	Without outcomes of interests
