# Supplementary material for: Mobile App-Based Interventions to Support Diabetes Self-Management: A Systematic Review of Randomized Controlled Trials to Identify Functions Associated with Glycemic Efficacy
Source: JMIR Mhealth Uhealth. 2017 Mar 14;5(3):e35. doi: 10.2196/mhealth.6522 (PMC5373677; doi:10.2196/mhealth.6522)
Supplement: Multimedia Appendix 4 [file mhealth_v5i3e35_app4.pdf]

Multimedia Appendix 5 The detailed information, taxonomic classification and risk of bias of included trials

Hsu 2016

**Methods**	Study design: a randomized controlled study.<br>Duration of study: 12 weeks.<br>Run-in time: not reported.<br>Clinic visit: 0, 3 months.<br>Setting: a tertiary diabetes center of the Massachusetts Institute of Technology (MIT) and the Joslin Diabetes Center.<br>Country: USA
**Participants**	Identify: a tertiary diabetes center with care provided by teams of endocrinologists, nurse practitioners, and certified diabetes educators.<br>Inclusion criteria: Subjects with type 2 diabetes (above 18 years of age with HbA1c levels of 9–14%) who were being started on basal insulin therapy by their treating HCPs and had internet connectivity were eligible for inclusion in the study.<br>Exclusion criteria: Subjects with significant visual or hearing impairment, who were not proficient in English, who were pregnant or lactating, who had alcohol dependency, or who required multiple daily insulin injections were excluded.<br>Number of subjects: I: baseline-20, end-15, C: baseline-20, end-16;<br>Race: not reported.<br>Education level: not reported.
**Interventions**	Intervention group-the cloud-based diabetes management program<br>Regular communications about glycemic control and insulin doses were conducted via patient self-tracking tools, shared decision-making interfaces, secure text messages, and virtual visits instead of office visits. The plan can include any number of medications a day, which can be scheduled at specific times with flexible adherence windows. The plan is visualized for the patient on the tablet computer application in order to provide daily awareness and to allow self-tracking of medication adherence and blood glucose. (A wireless glucose meter [model D40b; ForaCare, Moorpark, CA] is integrated into the program and automates the reporting of blood glucose.) The interface emphasizes that other factors, such as medication adherence and diet and exercise, should be accounted for in the decision. The streamlined communication tools integrated into the application help facilitate timely learning and clinical support based on trends in data and decision-making events. The tablet computer simply visualizes the data to make it easier for the subject to make an informed decision. No instance will the computer make insulin titration decisions. Each hypoglycemic reading was also electronically tracked, along with the subject's response as to whether symptoms of hypoglycemia were experienced and what subsequent actions were taken. The diabetes management program was developed using the CollaboRhythm software platform designed at the MIT Media Lab, Cambridge, MA. The streamlined communications tools (secure text messages and virtual visits) were integrated into the application. The shared decision-making interfaces include weekly charts to help the subjects and HCPs.<br>Control group-standard face-to-face care<br>Subjects in the control group received standard care at the clinic in initiating and titrating insulin, with interim face- to-face visits, as well as telephone/fax communication with educators and physicians as dictated by their HCPs. Rates of hypoglycemia and the frequency of communications were obtained by re-viewing the subjects' medical records.
**Outcomes**	Primary outcome: the absolute HbA1c level change in 3 months;<br>Second outcome: the percentage reaching the glycemic target of A1c ≤7%, the change between patient satisfaction before and after the study, the frequency for hypoglycemia, and the time HCPs and subjects spent on managing the insulin titration;

	Outcomes of interest:<br>HbA1c%:<br>Intervention group: baseline 10.9±1.2, 3 month 7.7±1.6, change -3.2±1.5<br>Control group: baseline 10.8±1.2, 3 month 8.9±2.2, change -2±2<br>Adverse events:<br>0-3 month:<br>Hypoglycemia: Intervention group: 4 subjects, Control group: 2 subjects.<br>No one required outside assistance in treating hypoglycemia. However, we were only able to obtain hypoglycemic complaints in the control group from subjects who either called following an episode or reported hypoglycemia at the end visit, in contrast to digitally capturing hypoglycemic glucose readings from the intervention group.
**Publication details**	Language: English<br>Funding: none<br>Publication statues: peer reviewed journal

**Functions**		**Diabetes management modules**				
		**Monitoring**	**Medication management**	**Lifestyle modification**	**Complication prevention**	**Psychosocial care**
**Functional modules**	**Log**	Recording blood glucose;	Recording medications and insulin;	Recording diet and exercise;	Recording symptoms of hypoglycemia	-
	**Structured display**	Visualization;				
	**General education**	-	-	Lifestyle education and emphasizes;	-	-
	**Personalized feedback**	-	-	-	-	-
	**Communication**	Communication with HCPs through secure text messages and virtual visits weekly				

**Risk of bias**		
**Domain**	**Review authors' judgement**	**Support for judgement**
Random sequence generation (selection bias)	Unclear risk	Quote: "Twenty subjects were randomized to the intervention group, versus 20 to the control group."<br>Comment: insufficient information provided.
Allocation concealment (selection bias)	Unclear risk	Quote: "Twenty subjects were randomized to the intervention group, versus 20 to the control group."<br>Comment: insufficient information provided.
Blinding of outcome assessment (detection bias, HbA1c)	Low risk	HbA1c is an objective measurement which is not likely to be influenced by whether or not assessors are blinded.
Blinding of outcome assessment (detection bias, adverse events)	Low risk	Hypoglycemia rate was obtained by reviewing the medical records, which is not likely to be influenced by whether or not assessors are blinded.
Incomplete outcome data addressed (attrition bias)<br>(HbA1c and adverse events)	Low risk	6 month: Five subjects (one from the intervention group and four from the control group) dropped out from the study. Specifically, three failed to show up at the final visit (one from the intervention

		group and two from the control group), and two opted to participate in a medically supervised weight loss program, which was not part of the study protocol.<br>Comment: missing outcome data balanced in numbers across intervention groups, with similar reasons for missing data across groups.
Selective reporting<br>(reporting bias)	Low risk	Comment: the study protocol is available and all of the study's pre-specified (primary and secondary) outcomes that are of interest in the review have been reported in the pre-specified way.
Other sources of bias	Unclear risk	Comment: Small convenience sample.

Baron 2016

**Methods**	Study design: a randomized controlled trial.<br>Duration of study: 9 months.<br>Run-in time: not reported.<br>Clinic visit: 0, 3, 9 months.<br>Setting: a diabetes clinic in east London.<br>Country: United Kingdom
**Participants**	Identify: Participants with an appointment in the following two weeks were screened for eligibility and sent recruitment materials.<br>Inclusion criteria: age 18 or above, poorly controlled type 1 or type 2 diabetes (HbA1c 5 7.5%) with the latest HbA1c collected within the last 12 months, taking insulin, and fluency and literacy in English.<br>Exclusion criteria: previous experience using MTH, regular extended travels (53 weeks) outside the UK, home visits by a district nurse for BG monitoring and/or insulin administration, a diagnosis of kidney failure or sickle cell disease, pregnancy, and dexterity/visual problems compromising the use of a mobile-phone.<br>Number of subjects (diabetes group): I: baseline-45, end-40, C: baseline-36, end-31;<br>Race: white 20 (24.5%), black 27 (33.3%), Asian 29 (35.8%), other 5 (6.2%).<br>Education level: no formal education 26 (32.1%), GCSE/O' levels 27 (33.3%), A-level/HNC 9 (11.1%), university level 10 (12.3%), graduate/professional 9 (11.1%).
**Interventions**	Intervention group-standard care supplemented with mobile telehealth (MTH)<br>Self-monitoring, mobile-phone data transmissions, graphical and nurse-initiated feedback, and educational calls. The MTH equipment included BG meter, BP monitor, mobile-phone, and Bluetooth cradle and the mobile-phone software allowed participants to store and transmit diabetes-related data (BG and BP readings, time since last meal, level of physical activity performed that day, insulin dose, and weight) to an MTH nurse. Colour-coded graphical feedback on the data recorded could be accessed through the mobile-phone menu, and was automatically displayed following each data transfer. In addition to providing feedback on out-of-range clinical readings (as needed) and education on lifestyle changes, the MTH nurses supported insulin titration.<br>Control group-standard care<br>Standard care at the diabetes clinic consisted of follow-up appointments with a DSN every three to four months, and one annual or two semi-annual appointments with diabetes consultants, depending on glycemic control.
**Outcomes**	Primary outcome: HbA1c;<br>Second outcome: BP and daily insulin dose, and number of DOAs attended with a DSN or consultant.<br>Outcomes of interest:<br>HbA1c%:<br>Intervention group: baseline 9.07±1.72, 3 month 8.76±1.70, 9 month 8.56±1.64<br>Control group: baseline 8.88±1.68, 3 month 8.82±1.68, 9 month 8.93±1.61

	Adverse events: not report
**Publication details**	Language: English.<br>Funding: the Policy Research Programme of the Department of Health for England.<br>Publication statues: peer reviewed journal.

**Functions**		**Diabetes management modules**				
		**Monitoring**	**Medication management**	**Lifestyle modification**	**Complication prevention**	**Psychosocial care**
**Functional modules**	**Log**	Recording blood glucose, blood pressure and pulse;	Recording insulin dose;	Recording meal time, physical activity, and weight;	-	-
	**Structured display**	Graphs;				
	**General education**	-	-	Education on lifestyle changes;	-	-
	**Personalized feedback**	Off-target alerts;	-	-	-	-
	**Communication**	Connection with MTH nurses through web portal;				

**Risk of bias**		
**Domain**	**Review authors' judgement**	**Support for judgement**
Random sequence generation (selection bias)	Low risk	Quote: "Randomisation was carried out using an online sequence generator"
Allocation concealment (selection bias)	Low risk	Quote: "Randomisation was carried using an online sequence generator that generated randomized block allocations"
Blinding of outcome assessment (detection bias) (HbA1c)	Low risk	HbA1c is an objective measurement which is not likely to be influenced by whether or not assessors are blinded.
Incomplete outcome data addressed (attrition bias)<br>(HbA1c)	Low risk<br>Intervention group: Dropouts (n=3 all within 3 months because inability to use technology in n=2, health problem in n=1*).Lost to follow-up (n=1 at 9 months; questionnaire lost in post). Did not reach 9 month follow-up (n=1, contract constraints, i.e. questionnaire not sent). Deceased (n=1, within 3 months).<br>Control group: Dropouts (n=1 before 3 months, lack of time). Lost to follow-up (n=2 at 3	

	months because of lack of time, and illness).Did not reach 9 month follow-up (n=3, contract constraints, i.e. questionnaire not sent out). Deceased (n=1, just before 9 month follow-up). Comment: missing outcome data balanced in numbers across intervention groups, with similar reasons for missing data across groups.	
Selective reporting (reporting bias)	High risk	Comment: the study report fails to include adverse events for a key outcome that would be expected to have been reported for such a study.
Other sources of bias	Low risk	Comment: the study appears to be free of other sources of bias.

Drion 2015

**Methods**	Study design: block randomized controlled trial<br>Duration of study: 3 month<br>Run-in time: not reported.<br>Clinic visit: 0 and 3 months<br>Setting: diabetes outpatient clinic Isala hospital in Zwolle.<br>Country: Netherlands
**Participants**	Identify: patients with T1DM who visit the diabetes outpatient clinic between Sept. until Oct. 2011.<br>Inclusion criteria: over 18 years old, had T1DM, and were treated with insulin.<br>Exclusion criteria: had used a diabetes application in the 3 months prior to their visit, did not have internet or email access, or were unable to read Dutch<br>Number of subjects: I: baseline-31, end-30; C: baseline-32, end-32.<br>Race: not reported<br>Education level: primary school 2 (3.1%), low level 2 (3.1%), intermediate level 20 (31.7%), high school 6 (9.5%), University 33 (52.4%)
**Interventions**	Intervention group- Diabetes Under Control (DBEES) application (on market)<br>A digital diabetes diary which could manually enter diabetes-related self-care data: blood glucose values, carbohydrate intake, medication, physical exercise, and notes into the application.<br>Control group-standard paper diary.
**Outcomes**<br>Primary outcomes: the change in QOL (the RAND-36 questionnaire).<br>Secondary outcomes: diabetes-related distress (PAID questionnaire),	

HbA1c, daily frequency of SMBG, and usability of the DBEES system (SUS questionnaire).<br>Adverse events: not reported.<br>Outcomes of interest:<br>HbA1c%:<br>Intervention group: baseline 7.73 (7.37, 8.09), 3 months 7.91 (7.46, 8.28)<br>Control group: baseline 7.82 (7.37, 8.19), 3 months 7.91 (7.37, 8.46)	
**Publication details**	Language: English<br>Funding: no<br>Publication statues: peer reviewed journal

**Functions**		**Diabetes management modules**				
		**Monitoring**	**Medication management**	**Lifestyle modification**	**Complication prevention**	**Psychosocial care**
**Functional modules**	**Log**	Recording blood glucose levels;	Recording medications;	Recording carbohydrate and physical exercise;	-	-
	**Structured display**	Customized notes;				
	**General education**	-	-	-	-	-
	**Personalized feedback**	-	-	-	-	-
	**Communication**	-				

**Risk of bias**		
**Domain**	**Review authors' judgement**	**Support for judegement**
Random sequence generation (selection bias)	Low risk	Quote: Randomization was performed through a telephone call with an independent researcher who was asked to draw a nontransparent envelope. All envelopes contained tickets with an I (for the intervention group) or a C (for the control group). To ensure equal allocation rates within the 2 groups, block randomization was used."<br>Comment: block randomization was used.
Allocation concealment<br>(selection bias)	Low risk	Quote: Randomization was performed through a telephone call with an independent researcher who was asked to draw a nontransparent

		envelope.<br>Comment: central allocation was used to conceal allocation.
Blinding of outcome assessment (detection bias) (HbA1c)	Low risk	HbA1c is an objective measurement which is not likely to be influenced by whether or not assessors are blinded.
Incomplete outcome data (attrition bias) (HbA1c)	Low risk	1 patient from the intervention group was lost to follow-up.<br>Comment: the proportion of missing outcomes was too low to induce bias in observed effect size.
Selective reporting (reporting bias)	High risk	Comment: the study report fails to include adverse events for a key outcome that would be expected to have been reported for such a study.
Other sources of bias	Low risk	Comment: the study appears to be free of other sources of bias.

Holmen 2014

**Methods**	Study design: 3-arm block randomized controlled trial.<br>Duration of study: 12 months.<br>Run-in time: not reported.<br>Clinic visit: 0,4,12 months<br>Setting: 2 study centers in the southern and northern parts of Norway.<br>Country: Norway.
**Participants**	Identify: eligible patients 2 study centers in the southern and northern parts of Norway in collaboration with their GPs.<br>Inclusion criteria: persons with type 2 diabetes with an HbA1c level ≥7.1% (≥54.1 mmol/mol) and aged ≥18 years, and were capable of completing questionnaires in the Norwegian language, had to be cognitively able to participate and to use the system and devices provided, although prior familiarity with mobile phones was not necessary.<br>Exclusion criteria: not report<br>Number of subjects: I: baseline-50, end-39; AC: baseline-51, end-40; C: baseline-50, end-41.<br>Race: not reported.<br>Education level: <12 years 83 (55.9%), 12 years 17 (11.3%), >12 years 51 (33.8%).
**Interventions**	Intervention group-Few Touch Application (FTA) with health counseling intervention<br>The participants measured blood glucose level with a glucometer (LifeScan OneTouch Ultra Easy), which enabled automatic transfer of the measurement to the diary mobile app and provided visual graphs, trend reports, and feedback through color coding (below normal, normal, and above normal). The phone and the blood glucose meter were linked using Bluetooth wireless communication. The app also consisted of a food habit registration system, a physical activity registration system, a personal goal-setting system, and a general information system. The user entered information about food intake, physical activity, and personal goals manually.<br>Active control group- Few Touch Application (FTA)<br>Control group- usual care by their general practitioner.
**Outcomes**	Primary outcome: HbA1c<br>Secondary outcomes: self-management (heiQ), health-related quality of life (SF-36), depressive symptoms (CES-D), and lifestyle changes (dietary habits and physical activity)<br>Adverse events: not pre-specified<br>Outcomes of interest:<br>HbA1c%<br>Intervention group: baseline-8.2 (1.1), 12 month-8.0 (1.0)<br>Active control group: baseline-8.1 (1.1), 12 month-7.8 (0.9)

	Control group: baseline-8.3 (1.2), 12 month-8.2 (1.1)<br>Adverse events: no adverse clinical events related to the intervention. However, a few undesired technical events were reported, such as trouble with the Bluetooth pairing required for automatic transmission of data from the glucometer to the app in the mobile phone.
**Publication details**	Language: English<br>Funding: (1) the EU through the ICT Policy Support Programme as part of the Competitiveness and Innovation Framework Programme, (2) the Norwegian Research Council, (3) the Health Authorities of Northern Norway, (4) the Norwegian Centre of Integrated Care and Telemedicine at the University Hospital of North-Norway, (5) the Oslo and Akershus University College, (6) the Akershus University Hospital, and (7) the Norwegian Diabetes Association.<br>Publication statues: peer reviewed journal

**Functions**		**Diabetes management modules**				
		**Monitoring**	**Medication management**	**Lifestyle modification**	**Complication prevention**	**Psychosocial care**
**Functional modules**	**Log**	Recording blood glucose levels;	-	Recording food habit and physical activity;	-	-
	**Structured display**	Graphs and trend;				
	**General education**	-	-	A general information system;	-	-
	**Personalized feedback**	Off-targets alerts;<br>A personal goal-setting system;	-	-	-	-
	**Communication**	Health counseling with nurses through 5 telephone calls in 4 months;				

**Risk of bias**		
**Bias**	**Authors' judgement**	**Support for judgement**
Random sequence generation (selection bias)	Low risk	Quote: "randomization is performed through the Center of Randomization at the Unit for Applied Clinical Research at the Norwegian University of Science and Technology in Trondheim, using WebCRF (Case Report Form)."<br>Comment: block randomization was used.
Allocation concealment (selection bias)	Low risk	Quote: "randomization is performed through the Center of Randomization at the Unit for Applied Clinical Research at the Norwegian University of Science and Technology in Trondheim, using WebCRF (Case Report Form)."<br>Comment: central allocation was used to conceal allocation.
Blinding of outcome assessment (detection bias, HbA1c)	Low risk	HbA1c is an objective measurement which is not likely to be influenced by whether or not assessors are blinded.
Blinding of outcome assessment (detection bias, adverse events)	Low risk	A few undesired technical events were reported, such as trouble with the Bluetooth pairing required for automatic transmission of data from the glucometer to the app in the mobile phone. The adverse events were not likely to be influenced by whether or not assessors were blinded.
Incomplete outcome data (attrition bias)	High risk	At 12 month, there was a total dropout attrition rate of 21% (31/151),

(HbA1c)		with an equal distribution in the groups. Reasons for missed follow-up were not reported.
Selective reporting (reporting bias)	High risk	Adverse events were not pre-specified.
Other sources of bias	Low risk	Comment: the study appears to be free of other sources of bias.

Waki 2014

**Methods**	Study design: randomized controlled trial<br>Duration of study: 3 months<br>Run-in time: 2 weeks<br>Clinic visit: 2, 0,12 weeks<br>Setting: University of Tokyo Hospital.<br>Country: Japan
**Participants**	Identify: posters at the University of Tokyo Hospital<br>Inclusion criteria: persons with type 2 diabetes, to be able to exercise<br>Exclusion criteria: have any severe complications—serum creatinine below 1.5 mg/dl, or proliferative retinopathy, could not use the system and the devices properly<br>Number of subjects: I: baseline-27, end-24; C: baseline-27, end-25.<br>Race: not reported<br>Education level: not reported
**Interventions**	Intervention group-DiaBetics<br>DiaBetics included a smartphone (NEC, Tokyo, Japan: MEDIAS WP N-06C), NFC-enabled glucometer (Terumo, Tokyo, Japan: MS-FR201B) and Bluetooth-enabled BP monitor (Omron, Kyoto, Japan: HEM-7081-IT), pedometer (Omron HJ-720IT) with adapter (Omron HHX-IT1), and scale (Omron HBF-206IT), all devices paired with a unique communicator that transmitted the readings by wireless network to the DialBetics server.<br>(1) data transmission module, patients' data—blood glucose, blood pressure, body weight, and pedometer counts—are measured at home and sent to the server twice a day right after the patients' measurement, the first 3 upon waking in the morning, then blood glucose, blood pressure, and pedometer readings at bed time;<br>(2) evaluation module, data are automatically evaluated following the Japan Diabetes Society (JDS) guideline's targeted values—optimally, blood glucose below 110 mg/dl before breakfast, below 140 mg/dl at bed time; blood pressure below 130/80 mmHg; and pedometer count above 10,000. DialBetics determines if each reading satisfies guideline requirements, then immediately sends those results to each patient's smartphone. Readings defined as abnormal—blood glucose above 400 mg/dl or below 40 mg/dl, and systolic blood pressure above 220 mmHg—are reported to a doctor as "Dr Call," meaning a physician will check the data and interact with the patient if necessary;<br>(3) communication module, (a) the patient's voice/text messages about meals—main dish of a meal—and exercise that is not counted by a pedometer— the type of exercise and its duration—are sent to the server; (b) message processing, if by voice input, is converted to text and matched with text in the DialBetics database; (c) advice on lifestyle modification, matched to the patient's input about food and exercise, is sent back to each patient immediately after the patient's input;<br>(4) dietary evaluation module, patients' photos of meals are sent to the server; the nutritional value of those meals is calculated by dieticians, then sent back to each patient. This process usually takes 1 or 2 days. This service was partially assisted by IMD, Inc, Tokyo, Japan.<br>Control group: continue their self-care regimen, but they did not receive or use any devices to monitor their health data; they did not record their diet and exercise.

**Outcomes**	Primary outcome: HbA1c<br>Secondary outcomes: fast blood sugar, LDL-C, HDL-C, TG, and BP, usability, compliance<br>Adverse events: not reported.<br>Outcomes of interest:<br>HbA1c%;<br>Intervention group: baseline-7.1±1.0, 3 month-6.7±0.7<br>Control group: baseline-7.0±0.9, 3 month-7.1±1.1
**Publication details**	Language: English<br>Funding: NTT DOCOMO and Japan Society for Promotion of Science Grant-in-Aid for Young Scientist Research (B) 23790559.<br>Publication statues: peer reviewed journal

**Functions**		**Diabetes management modules**				
		**Monitoring**	**Medication management**	**Lifestyle modification**	**Complicatio n prevention**	**Psychosocial care**
**Functional modules**	**Log**	Recording blood glucose and blood pressure;	-	Recording meals, pedometer counts, and weight;	-	-
	**Structured display**	-				
	**General education**	-	-	-	-	-
	**Personalized feedback**	Targets setting for blood glucose and blood pressure;	-	Targets setting for steps; Advice on food and exercise;	-	-
	**Communication**	Connection with the physician through telephone call if necessary;				

**Risk of bias**		
**Domain**	**Review authors' judgement**	**Support for judgement**
Random sequence generation (selection bias)	Unclear risk	Quote: "These 54 were then randomly divided into 2 groups, 27 in the DialBetics group and 27 in the non-DialBetics control group."<br>Comment: Insufficient information provided
Allocation concealment<br>(selection bias)	Unclear risk	Quote: "These 54 were then randomly divided into 2 groups, 27 in the DialBetics group and 27 in the non-DialBetics control group."<br>Comment: Insufficient information provided
Blinding of outcome assessment (detection bias)<br>(HbA1c)	Low risk	Obtained from medical records<br>Comment: review authors do not believe this will introduce bias
Incomplete outcome data (attrition bias)<br>(HbA1c)	High risk	The total dropout attrition rate of 9.1% (49/54), with an equal distribution in the groups. Reasons for missed follow-up were not reported.
Selective reporting<br>(reporting bias)	High risk	Comment: the study report fails to include adverse events for a key outcome that would be expected to have been reported for

		such a study.
Other sources of bias	Unclear risk	Comment: Small convenience sample.

Kirwan 2013

**Methods**	Study design: parallel randomized controlled trial<br>Duration of study: 9 month<br>Run-in time: not reported<br>Clinic visit: 0, 3, 6, 9 months<br>Setting: not reported<br>Country: Australian
**Participants**	Identify: recruited nationally by means of an invitation letter sent to type 1 diabetes patients registered with Diabetes Australia in New South Wales and Queensland, as well as an advertisement in a type 1 diabetes national newsletter (Yada newsletter) emailed to recipients and promotion in an online community forum (Reality Check Forum).<br>Inclusion criteria: (1) aged 18-65 years, (2) diagnosed with type 1 diabetes >6 months, (3) HbA1c >7.5%, (4) treated with multiple daily injections or insulin pump, and (5) own a smartphone (iPhone).<br>Exclusion criteria: pregnant or already using a smartphone application to self-manage their diabetes.<br>Number of subjects: I: baseline-36, 6 month-28; C: baseline-36, 6 month-32.<br>Race: not reported<br>Education level: not reported
**Interventions**	Intervention group-Glucose Buddy<br>Manually enter blood glucose levels, insulin dosages, other medications, diet (food item in grams), and physical activities (minutes). View their data on a customizable graph.<br>Control group-usual care<br>Continue with their usual care, which included a visit to their primary diabetes health care practitioner every 3 months.
**Outcomes**	Primary outcome: HbA1c<br>Secondary outcomes: diabetes-related self-efficacy (DES-SF), self-care activities (SDSCA), and quality of life (DQOL)<br>Adverse events: not reported.<br>HbA1c%<br>Intervention group: baseline-9.08±1.18, 6 month-7.97±0.73<br>Control group: baseline-8.47±0.86, 6 month-8.43±1.00
**Publication details**	Language: English<br>Funding: Central Queensland University, Australia. The authors thank Certified Diabetes Educator Veronica Mills (Queensland Health) and SkyHealth, the developers of Glucose Buddy application and website.<br>Publication statues: peer reviewed journal

**Functions**		**Diabetes management modules**				
		**Monitoring**	**Medication management**	**Lifestyle modification**	**Complication prevention**	**Psychosocial care**
**Functional modules**	**Track**	Recording blood glucose;	Recording insulin dosages and medications;	Recording diet and activities;	-	-
	**Structured display**	Graph;				
	**General education**	-	-	-	-	-

	**Personalized feedback**	-	-	-	-	-
	**Communication**	-				

**Risk of bias**		
**Domain**	**Review authors' judgement**	**Support for judgement**
Random sequence generation (selection bias)	Low risk	Quote: "a permuted block randomization design method was used during the 3-month rolling recruitment to ensure roughly equal numbers of patients were allocated to each comparison group." Comment: block randomization was used.
Allocation concealment (selection bias)	Low risk	The study coordinator randomized patients using a freely available online randomization program. Comment: central allocation was used to conceal allocation.
Blinding of outcome assessment (detection bias) (HbA1c)	Low risk	HbA1c is an objective measurement which is not likely to be influenced by whether or not assessors are blinded.
Incomplete outcome data (attrition bias) (HbA1c)	High risk	Comment: The dropout was 26% (11 males, 8 females, 19/72). Missing outcome data in numbers across intervention and control groups and reasons for missing data were not reported.
Selective reporting (reporting bias)	High risk	Comment: the study report fails to include adverse events for a key outcome that would be expected to have been reported for such a study.
Other sources of bias	Unclear risk	Funded by the developers of Glucose Buddy application and website.

Rossi 2013

**Methods**	Study design: multicenter parallel randomized clinical trial<br>Duration of study: 6 month<br>Run-in time: 15 days<br>Clinic visit: 0, 3, 6 months<br>Setting-12 diabetes clinics, Valerio Miselli, Elisa Rabitti, Susanna Valenti, Paola Accorsi, and Cristina Dotti, Ospedale Magati, Scandiano (RE); Roberto Anichini and Laura Tedeschi, Ospedale del Ceppo, Pistoia; Paolo Di Bartolo, Cipriana Sardu, Francesca Pellicano, Sara Brandolini, and Patrizia Scolozzi, AUSL Provincia di Ravenna, Ravenna; Concetta Suraci, Santina Abbruzzese, Sergio Leotta, Lucia Fontana, Silvia Carletti, and Maria Altomare, Ospedale Sandro Pertini, Rome; Gabriella Galimberti and Andrea Laurenzi, Istituto Scientifico San Raffaele, Milan; Cristina Trojani and Matteo Bruglia, Ospedale Infermi, Rimini; Luigi Sciangula, Alessandra Ciucci, Elisa Bellini, and Adele Tono, Az. Ospedale S. Anna P.O Cantu` , Mariano Comense (CO); Silvia Acquati, Ospedale G.B. Mor- gagni, L. Pierantoni, Forl`ı; Andrea Matteo Bonomo and Elena Meneghini, Ospedale Niguarda Ca` Granda, Milan; Stefano Del Prato, Alessandra Bertolotto, and Michele Aragona, Ospedale Cisanello, Pisa; Giorgio Grassi and Michela Tomelini, A.O.U. S. Giovanni Battista, Torino; and Mauro Rossi, P.O. di Grosseto Stabilimento Misericordia, Grosseto.<br>Country: Italy
**Participants**	Inclusion criteria: diagnosis of T1DM, ≥18 years of age, no previous education on CHO counting, HbA1c levels ≥7.5%, treatment with a basal-bolus regimen with insulin analogs, SMBG measurements at least three times a day, and adequate familiarity in the use of mobile phones according to the physician judgment.<br>Exclusion criteria: treatment with NPH insulin or soluble regular insulin, continuous subcutaneous insulin infusion, insulin regimens other than basal: bolus, eating disorders (based on the physician's judgment), pregnancy/ lactation, inability to send or receive SMSs, inability or unwillingness to give informed consent, or any other disease or condition that could interfere with the compliance with the

	protocol or the study completion.<br>Number of subjects: I: baseline-63, 6 month-55; C: baseline-64, 6 month-57.<br>Race: not reported<br>Education level: low level (less than college degree) 19 (14.9%), intermediate level (less than university degree) 77 (60.6%), high level (university degree) 31 (24.4%);
**Interventions**	Intervention group-Diabetes Interactive Diary, DID<br>A carbohydrate/insulin bolus calculator, an information technology device, and a telemedicine system based on the communication between a health care professional (physician or dietitian) and a patient via text messages. It supports patients in managing the CHO counting through a food atlas and in recording the self-monitoring blood glucose (SMBG) measurements. On the basis of the stored data (blood glucose values deriving from self-monitoring, individualized correction factor, and insulin: CHO ratio set by the physician, food intake, and physical activities performed), DID suggests the daily carbohydrate intake, and automatically calculates the most appropriate insulin dose to be injected at each meal.<br>Control group-Standard care<br>Patients randomized to the control group received the standard educational approach usually used in the center. The insulin scheme was the same as in Group A. Insulin doses in Group B were adjusted according to the usual practice, on the basis of SMBG values reviewed during the doctor's office visit.
**Outcomes**	Primary outcome: HbA1c<br>Secondary outcomes: fasting blood glucose levels, glucose variability, mean daily doses of basal and prandial insulin, frequency of hypoglycemic episodes, changes in body weight, lipid profile, blood pressure levels, quality of life, patient satisfaction.<br>Adverse events: Grade 1 hypoglycemia was defined as any symptomatic and/or an asymptomatic finger stick plasma glucose of < 3.3 mmol/L (< 60 mg/dL) with the patient not requiring the assistance of other people; grade 2 hypoglycemia was defined as any episode resulting in coma, seizure, or significant neurologic impairment so that the subject was unable to initiate self-treatment or required the assistance of other people.<br>Outcomes of interest:<br>HbA1c%:<br>Intervention group: baseline 8.4±0.1, 6 months 7.9±0.1, change -0.49±0.11<br>Control group: baseline 8.5±0.1, 6 months 8.1±0.1, change -0.48±0.11<br>Adverse events: incidence of grade 1 and grade 2 hypoglycemic episodes<br>Grade 1: intervention group 49.2 (46.7-51.9), standard group 45.6 (43.2-48.1)<br>Grade 2: intervention group 0.33 (0.17-0.63), standard group 2.29 (1.80-2.91)
**Publication details**	Language: English<br>Funding: Sanofi-Aventis SpA, Milan, Italy. Materials for SMBG (glucose meters, strips, lancets, and control solutions) were supplied by LifeScan Inc., Milpitas, CA. Me.Te.Da. s.r.l., San Benedetto del Tronto, Italy, is the software company that developed the DID system.<br>Publication statues: peer reviewed journal

**Functions**		**Diabetes management modules**				
		**Monitoring**	**Medication management**	**Lifestyle modification**	**Complication prevention**	**Psychosocial care**
**Functional modules**	**Log**	Recording blood glucose;	Recording insulin dosages;	Carbohydrate counting;<br>Recoding food intake and physical activities;	-	-
	**Structured display**	-				

	**General education**	-	-	-	-	-
	**Personalized feedback**	-	Calculates insulin dose based on algorithms;	Suggesting the daily carbohydrate intake;	-	-
	**Communication**	In-app communication between patients and physician via text messages.				

**Risk of bias**		
**Domain**	**Review authors' judgement**	**Support for judgement**
Random sequence generation (selection bias)	Low risk	Randomization was performed through a telephone call to the coordinating center. To control for bias deriving from systematical differences in the usual-care approach adopted in the different clinics, random lists were stratified by center.<br>Comment: block randomization was used.
Allocation concealment (selection bias)	Low risk	To ensure equal allocation rates within centers, permuted block randomization has been used.<br>Comment: central allocation was used to conceal allocation.
Blinding of outcome assessment (detection bias, HbA1c)	Low risk	HbA1c is an objective measurement which is not likely to be influenced by whether or not assessors are blinded.
Blinding of outcome assessment (detection bias, adverse events)	Low risk	Grade 1 hypoglycemia was defined as any symptomatic and/or an asymptomatic fingerstick plasma glucose of <3.3 mmol/L (<60 mg/dL) with the patient not requiring the assistance of other people; grade 2 hypoglycemia was defined as any episode resulting in coma, seizure, or significant neurologic impairment so that the subject was unable to initiate self-treatment or required the assistance of other people. These were objective measurements which were not likely to be influenced by whether or not assessors are blinded.
Incomplete outcome data (attrition bias) (HbA1c and adverse events)	Unclear risk	In the intervention group, 2 for drop-out of center, 4 patients unable to continue follow-up, 1 for pregnancy, 1for starting of CSII; In the control group, 2 for drop-out of center, 2 patients unable to continue follow-up, 3 withdrawal of informed consent.<br>Comment: Unclear reasons for drop-out and unable to follow-up.
Selective reporting (reporting bias)	Low risk	Comment: the study protocol is available and all of the study's pre-specified (primary and secondary) outcomes that are of interest in the review have been reported in the pre-specified way.
Other sources of bias	Unclear risk	Funding from Sanofi-Aventis SpA, Milan, Italy. Materials for SMBG (glucose meters, strips, lancets, and control solutions) were supplied by LifeScan Inc., Milpitas, CA. Me.Te.Da. s.r.l., San Benedetto del Tronto, Italy, is the software company that developed the DID system.<br>Comment: insufficient rationale that a conflict of interests will introduce bias.

Charpentier 2011

**Methods**	Study design: multi-center parallel randomized clinical trial<br>Duration of study: 6 months

	Run-in time: 14 days<br>Clinic visit: 0, 3, 6 months<br>Setting: 17 hospital sites, From the Department of Diabetes and the Centre d'Études et de Recherche pour l'Intensification du Traitement du Diabète, Sud-Francilien Hospital, Corbeil-Essonnes, France; the Department of Endocrinology, University Hospital, Grenoble, France; the Department of Endocrinology, University Hospital, Besançon, France; the University Hospital Sainte Marguerite, Marseille, France; the Department of Endocrinology, CHU Bordeaux, Pessac, France; the Department of Diabetology, Toulouse Rangueil University Hospital, Toulouse, France; the Clinique d'Endocrinologie, Maladies Métaboliques et Nutrition, Institut du Thorax, Hôpital Laennec, Nantes, France; the Endocrinology Department, Centre Hospitalier Universitaire de Montpellier, Université de Montpellier, Montpellier, France; and the CIC-INSERM, Grenoble University Hospital, Grenoble, France.<br>Country: France
**Participants**	Identify: 17 hospital sites in France between September 2007 and April 2009.<br>Inclusion criteria: over 18 years old, had type 1 diabetes for at least 1 year, and had been treated with a basal bolus insulin regimen for at least 6 months, either with MDI or with a pump. Last HbA1c values during the year before and at entry of the study were >8.0% carry out at least two self-monitoring blood glucose (SMBG) everyday during the study.<br>Exclusion criteria: participation in a diabetes educational program within 3 months before the study or a clinical condition requiring the patient to receive follow-up more frequently than the quarterly visits scheduled.<br>Number of subjects: G1: baseline-61, 6 month-60; G2: baseline-60, 6 month-56; G3: baseline-59, 6 month-57.<br>Race: not reported<br>Education level: low level (college or less) 43 (23.9%); intermediate level (less than university degree) 38 (23.8%); high level (university degree) 99 (55.0%).
**Interventions**	Group 1-control group<br>Participants had no electronic logbook but kept their paper logbook and were asked to attend two follow-up visits at the hospital, after 3 and 6 months.<br>Group 2-Diabeo software<br>Home use of a smartphone recommending insulin doses with face-to-face follow-up visits at month 3 and month 6. Participant SMPG, diet, and insulin treatment data were automatically uploaded by the smartphone to a secured website. A bolus calculator with validated algorithms, taking into account SMPG level before meals, carbohydrate counts, and planned physical activity. Parameters personally tailored for adjustment of prandial and basal insulin dose are entered into the system for each patient. If fasting or postprandial SMPG do not meet target levels, the system can suggest adjustments for carbohydrate ratio, long-acting insulin analog dose, or pump basal rates. Diabeo software was edited by Voluntis (Paris, France), in collaboration with CERITD.<br>Group 3-Diabeo system + teleconsultations<br>Use of the smartphone with short teleconsultations every 2 weeks but no visit until point end. Teleconsultations were conducted with both patients and doctors in front of their computers or smartphone displaying last weeks' data and focused on insulin dose adjustments and motivational support. No follow-up hospital visits were scheduled but teleconsultations by telephone call were planned every 2 weeks.
**Outcomes**	Primary outcome: HbA1c<br>Secondary outcomes: the change in the HbA1c level from baseline to end point, the proportion of patients reaching the HbA1c target of below 7.5%, the change in SMPG frequency, the change in quality of life (QOL) and satisfaction assessed by Diabetes Health Profile and Diabetes QOL questionnaires, the amount of time spent by investigators conducting face-to-face visits or teleconsultations, and by the participants coming for hospital visits. For G2 and G3 participants, satisfaction with Diabeo system and their willingness to carry on with it at the end of the study was assessed by a specific questionnaire.<br>Adverse events: major hypoglycemia episodes, defined as requiring third-party assistance, and minor hypoglycemia episodes, defined as symptomatic, nonsevere hypoglycemia self-reported by the participant within 14 days before baseline and end point visits.<br>Outcomes of interest:

	HbA1c%:<br>G1: baseline 8.91±0.90, 6 month 9.10±1.16<br>G2: baseline 9.19±1.14, 6 month 8.63±1.07<br>G3: baseline 9.11±1.14, 6 month 8.41±1.04<br>Adverse events:<br>The frequency of symptomatic, non-severe hypoglycemia episodes: baseline 3.7±3.2, 6 month 4.6±4.0;<br>The participants experienced severe episodes during the 6 months of the study: G1 3, G2 3, G3 1
**Publication details**	Language: English<br>Funding: Voluntis provided the Diabeo software, and Orange (Paris, France) provided the smartphone and telephone lines; sanofi-aventis (Bridgewater, NJ) and CERITD funded the study.<br>Publication statues: peer reviewed journal

**Functions**		**Diabetes management modules**				
		**Monitoring**	**Medication management**	**Lifestyle modification**	**Complication prevention**	**Psychosocial care**
**Functional modules**	**Log**	Recording blood glucose;	Recording insulin dosages;	Recording diet and activity;	-	-
	**Structured display**	-				
	**General education**	-	-	-	-	-
	**Personalized feedback**	Customized blood glucose target;	Calculating bolus insulin dose based on algorithms;	-	-	-
	**Communication**	Teleconsultations between patients and doctors through video calls every two weeks;				

**Risk of bias**		
**Domain**	**Review authors' judgement**	**Support for judgement**
Random sequence generation (selection bias)	Low risk	Quote: "randomization was carried out using a Web-based system". Comment: block randomization was used.
Allocation concealment (selection bias)	Low risk	Quote: "randomization was carried out using a Web-based system". Comment: central allocation was used to conceal allocation.
Blinding of outcome assessment (detection bias, HbA1c)	Low risk	HbA1c is an objective measurement which is not likely to be influenced by whether or not assessors are blinded.
Blinding of outcome assessment (detection bias, adverse events)	Low risk	Major hypoglycemia episodes, defined as requiring third-party assistance, and minor hypoglycemia episodes, defined as symptomatic, non-severe hypoglycemia self-reported by the participant. These were objective measurements which were not likely to be influenced by whether or not assessors were blinded.
Incomplete outcome data (attrition bias) (HbA1c and adverse events)	Unclear risk	At 6 month, 1/61 patient in G1, 4/60 patient in G2, 2/59 in G3 were lost to follow-up. Missing values were replaced either by HbA1c measurements taken at month 6 in a private laboratory, provided the upper normal range limit was <6.0% (n = 6). If no result was available at month 6, HbA1c measures at month 3 were used (n = 5).

		Comment: reasons for missing data were not detailed reported.
Selective reporting (reporting bias)	Low risk	Comment: the study protocol is available and all of the study's pre-specified (primary and secondary) outcomes that are of interest in the review have been reported in the pre-specified way.
Other sources of bias	Unclear risk	Comment: insufficient rationale that a conflict of interests (funding) will introduce bias.

Rossi 2010

**Methods**	Study design: multicenter parallel randomized controlled trial.<br>Duration of study: 6 month.<br>Run-in time: 2 weeks.<br>Clinic visit: 0, 3, 6 months.<br>Setting: seven Diabetes Outpatient Clinics: three in Italy, two in England, and two in Spain.<br>Country: Italy
**Participants**	Identify: Every center was asked to enroll 20 patients<br>Inclusion criteria: diagnosis of type 1 diabetes, age 18 years, no previous education on carbohydrate counting, and treatment with multiple daily injections of short- acting and long-acting insulin analogs or with continuous subcutaneous insulin in- fusion; patients practiced self-monitoring of blood glucose at least three times a day. Other important requirements in the selection of patients were adequate familiarity in the use of mobile phones, according to the physician judgment, and possession of a personal mobile phone card.<br>Exclusion criteria: if they were being treated with NPH insulin or soluble regular insulin, had an eating disorder, were pregnant, were unable to send or receive short text messages, were unable or unwilling to give informed con- sent, or had any other disease or condition that may interfere with compliance with the protocol or completion of the study.<br>Number of subjects: I: baseline-67, 6 month-58; C: baseline-63, 6 month-61.<br>Race: not reported.<br>Education level: not reported.
**Interventions**	Intervention group-a Diabetes Interactive Diary (DID)<br>A carbohydrate/insulin bolus calculator, an information technology device, and a telemedicine system based on the communication between a health care professional (physician or dietitian) and a patient via text messages. It supports patients in managing the CHO counting through a food atlas and in recording the self-monitoring blood glucose (SMBG) measurements. On the basis of the stored data (blood glucose values deriving from self-monitoring, individualized correction factor, and insulin: CHO ratio set by the physician, food intake, and physical activities performed), DID suggests the daily carbohydrate intake, and automatically calculates the most appropriate insulin dose to be injected at each meal. All the recorded data are sent to the physician via SMS and reviewed on the personal computer of the diabetes clinic. Then, any new therapeutic and behavioral prescription can be sent from the diabetes clinic computer to the patient's mobile phone.<br>Control group-standard carbohydrate counting<br>All participants were instructed to measure their blood glucose levels at least seven times a week (three or more times fasting, three or more times postprandially, and once or more at bedtime). Each patient was advised to use their own glucometer. Average caloric consumption by exercise was estimated at clinic visits.
**Outcomes**	Primary outcome: HbA1c<br>Second outcome: changes in fasting blood glucose (FBG) levels, body weight, lipid profile, blood pressure, safety- related problems (frequency of hypoglycemic episodes and hospitalizations), differences in time dedicated to educational activities, quality of life, patient treatment satisfaction.<br>Adverse events: the WHO-DTSQ two items are treated individually and explore the perceived frequency of hyperglycemic and

	hypoglycemic episodes.<br>Outcomes of interest:<br>HbA1c%:<br>Intervention group: baseline 8.2±0.8, 6 month 7.8±0.8, change -0.4±0.9,<br>Control group: baseline 8.4±0.7, 6 month 7.9±1.1, change -0.5±1,<br>Adverse events:<br>No patients in either group were admitted to the hospital during the study, and none reported any severe hypoglycemic episode requiring assistance. In each group, 2 patients reported episodes of mild hypoglycemia.
**Publication details**	Language: English<br>Funding: Me.Te.Da. and Lifescan, Milpitas, CA. G.V. is a medical consultant for Me.Te.Da.<br>Publication statues: peer reviewed journal

**Functions**		**Diabetes management modules**				
		**Monitoring**	**Medication management**	**Lifestyle modification**	**Complication prevention**	**Psychosocial care**
**Functional modules**	**Log**	Recording blood glucose;	Recording insulin dosages;	Carbohydrate counting; Recording food intake and physical activities;	-	-
	**Structured display**	-				
	**General education**	-	-	-	-	-
	**Personalized feedback**	-	Calculating insulin dose based on algorithms;	Suggestions of the daily carbohydrate intake;	-	-
	**Communication**	Communication with the physician or dietitians via text messages;				

**Risk of bias**		
**Domain**	**Review authors' judgement**	**Support for judgement**
Random sequence generation (selection bias)	Low risk	Quote: "block randomization was used to assign each patient."<br>Comment: block randomization was used
Allocation concealment (selection bias)<br>Low risk	Quote: "randomization was performed through a telephone call to the coordinating center".<br>Comment: central allocation was used to conceal allocation.	
Blinding of outcome assessment (detection bias, HbA1c)	Low risk	HbA1c is an objective measurement which is not likely to be influenced by whether or not assessors are blinded.
Blinding of outcome assessment (detection bias, adverse events)	Low risk	Serious hypoglycemic episode was defined as those requiring medical intervention, which was an objective measurement. The assessment of hypoglycemic episode was not likely to be influenced by whether or not assessors were blinded.
Incomplete outcome data (attrition bias)	High risk	In the intervention group, 1 lost to follow-up, 8 discontinued

(HbA1c and adverse events)		intervention: 4 not compliant with DID or visit scheduling, and 4 for technical difficulties in transmitting messages; In the control group, 2 lost to follow-up.<br>Comment: technical difficulties should be analyzed in adverse events.
Selective reporting<br>(reporting bias)	High risk	Comment: technical difficulties should be analyzed in adverse events.
Other sources of bias	Low risk	Comment: the study appears to be free of other sources of bias.

Yoo 2009

**Methods**	Study design: parallel randomized controlled trial.<br>Duration of study: 3 month<br>Run-in time: not reported.<br>Clinic visit: 0, 3 months.<br>Setting: University hospital setting (Korea University) and Community healthcare centre (Guro-Gu Public Health Centre)<br>Country: Korea
**Participants**<br>Inclusion criteria:<br>Between 30 and 70 years of age, who met the following criteria: (i) a diagnosis of both type 2 diabetes and hypertension at least 1 year previously by a physician; (ii) HbA1c 6.5%-10.0%; (iii) blood pressure > 130/80 mmHg; and (iv) BMI≥23.0 kg/m^2 (overweight according to Asia-Pacific criteria)<br>Exclusion criteria:<br>i) severe diabetic complications (e.g. diabetic foot or severe diabetic retinopathy); (ii) liver dysfunction with aspartate aminotransferase or alanine amino-	

transferase>2.5 times the reference level, or renal dysfunction (serum creatinine>132 mmol/L); (iii) medical history of congestive heart failure, angina pectoris, MI, or stroke based on a physician's diagnosis; (iv) pregnancy or lactation; or (v) other medical problems that could affect study results or trial participation or (Vi) excluded all participants with hsCRP≥15.0 mg to rule out any occult inflammatory or infectious disorders<br>Number of subjects: I baseline-62, 3 month-57; C baseline-61, 3 month-54.<br>Race: not reported<br>Education level: not reported	
**Interventions**	Intervention group-UCDC<br>A Ubiquitous Chronic Disease Care (UCDC) system had a cellular phone(LG-SV280;LGElectronics, Seoul,Korea)with a modular blood glucose measuring device (Anycheck; Insung Information Co., Seoul, Korea), an automatic blood pressure monitoring device (T5M; Omron, Kyoto, Japan), as well as body weight scales (HD308; Tanita, Tokyo, Japan). UCDS using cellular phones to provide continuous education, reinforcement of diet, exercise, and SMBG. First, the UCDC system sent out an alarm on the cellular phone to remind the participant to measure their blood glucose, blood pressure twice a day (before breakfast and bedtime) and body weight once a day (before breakfast) and generated messages of encouragement, reminders, and recommendations. For example, your fasting blood glucose level is very high compared with the appropriate target level for Type 2 diabetes (< 7.2 mmol/L). If this high level recurs often, diabetic complications might result. Reduce your calorie intake and avoid foods high in fat. In addition, plan for regular exercise after your meals. Second, the system automatically recorded participant's exercise time. Third, participants received information three times a day regarding healthy diet and exercise methods, along with general information about

	diabetes, hypertension and obesity.<br>Control group-conventional clinic visits<br>Visited their clinic according to their routine schedule and received the usual out-patient treatment from their physicians during the study period. During the trial, drug dosage was not changed in either the UCDC or the control groups at either location.
**Outcomes**	Outcomes: BMI, plasma glucose, lipid profile (serum total cholesterol, triglycerides, and high-density lipoprotein cholesterol), HbA1c.<br>Adverse events: not reported<br>Outcomes of interest:<br>HbA1c%:<br>Intervention group: baseline-7.6±0.9, 3 month-7.1±0.8;<br>Control group: baseline-7.4±0.9, 3 month-7.6±1.0
**Publication details**	Language: English<br>Funding: Seoul R & BD Project. The development of the HSA business model and technology was sponsored by the Ministry of Commerce, Industry and Energy Publication statues: Peer reviewed journal

**Functions**		**Diabetes management modules**				
		**Monitoring**	**Medication management**	**Lifestyle modification**	**Complication prevention**	**Psychosocial care**
**Functional modules**	**Log**	Recording blood glucose and blood pressure;	-	Track diet and exercise;	-	-
	**Structured display**	-				
	**General education**	-	-	Education of healthy diet and exercise methods;	-	-
	**Personalized feedback**	Reminders of monitoring; Target setting;	-	Advice on lifestyle modification;	-	-
	**Communication**	-				

**Risk of bias**		
**Domain**	**Review authors' judgement**	**Support for judgement**
Random sequence generation (selection bias)	Unclear risk	Quote: "We recruited patients for this open-label, randomized, controlled, prospective study from both a university hospital setting"<br>Comment: Insufficient information provided
Allocation concealment (selection bias)	Unclear risk	Comment: insufficient information provided.
Blinding of outcome assessment (detection bias) (HbA1c)	Low risk	HbA1c is an objective measurement which is not likely to be influenced by whether or not assessors are blinded.
Incomplete outcome data (attrition bias)<br>All outcomes	Unclear risk	Quote: "Five patients (8.1%) dropped out of the intervention group and seven (10%) out of the control group. The characteristics of patients who did and did not drop out were similar in both the intervention and control groups"<br>Comment: no details provided about reasons for patients dropping out. No imputation of data or intention-to-treat analysis reported. Insufficient evidence to permit judgement.
Selective reporting	High risk	Comment: the study report fails to include adverse events for a key

(reporting bias)		outcome that would be expected to have been reported for such a study.
Other sources of bias	Low risk	Comment: the study appears to be free of other sources of bias.

Istepanian 2009

**Methods**	Study design: parallel randomized controlled trial.<br>Duration of study: 9 month<br>Run-in time: 4 weeks.<br>Clinic visit: 0, 9 months.<br>Setting: the Thomas Addison Diabetes Unit of St George's Hospital.<br>Country: UK
**Participants**	Inclusion criteria: Ambulant patients aged over 18 years with diabetes.<br>Exclusion criteria: a physical inability to self-monitor blood glucose, pregnancy, severe life- threatening or terminal illness or an inability to provide written informed consent.<br>Number of subjects: I baseline-72; C baseline-65.<br>Race: Caucasian 47(34.3%), African-Caribbean 42(30.7%), Indo-Asian 42(30.7%), other 6(4.4%).<br>Education level: not reported
**Interventions**	Intervention group-mobile health technology<br>Patients were trained to measure their blood glucose with a sensor which transmitted the readings to a mobile phone via a Bluetooth wireless link. Clinicians were then able to examine and respond to the readings which were viewed with a web-based application. Letters were sent from the clinician to the patients and their general practitioners with details of the amalgamated readings and treatment recommendations. Patients could also use the mobile phones free of charge to contact the research team for clinical and technical support.<br>Control group-usual care<br>received care with their usual doctor in the outpatient and/or primary care setting.
**Outcomes**	Primary outcomes: HbA1c.<br>Adverse events: not reported.<br>Outcomes of interest:<br>HbA1c%:<br>Intervention group: baseline-7.9±1.5, 3 month-7.76;<br>Control group: baseline-8.1±1.6, 3 month-8.40.
**Publication details**<br>Language: English<br>Funding: the IDEN Group, Motorola, USA and the Motohealth team in UK.<br>Publication statues: Peer reviewed journal	

**Functions**	**Diabetes management modules**

		Monitoring	Medication management	Lifestyle modification	Complication prevention Psychosocial care	
Functional modules	Log	Recording blood glucose;	-	-	-	-
	Structured display	-				
	General education	-	-	-	-	-
	Personalized feedback	-	-	-	-	-
	Communication	Connections with the research team for clinical support through telephone calls if necessary;				

Risk of bias		
**Domain**	**Review authors' judgement**	**Support for judgement**
Random sequence generation (selection bias)	Low risk	Quote: "Randomization to usual care or the telemonitoring arm of the study was by computer-generated random numbers".
Allocation concealment (selection bias)	Unclear risk	Comment: insufficient information provided.
Blinding of outcome assessment (detection bias) (HbA1c)	Low risk	HbA1c is an objective measurement which is not likely to be influenced by whether or not assessors are blinded.
Incomplete outcome data (attrition bias)(HbA1c)	Unclear risk	Comment: no details provided about reasons for patients dropping out.
Selective reporting (reporting bias)	High risk	Comment: the study report fails to include adverse events for a key outcome that would be expected to have been reported for such a study.
Other sources of bias	Low risk	Comment: the study appears to be free of other sources of bias.

Quinn 2008

**Methods**	Study design: parallel randomized controlled trial.<br>Duration of study: 3 month<br>Run-in time: not reported.<br>Clinic visit: 0, 3 months.<br>Setting: one community endocrinology and two community primary care practices in Maryland.<br>Country: USA
**Participants**	Inclusion criteria:<br>the study enrolled patients 18-70 years old who had a diagnosis of type 2 diabetes for at least 6 months. Study patients were required to have an A1c 7.5% and to have been on a stable diabetes therapeutic regimen for 3 months prior to study enrolment.<br>Exclusion criteria: none stated.<br>Number of subjects: I: baseline-15, 3 month-13; C baseline-15, 3 month-13.<br>Race: African American: I 10, C 6; White (non-Hispanic): I 3, C 7.<br>Education level: not reported.
**Interventions**	Intervention group-WellDoc's proprietary Diabetes Manager software<br>Study patients enrolled in the intervention group received a Bluetooth® (Bluetooth SIG, Bellevue, WA)-enabled One Touch Ultra BG meter and a Nokia (Espoo, Finland) 6682™ or 6680™ cell phone equipped with WellDoc's proprietary DiabetesManager software. The software provided real-time feedback on patients' blood glucose levels, displayed patients' medication regimens,

	incorporated hypo- and hyperglycemia treatment algorithms. Patient data captured and transferred to secure servers were analyzed. Patient's BG value would be sent to the patient's cell phone. Patient data were uploaded from the web server into the cell phone and integrated into the cell phone- based software, DiabetesManager, for personalized feedback.<br>Once the BG value was received by the phone, the DiabetesManager application on the cell phone was triggered. The software asked the patient to identify (label) the BG (e.g., "Before Breakfast?," "Bedtime?"). Another major component of the system , Guided Compliance, directed patients to test their BG at optimal times to generate BG data points that could be used for a pattern analysis.<br>Once the BG was labeled, the patient was given feedback about the value related to the patient-specific target level and was shown his or her HCP-prescribed medication instructions. If the patient's BG levels were above or below his or her target levels, the patient was given real-time feedback on how to correct the BG level. The patient was then prompted to enter the medication dosage he or she actually took and the number of carbohydrates eaten, if known.<br>When a troubling BG value or pattern was detected, the patient either was directed to test (at particular times of the day to generate a pattern analysis) or e-mailed several questions in attempt to discover the root of the issue. Once the problem was identified, the patient was sent an e- mail with educational material specific to that issue. WellDoc communicates suggested medication changes directly to patients, and all suggested changes to patients' therapy regimes are communicated to the HCP. The choice to implement or not implement WellDoc's recommendations is at the HCPs' discretion.<br>Control group- SMBG<br>They were asked to fax or call in their BG logbooks every 2 weeks to their HCPs until their BG levels were stabilized in the target ranges or until their HCPs changed testing frequency. Investigators asked treating HCPs to follow their usual standards of care for the patients' diabetes management.
**Outcomes**	Primary outcomes: HbA1c<br>Secondary outcomes: summary of Diabetes Self-Care Activities (SDSCA) questionnaire. PCP prescribing practice.<br>Adverse events: not reported<br>Outcome of interest:<br>HbA1c%:<br>Intervention group: baseline-9.51, 3 month-7.48;<br>Control group: baseline-9.05, 3 month-8.37.
**Publication details**	Language: English<br>Funding: Study was supported by LifeScan, Inc. and Nokia, Inc.<br>Publication statues: Peer reviewed journal

**Functions**		**Diabetes management modules**				
		**Monitoring**	**Medication management**	**Lifestyle modification**	**Complication prevention**	**Psychosocial care**
**Functional modules**	**Log**	Recording blood glucose;	Recording medication regimens;	Recording diet;	-	-
	**Structured display**	Labels; Blood glucose pattern analysis;				
	**General education**	-	-	-	Educational material about hypoglycemia and hyperglycemia treatment;	-

	**Personalized feedback**	Target and feedback on blood glucose levels;	-	-	-	-
	**Communication**	In-app call to reach HCP every 2 weeks;				

**Risk of bias**		
**Domain**	**Review authors' judgement**	**Support for judgement**
Random sequence generation (selection bias)	Unclear risk<br>Quote: "Eligible patients gave consent and were randomized to either the control or intervention group<br>Comment: insufficient information provided	
Allocation concealment (selection bias)	Unclear risk	Comment: insufficient information provided
Blinding of outcome assessment (detection bias) (HbA1c)	Low risk<br>HbA1c is an objective measurement which is not likely to be influenced by whether or not assessors are blinded.	
Incomplete outcome data (attrition bias)(HbA1c)	Unclear risk	Comment: no details given about reasons for dropping out of study. Insufficient information provided
Selective reporting<br>(reporting bias)	High risk	Comment: the study report fails to include adverse events for a key outcome that would be expected to have been reported for such a study.
Other sources of bias	Unclear risk<br>"A convenience sample of 30 patients with type 2 diabetes was recruited"<br>Comment: Small convenience sample.	
