# Supplementary figures and images for: Mobile App-Based Interventions to Support Diabetes Self-Management: A Systematic Review of Randomized Controlled Trials to Identify Functions Associated with Glycemic Efficacy
Source: JMIR Mhealth Uhealth. 2017 Mar 14;5(3):e35. doi: 10.2196/mhealth.6522 (PMC5373677; doi:10.2196/mhealth.6522)

## Multimedia Appendix 6 Funnel plot

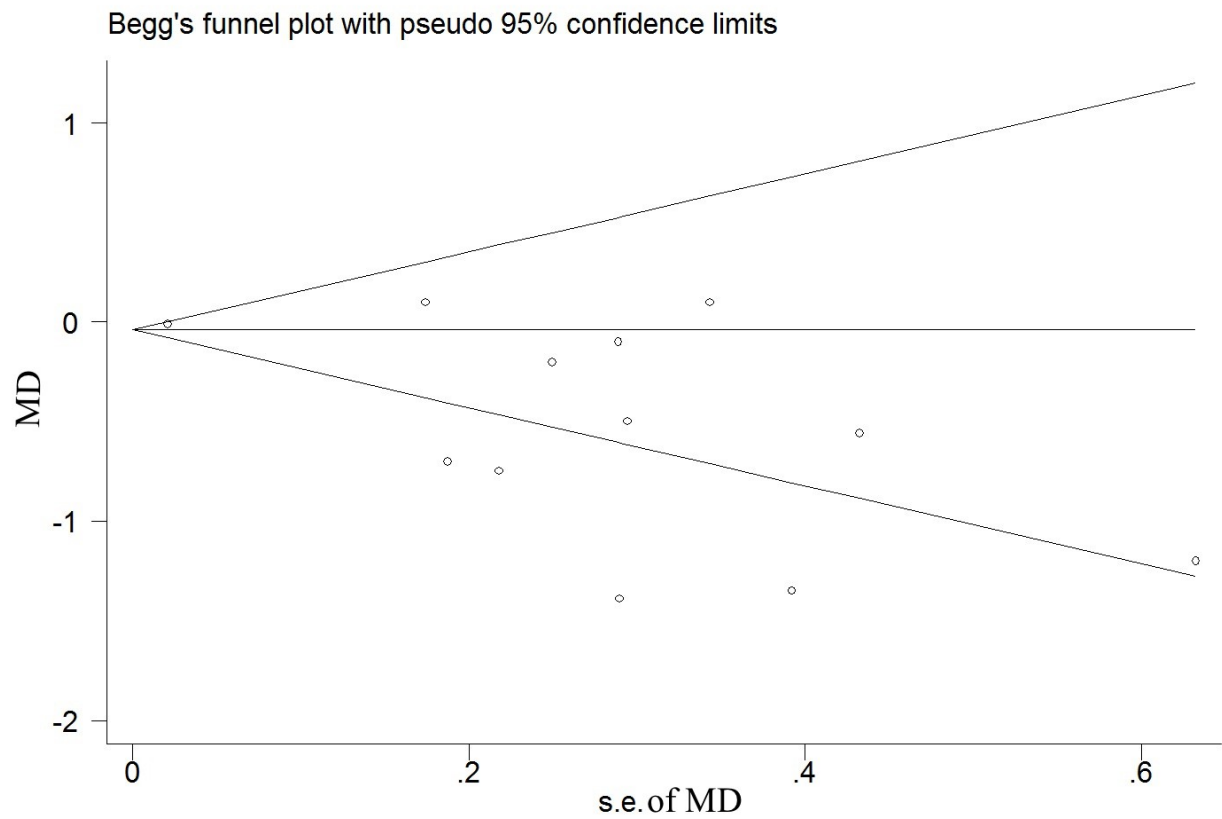

s.e., standard error. MD, Mean Difference.

Supplement: Multimedia Appendix 5 [file mhealth_v5i3e35_app5.pdf]
