# Supplementary material for: Mobile App-Based Interventions to Support Diabetes Self-Management: A Systematic Review of Randomized Controlled Trials to Identify Functions Associated with Glycemic Efficacy
Source: JMIR Mhealth Uhealth. 2017 Mar 14;5(3):e35. doi: 10.2196/mhealth.6522 (PMC5373677; doi:10.2196/mhealth.6522)
Supplement: Multimedia Appendix 6 [file mhealth_v5i3e35_app6.pdf]

Multimedia Appendix 7 GRADE for primary and secondary outcomes

**Mobile health interventions for diabetes management**				
**Patient or population:** Outpatients with diabetes mellitus<br>**Settings:** The management of diabetes in outpatients<br>**Intervention:** App-based mobile health interventions<br>**Comparison:** standard diabetes care, usual diabetes care, standard paper diabetes diary, standard SMBG, standard self-care, conventional diabetes patient education, standard carbohydrate counting conventional clinic visits				
**Outcomes**	**Effects of mobile health apps for diabetes management**	**No of Participants (studies)**	**Quality of the evidence (GRADE)**	**Comments**
HbA1c changes [follow-up: 3-12 months]	The mean difference (MD) for HbA_{1c} changes of app-based mobile health interventions versus usual care was 0.48% (95% CI 0.19 to 0.77).	974<br>(12 stuides)	⊕⊕⊝⊝<br>**low**[a]	All included trials suffered from more than one risk of bias (lack of allocation concealment, lack of blinding, incomplete outcome data and selective reporting).
Severe hypoglycemia [follow-up: 3-12 months] The risk ratio (RR) for severe hypoglycemia of mobile health versus usual care was 1.07 (95% CI 0.23 to 5.09)	346<br>(4 studies)	⊕⊕⊝⊝<br>**low**[b]	CI for severe hypoglycemia was wide and included null effect.	
Adverse events [follow-up: 3-12 months] One study announced no adverse events had been identified,	458<br>(5 studies)	⊕⊝⊝⊝<br>**very low**[c]	Variations in definitions, reporting formats and the level of supplied detail.	

another study announced no adverse clinical event but several undesired technical events in automatic data transmission between glucometer and apps. Five studies reported subjects, proportion of subjects, or incidence of severe hypoglycemia. Three studies reported subjects, proportion of subjects, frequency, or incidence of hypoglycemia. None of the studies reported any other kind of adverse events, or any death in participants.				
**CI:** Confidence interval.				

GRADE Working Group grades of evidence
High quality: Further research is very unlikely to change our confidence in the estimate of effect.
Moderate quality: Further research is likely to have an important impact on our confidence in the estimate of effect and may change the estimate.

Low quality: Further research is very likely to have an important impact on our confidence in the estimate of effect and is likely to change the estimate.
Very low quality: We are very uncertain about the estimate.

[a]Downgraded by two levels owing to the potential publication bias and study limitations (lack of allocation concealment, lack of blinding of participants and personnel, incomplete outcome data, selective reporting and other bias as shown in figure 2 and figure 3).

[b]Downgraded by two level owing to imprecision (wide confidence intervals include null effect) and study limitations (risk of bias of four trials).

[c]Downgraded by three level owing to inconsistency (substantial diversity in outcome measures definition), imprecision (small sample sizes and low event rates) and study limitations (risk of bias of five trials).
