# Supplementary material for: Mobile App-Based Interventions to Support Diabetes Self-Management: A Systematic Review of Randomized Controlled Trials to Identify Functions Associated with Glycemic Efficacy
Source: JMIR Mhealth Uhealth. 2017 Mar 14;5(3):e35. doi: 10.2196/mhealth.6522 (PMC5373677; doi:10.2196/mhealth.6522)

Multimedia Appendix 8 Meta-analyses for adverse events

A Severe hypoglycemia

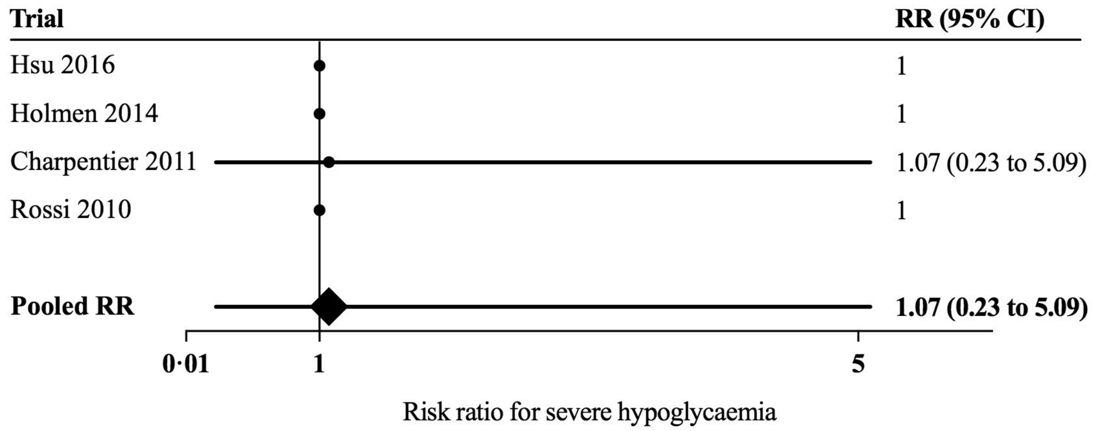

B Overall hypoglycemia

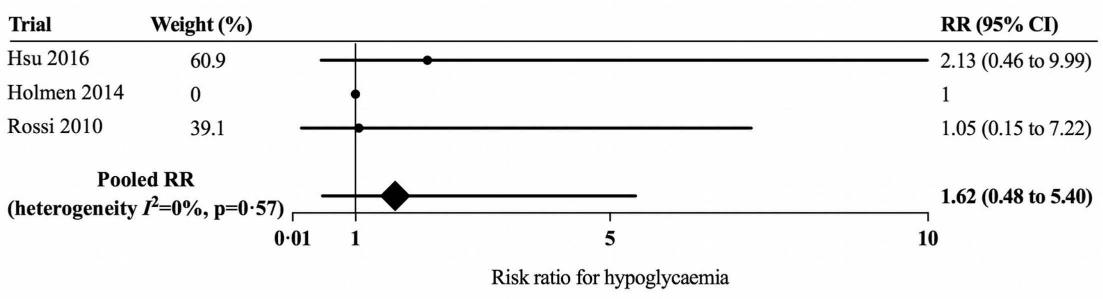

Supplement: Multimedia Appendix 7 [file mhealth_v5i3e35_app7.pdf]
