# Supplementary material for: Mobile App-Based Interventions to Support Diabetes Self-Management: A Systematic Review of Randomized Controlled Trials to Identify Functions Associated with Glycemic Efficacy
Source: JMIR Mhealth Uhealth. 2017 Mar 14;5(3):e35. doi: 10.2196/mhealth.6522 (PMC5373677; doi:10.2196/mhealth.6522)
Supplement: Multimedia Appendix 8 [file mhealth_v5i3e35_app8.pdf]

Multimedia Appendix 10. Classifications of the patient-clinician communication function

First author and year	Intervention	Frequency	Method	Delivery personnel	Intensity of communications
Hsu 2016	Cloud-based diabetes management program	Weekly	Secure text messages and virtual visits	Healthcare provider	The streamlined communications tools were integrated into the application. The shared decision-making interfaces charts to help the subjects and HCPs.
Baron 2016	Mobile telehealth (MTH)	NR	Web portal	Nurse	The MTH nurses accessed the data transmitted to the server via a Web portal also accessible to MTH patients who supported insulin titration.
Katalenich 2015	Diabetes Remote Monitoring and Management System (DRMS)	Hyper- or hypo-glycaemia emergency	In-app connection	Endocrino logist	When a participant submitted extreme values of blood glucose, the patient was immediately and automatically connected to the endocrinologist on call.
Holmen 2014	Few Touch application (FTA)	5 telephone calls in 4 months	Telephone calls	Nurse	The participants received 5 telephone calls from the nurse during the first 4 months, each of which lasted for an average of 20 minutes. A schedule for each conversation was developed before the study by an interdisciplinary research team.
Waki 2014	DiaBetics	If necessary	Telephone call	Physician	Readings defined as abnormal—blood glucose above 400 mg/dl or below 40 mg/dl, and systolic blood pressure above 220 mmHg—are reported to a doctor as “Dr Call,” meaning a physician will check the data and interact with the patient if necessary.
Rossi 2013	Diabetes Interactive Diary	NR	Text messages	Physician	In-app communication between patient and physician via text messages.
Charpentier 2011	Diabeo (on market app)	Every two weeks;	Video calls	Doctor	Teleconsultations were conducted with both patients and doctors in front of their computers or smartphone displaying last weeks’ data and focused on insulin dose

					adjustments and motivational support;
Rossi 2010	Diabetes Interactive Diary	NR	Text messages	Physician and dietitian	A telemedicine system based on the communication between a health care professional (physician or dietitian) and a patient via text messages.
Istepanian 2009	Mobile health technology	If necessary	Telephone call	Research team	Patients could use the mobile phones to contact the research team for clinical and technical support.
Quinn 2008	WellDoc's Diabetes Manager software	Every 2 weeks	In-app connection	Healthcare provider	In-app call to reach their HCP.
